# Supplementary material for: An unbiased template of the Drosophila brain and ventral nerve cord
Source: PLoS One. 2020 Dec 31;15(12):e0236495. doi: 10.1371/journal.pone.0236495 (PMC7774840; doi:10.1371/journal.pone.0236495)
Supplement: S1 File — (PDF) [file pone.0236495.s001.pdf]

1 Supplementary Tables

Template	Algorithm	Mean	Std dev	10th perc	median	90th perc	N
JFRC2010	ANTs A	8.622	6.716	1.581	7.036	17.847	256308
	ANTs B	4.956	3.951	1.225	3.808	10.075	146800
	ANTs C	5.196	3.865	1.414	4.243	10.223	146628
	CMTK A	8.446	8.462	1.581	5.788	17.958	118112
	CMTK B	7.776	7.473	1.581	5.431	16.654	119994
	CMTK C	7.686	7.710	1.732	5.523	15.133	137414
	Elastix A	5.452	4.060	1.414	4.528	10.794	141868
	Elastix B	7.431	5.954	1.581	5.745	15.764	122728
JFRC2013	ANTs A	4.568	3.730	1.000	3.606	9.247	138368
	ANTs B	6.720	4.943	1.581	5.523	13.675	128144
	ANTs C	6.563	5.076	1.581	5.196	13.528	133550
	CMTK A	5.413	4.345	1.225	4.183	11.180	130310
	CMTK B	5.513	4.335	1.225	4.359	11.380	135338
	CMTK C	6.112	4.696	1.414	5.000	12.490	141378
	Elastix A	5.079	3.812	1.414	4.123	9.950	135046
	Elastix B	5.653	4.511	1.414	4.472	11.533	132788
JRC2018	ANTs A	4.659	3.824	1.000	3.606	9.513	140804
	ANTs B	4.925	3.849	1.225	3.873	9.721	135624
	ANTs C	5.311	4.045	1.414	4.301	10.464	141770
	CMTK A	5.000	4.021	1.225	3.873	10.025	124912
	CMTK B	4.913	3.969	1.225	3.808	9.849	124664
	CMTK C	4.834	3.885	1.225	3.808	9.667	128432
	Elastix A	4.972	3.857	1.225	4.062	9.721	130008
	Elastix B	5.796	4.684	1.414	4.528	12.062	119008
FCWB	ANTs A	5.500	4.759	1.000	4.000	12.124	128116
	ANTs B	8.149	5.519	2.121	7.036	16.093	103628
	ANTs C	7.903	5.370	2.000	6.819	15.330	113236
	CMTK A	7.257	5.367	1.581	5.874	15.182	105712
	CMTK B	6.848	5.312	1.581	5.339	14.916	109638
	CMTK C	6.666	4.872	1.581	5.431	13.656	125260
	Elastix A	8.217	5.590	2.000	7.106	16.340	122506
	Elastix B	9.228	6.644	2.236	7.649	18.722	118268
Tefor	ANTs A	4.641	3.842	1.000	3.606	9.539	141758
	ANTs B	4.824	3.725	1.225	3.808	9.747	143886
	ANTs C	5.033	3.597	1.414	4.183	9.849	165882
	CMTK A	7.425	5.160	1.581	6.364	14.933	140012
	CMTK B	6.941	4.928	1.581	5.874	13.946	136286
	CMTK C	5.212	4.221	1.000	4.000	11.091	205034
	Elastix A	5.800	4.444	1.414	4.583	12.104	126216
	Elastix B	8.726	6.750	1.732	7.000	18.152	101056

Table S1. CRE_R : crepine

Template	Algorithm	Mean	Std dev	10th perc	median	90th perc	N
JFRC2010	ANTs A	7.403	6.977	1.581	4.950	18.221	16290
	ANTs B	4.236	2.557	1.414	3.808	7.583	30124
	ANTs C	4.126	2.587	1.225	3.606	7.416	37836
	CMTK A	6.262	6.624	1.225	4.062	15.000	35436
	CMTK B	5.936	6.301	1.225	3.808	14.916	30950
	CMTK C	6.016	5.701	1.581	4.183	14.933	33842
	Elastix A	4.645	3.253	1.225	3.808	9.028	14486
	Elastix B	4.522	3.060	1.225	3.808	8.868	14868
JFRC2013	ANTs A	3.932	2.799	1.000	3.240	7.649	21630
	ANTs B	3.346	2.276	1.000	2.915	6.325	47016
	ANTs C	3.153	2.230	0.707	2.646	6.000	44028
	CMTK A	3.529	2.666	1.000	2.915	6.856	24354
	CMTK B	3.556	2.687	1.000	2.915	6.964	24016
	CMTK C	4.098	2.588	1.225	3.606	7.583	34654
	Elastix A	3.719	2.763	1.000	3.000	7.382	23464
	Elastix B	4.062	2.963	1.000	3.240	8.093	21682
JRC2018	ANTs A	3.768	2.749	1.000	3.000	7.382	21558
	ANTs B	4.132	2.791	1.000	3.606	7.649	23310
	ANTs C	4.038	2.633	1.225	3.536	7.416	26778
	CMTK A	3.868	2.804	1.000	3.162	7.616	21388
	CMTK B	3.908	2.907	1.000	3.082	7.842	21008
	CMTK C	3.809	2.839	1.000	3.082	7.550	21214
	Elastix A	3.924	2.821	1.000	3.240	7.810	21484
	Elastix B	4.162	2.984	1.000	3.536	8.185	19802
FCWB	ANTs A	3.755	2.665	1.000	3.082	7.246	22464
	ANTs B	3.844	2.335	1.225	3.536	6.745	26150
	ANTs C	3.759	2.273	1.225	3.464	6.708	22568
	CMTK A	3.814	2.649	1.000	3.240	7.280	46938
	CMTK B	3.866	2.702	1.000	3.240	7.382	31124
	CMTK C	4.068	2.855	1.000	3.536	7.906	33454
	Elastix A	3.782	3.067	1.000	3.240	6.856	39318
	Elastix B	4.687	3.322	1.000	3.808	9.618	56956
Tefor	ANTs A	4.258	2.985	1.225	3.606	8.185	19428
	ANTs B	4.267	2.671	1.414	3.808	7.649	24896
	ANTs C	4.942	2.938	1.581	4.528	8.631	30864
	CMTK A	4.763	3.168	1.414	4.123	9.274	23110
	CMTK B	4.860	3.102	1.414	4.243	9.343	23078
	CMTK C	4.490	3.093	1.414	3.808	8.544	21146
	Elastix A	4.494	2.952	1.225	3.808	8.399	15300
	Elastix B	4.062	2.450	1.225	3.606	7.382	18314

Table S2. CAN_R : cantle

Template	Algorithm	Mean	Std dev	10th perc	median	90th perc	N
JFRC2010	ANTs A	7.150	7.956	1.581	4.528	19.770	225304
	ANTs B	5.234	4.993	1.581	4.183	8.746	187944
	ANTs C	5.416	5.060	1.581	4.359	9.165	193640
	CMTK A	5.177	4.705	1.414	4.123	9.192	189018
	CMTK B	5.022	4.600	1.414	4.062	8.602	194876
	CMTK C	5.253	5.016	1.581	4.183	8.746	187834
	Elastix A	4.623	4.374	1.414	3.674	7.810	187814
	Elastix B	4.646	4.339	1.414	3.808	7.842	198088
JFRC2013	ANTs A	4.671	4.553	1.225	3.674	7.906	194144
	ANTs B	6.253	6.122	1.581	4.743	11.180	163990
	ANTs C	6.093	6.197	1.581	4.528	11.269	159530
	CMTK A	5.040	4.726	1.581	4.062	8.631	178554
	CMTK B	5.070	4.724	1.414	4.062	8.660	172350
	CMTK C	5.755	4.961	1.581	4.528	10.440	139908
	Elastix A	4.571	4.670	1.225	3.606	7.649	192020
	Elastix B	4.659	4.590	1.414	3.674	7.810	187546
JRC2018	ANTs A	4.582	4.503	1.225	3.606	7.649	196510
	ANTs B	4.853	4.557	1.414	3.873	8.155	200496
	ANTs C	5.003	4.938	1.414	3.873	8.515	220340
	CMTK A	4.663	4.622	1.225	3.674	7.842	192616
	CMTK B	4.673	4.505	1.414	3.674	7.906	190402
	CMTK C	4.754	4.397	1.414	3.808	8.031	193484
	Elastix A	4.570	4.344	1.414	3.674	7.616	196662
	Elastix B	4.712	4.633	1.414	3.808	7.906	190378
FCWB	ANTs A	4.766	4.218	1.414	3.873	8.276	198206
	ANTs B	5.606	5.450	1.581	4.472	9.539	118958
	ANTs C	5.231	4.522	1.581	4.183	9.247	129264
	CMTK A	5.296	4.588	1.581	4.359	9.301	189414
	CMTK B	5.277	4.672	1.581	4.301	9.220	187246
	CMTK C	5.168	4.630	1.581	4.243	8.746	153568
	Elastix A	5.405	6.600	1.414	4.123	8.631	197108
	Elastix B	4.817	5.207	1.414	3.808	8.000	201070
Tefor	ANTs A	4.629	4.464	1.225	3.674	7.810	195792
	ANTs B	4.936	4.866	1.414	3.808	8.544	206934
	ANTs C	5.295	5.410	1.581	4.183	8.718	221890
	CMTK A	5.096	4.741	1.414	3.873	9.028	198934
	CMTK B	4.896	4.528	1.414	3.873	8.515	203194
	CMTK C	5.165	5.229	1.581	4.123	8.515	201560
	Elastix A	4.603	4.346	1.414	3.674	7.746	187738
	Elastix B	4.745	4.064	1.414	3.873	8.093	197718

Table S3. AMMC_R : antennal mechanosensory and motor center

Template	Algorithm	Mean	Std dev	10th perc	median	90th perc	N
JFRC2010	ANTs A	8.835	7.213	1.414	6.745	19.506	501078
	ANTs B	3.167	2.986	0.707	2.236	6.671	316016
	ANTs C	3.315	3.202	0.707	2.345	7.106	307394
	CMTK A	4.211	5.414	0.707	2.236	11.203	293368
	CMTK B	4.158	5.566	0.707	2.236	9.539	289230
	CMTK C	4.177	4.478	0.707	2.828	9.220	331886
	Elastix A	2.948	2.765	0.707	2.121	6.205	300488
	Elastix B	3.122	3.178	0.707	2.121	7.000	311002
JFRC2013	ANTs A	2.697	2.771	0.707	2.000	5.568	321788
	ANTs B	3.434	3.348	0.707	2.345	7.681	299558
	ANTs C	3.219	3.168	0.707	2.236	7.036	303386
	CMTK A	3.011	2.874	0.707	2.121	6.403	293092
	CMTK B	3.169	2.972	0.707	2.236	6.819	290932
	CMTK C	4.035	3.561	0.707	3.000	8.485	263762
	Elastix A	2.726	2.688	0.707	2.000	5.701	320686
	Elastix B	2.674	2.733	0.707	2.000	5.568	328936
JRC2018	ANTs A	2.673	2.700	0.707	2.000	5.568	319308
	ANTs B	2.861	2.664	0.707	2.121	5.745	326204
	ANTs C	2.902	2.819	0.707	2.121	6.000	329458
	CMTK A	2.679	2.803	0.707	2.000	5.568	298028
	CMTK B	2.660	2.771	0.707	1.732	5.568	296780
	CMTK C	2.717	2.683	0.707	2.000	5.568	303460
	Elastix A	2.742	2.706	0.707	2.121	5.657	312578
	Elastix B	2.711	2.714	0.707	2.000	5.568	310436
FCWB	ANTs A	2.758	2.812	0.707	2.000	5.874	334680
	ANTs B	3.551	3.415	0.707	2.449	7.937	310126
	ANTs C	3.377	3.280	0.707	2.345	7.416	305450
	CMTK A	5.384	5.552	0.707	3.536	12.981	283240
	CMTK B	4.969	5.255	0.707	3.240	11.269	264802
	CMTK C	3.833	4.041	0.707	2.550	8.631	304944
	Elastix A	6.306	6.023	0.707	4.183	15.313	290364
	Elastix B	3.448	4.041	0.707	2.121	7.906	304660
Tefor	ANTs A	2.671	2.668	0.707	2.000	5.568	321736
	ANTs B	3.090	2.853	0.707	2.236	6.557	322866
	ANTs C	3.239	3.132	0.707	2.236	7.036	319676
	CMTK A	3.468	4.315	0.707	2.121	8.031	296402
	CMTK B	3.586	4.498	0.707	2.121	8.337	299568
	CMTK C	3.490	3.250	0.707	2.550	7.583	319366
	Elastix A	2.837	2.731	0.707	2.121	5.916	305932
	Elastix B	3.276	3.410	0.707	2.121	7.314	315504

Table S4. ICL_R : inferior clamp

Template	Algorithm	Mean	Std dev	10th perc	median	90th perc	N
JFRC2010	ANTs A	8.273	6.716	1.732	6.205	18.125	124266
	ANTs B	4.619	3.635	1.225	3.808	8.972	171134
	ANTs C	4.846	4.001	1.000	3.808	9.925	176908
	CMTK A	7.379	6.750	1.414	5.000	17.277	162750
	CMTK B	7.217	6.801	1.414	4.743	17.132	139584
	CMTK C	6.430	6.078	1.414	4.301	15.199	184334
	Elastix A	4.157	3.351	1.000	3.240	8.276	136550
	Elastix B	4.352	3.374	1.000	3.536	8.660	122002
JFRC2013	ANTs A	4.537	3.631	1.000	3.606	9.055	113054
	ANTs B	4.515	3.459	1.225	3.606	8.860	279266
	ANTs C	4.575	3.668	1.000	3.606	9.247	244618
	CMTK A	4.719	3.648	1.225	3.808	9.539	98662
	CMTK B	4.759	3.657	1.225	3.808	9.513	99628
	CMTK C	4.877	3.859	1.225	3.808	10.025	182464
	Elastix A	4.506	3.545	1.225	3.606	8.972	116176
	Elastix B	4.487	3.474	1.000	3.606	8.746	111036
JRC2018	ANTs A	4.464	3.631	1.000	3.606	8.972	111894
	ANTs B	4.932	3.717	1.225	4.062	9.823	136380
	ANTs C	4.973	3.597	1.414	4.062	9.925	135970
	CMTK A	4.531	3.617	1.000	3.606	9.000	95754
	CMTK B	4.536	3.632	1.000	3.606	9.055	92400
	CMTK C	4.594	3.672	1.000	3.674	9.220	100094
	Elastix A	4.487	3.531	1.000	3.606	8.718	118292
	Elastix B	4.466	3.374	1.000	3.674	8.660	112558
FCWB	ANTs A	4.787	3.985	1.000	3.674	9.592	112756
	ANTs B	4.878	3.636	1.414	4.062	9.513	221906
	ANTs C	4.971	3.861	1.414	4.062	9.849	184380
	CMTK A	7.352	5.921	1.581	5.431	16.340	189818
	CMTK B	6.945	5.472	1.581	5.292	14.868	139980
	CMTK C	6.271	4.814	1.581	4.950	13.285	153026
	Elastix A	7.003	5.628	1.581	5.431	14.474	264300
	Elastix B	7.055	5.435	1.581	5.431	15.330	205946
Tefor	ANTs A	4.442	3.502	1.000	3.606	8.746	120918
	ANTs B	4.467	3.393	1.225	3.606	8.660	201810
	ANTs C	5.259	3.794	1.414	4.359	10.271	194238
	CMTK A	7.314	5.607	1.581	5.788	16.016	156436
	CMTK B	7.404	5.644	1.581	5.874	16.016	160204
	CMTK C	6.877	5.227	1.581	5.292	14.849	165862
	Elastix A	4.217	3.378	1.000	3.464	8.185	147790
	Elastix B	4.809	3.647	1.225	3.808	9.925	168850

Table S5. VES_R : vest

Template	Algorithm	Mean	Std dev	10th perc	median	90th perc	N
JFRC2010	ANTs A	5.841	5.356	1.000	3.873	13.416	212072
	ANTs B	4.067	3.357	1.000	3.082	8.660	255490
	ANTs C	4.098	3.194	1.000	3.240	8.426	364138
	CMTK A	4.722	4.537	1.000	3.240	10.630	247624
	CMTK B	4.457	4.372	1.000	3.082	9.644	257816
	CMTK C	4.417	4.448	1.000	3.082	9.220	370982
	Elastix A	3.060	2.493	0.707	2.449	5.916	259080
	Elastix B	3.670	3.090	0.707	2.828	7.906	211838
JFRC2013	ANTs A	3.163	2.767	0.707	2.345	6.557	210128
	ANTs B	4.260	3.368	1.000	3.317	8.775	324356
	ANTs C	4.210	3.293	1.000	3.317	8.775	325204
	CMTK A	3.366	2.867	0.707	2.550	6.964	181380
	CMTK B	3.509	2.882	0.707	2.646	7.280	178796
	CMTK C	4.819	3.925	1.000	3.606	11.068	207494
	Elastix A	3.105	2.621	0.707	2.345	6.325	213226
	Elastix B	3.115	2.625	0.707	2.345	6.403	201400
JRC2018	ANTs A	3.101	2.674	0.707	2.345	6.403	216404
	ANTs B	3.405	2.886	0.707	2.646	7.071	226268
	ANTs C	3.214	2.667	0.707	2.550	6.557	227828
	CMTK A	3.321	3.016	0.707	2.449	7.106	183046
	CMTK B	3.190	2.844	0.707	2.345	6.671	181056
	CMTK C	3.128	2.739	0.707	2.345	6.442	195562
	Elastix A	3.155	2.681	0.707	2.449	6.403	203250
	Elastix B	3.184	2.710	0.707	2.449	6.633	198934
FCWB	ANTs A	3.343	2.863	0.707	2.550	6.856	208368
	ANTs B	4.804	3.684	1.000	3.808	10.149	295074
	ANTs C	3.921	2.902	1.000	3.162	7.906	345268
	CMTK A	6.347	5.291	1.225	4.743	13.964	204434
	CMTK B	5.750	4.965	1.000	4.123	12.923	198092
	CMTK C	4.281	3.728	1.000	3.082	9.513	246048
	Elastix A	7.917	7.926	1.414	5.292	18.152	242588
	Elastix B	4.365	4.009	1.000	3.162	9.434	222796
Tefor	ANTs A	3.107	2.665	0.707	2.345	6.364	212832
	ANTs B	3.848	3.211	1.000	2.915	8.031	239752
	ANTs C	3.998	3.250	1.000	3.082	8.515	275152
	CMTK A	4.508	4.170	0.707	3.000	10.863	206740
	CMTK B	4.501	4.218	0.707	3.000	10.977	208652
	CMTK C	4.508	3.874	1.000	3.240	10.223	206512
	Elastix A	3.207	2.703	0.707	2.550	6.481	219002
	Elastix B	3.689	3.088	0.707	2.828	8.000	211786

Table S6. IB_R : inferior bridge

Template	Algorithm	Mean	Std dev	10th perc	median	90th perc	N
JFRC2010	ANTs A	4.343	3.451	1.000	3.536	8.485	125792
	ANTs B	4.230	2.710	1.225	3.674	8.031	99154
	ANTs C	4.473	3.157	1.000	3.674	9.000	116250
	CMTK A	4.913	3.653	1.000	4.000	10.000	100040
	CMTK B	4.735	3.419	1.000	3.873	9.539	100534
	CMTK C	4.179	3.048	1.000	3.536	8.276	124706
	Elastix A	3.471	2.269	1.000	3.000	6.557	98164
	Elastix B	3.774	2.437	1.000	3.240	7.141	108068
JFRC2013	ANTs A	3.491	2.498	1.000	2.915	6.557	102024
	ANTs B	4.523	3.082	1.225	3.808	8.888	113776
	ANTs C	4.536	3.198	1.000	3.808	9.110	110298
	CMTK A	3.449	2.294	1.000	2.915	6.481	93474
	CMTK B	3.571	2.345	1.000	3.082	6.671	90208
	CMTK C	4.575	2.978	1.414	3.873	8.718	90024
	Elastix A	3.454	2.241	1.000	3.000	6.403	100400
	Elastix B	3.377	2.305	1.000	2.915	6.519	104878
JRC2018	ANTs A	3.239	2.275	1.000	2.646	6.083	105940
	ANTs B	3.613	2.450	1.000	3.082	6.708	101916
	ANTs C	3.546	2.438	1.000	3.000	6.671	101848
	CMTK A	3.383	2.437	1.000	2.915	6.481	100240
	CMTK B	3.374	2.375	1.000	2.915	6.442	99604
	CMTK C	3.408	2.293	1.000	2.915	6.364	97688
	Elastix A	3.435	2.284	1.000	3.000	6.519	96144
	Elastix B	3.345	2.313	1.000	2.915	6.442	105946
FCWB	ANTs A	3.665	2.486	1.000	3.162	6.856	95158
	ANTs B	4.521	2.948	1.225	3.808	8.746	107846
	ANTs C	4.260	2.853	1.225	3.606	8.367	118618
	CMTK A	5.780	5.223	1.414	4.359	11.358	72358
	CMTK B	5.523	5.176	1.414	4.062	11.180	76574
	CMTK C	4.258	2.995	1.225	3.606	8.093	88152
	Elastix A	6.748	6.980	1.581	4.950	12.207	91484
	Elastix B	4.385	3.467	1.000	3.536	8.660	93730
Tefor	ANTs A	3.239	2.249	1.000	2.828	6.083	104392
	ANTs B	4.069	2.637	1.225	3.606	7.616	99864
	ANTs C	4.376	3.001	1.225	3.674	8.544	108260
	CMTK A	3.885	2.724	1.000	3.240	7.583	112418
	CMTK B	3.824	2.622	1.000	3.240	7.348	114492
	CMTK C	4.280	2.708	1.225	3.808	8.000	98510
	Elastix A	3.430	2.297	1.000	2.915	6.633	104642
	Elastix B	3.480	2.372	1.000	3.000	6.671	117174

Table S7. ATL_R : antler

Template	Algorithm	Mean	Std dev	10th perc	median	90th perc	N
JFRC2010	ANTs A	8.622	6.716	1.581	7.036	17.847	256308
	ANTs B	4.956	3.951	1.225	3.808	10.075	146800
	ANTs C	5.196	3.865	1.414	4.243	10.223	146628
	CMTK A	8.446	8.462	1.581	5.788	17.958	118112
	CMTK B	7.776	7.473	1.581	5.431	16.654	119994
	CMTK C	7.686	7.710	1.732	5.523	15.133	137414
	Elastix A	5.452	4.060	1.414	4.528	10.794	141868
	Elastix B	7.431	5.954	1.581	5.745	15.764	122728
JFRC2013	ANTs A	4.568	3.730	1.000	3.606	9.247	138368
	ANTs B	6.720	4.943	1.581	5.523	13.675	128144
	ANTs C	6.563	5.076	1.581	5.196	13.528	133550
	CMTK A	5.413	4.345	1.225	4.183	11.180	130310
	CMTK B	5.513	4.335	1.225	4.359	11.380	135338
	CMTK C	6.112	4.696	1.414	5.000	12.490	141378
	Elastix A	5.079	3.812	1.414	4.123	9.950	135046
	Elastix B	5.653	4.511	1.414	4.472	11.533	132788
JRC2018	ANTs A	4.659	3.824	1.000	3.606	9.513	140804
	ANTs B	4.925	3.849	1.225	3.873	9.721	135624
	ANTs C	5.311	4.045	1.414	4.301	10.464	141770
	CMTK A	5.000	4.021	1.225	3.873	10.025	124912
	CMTK B	4.913	3.969	1.225	3.808	9.849	124664
	CMTK C	4.834	3.885	1.225	3.808	9.667	128432
	Elastix A	4.972	3.857	1.225	4.062	9.721	130008
	Elastix B	5.796	4.684	1.414	4.528	12.062	119008
FCWB	ANTs A	5.500	4.759	1.000	4.000	12.124	128116
	ANTs B	8.149	5.519	2.121	7.036	16.093	103628
	ANTs C	7.903	5.370	2.000	6.819	15.330	113236
	CMTK A	7.257	5.367	1.581	5.874	15.182	105712
	CMTK B	6.848	5.312	1.581	5.339	14.916	109638
	CMTK C	6.666	4.872	1.581	5.431	13.656	125260
	Elastix A	8.217	5.590	2.000	7.106	16.340	122506
	Elastix B	9.228	6.644	2.236	7.649	18.722	118268
Tefor	ANTs A	4.641	3.842	1.000	3.606	9.539	141758
	ANTs B	4.824	3.725	1.225	3.808	9.747	143886
	ANTs C	5.033	3.597	1.414	4.183	9.849	165882
	CMTK A	7.425	5.160	1.581	6.364	14.933	140012
	CMTK B	6.941	4.928	1.581	5.874	13.946	136286
	CMTK C	5.212	4.221	1.000	4.000	11.091	205034
	Elastix A	5.800	4.444	1.414	4.583	12.104	126216
	Elastix B	8.726	6.750	1.732	7.000	18.152	101056

Table S8. CRE_R : crepine

Template	Algorithm	Mean	Std dev	10th perc	median	90th perc	N
JFRC2010	ANTs A	7.214	7.129	1.581	5.196	14.177	74380
	ANTs B	5.165	3.881	1.225	4.123	10.630	70674
	ANTs C	5.380	4.167	1.225	4.183	11.489	68636
	CMTK A	5.749	4.344	1.000	4.528	12.186	64380
	CMTK B	6.011	5.345	1.000	4.301	13.509	61530
	CMTK C	6.030	5.006	1.414	4.528	12.669	59820
	Elastix A	5.279	3.905	1.414	4.183	10.977	64292
	Elastix B	5.713	4.552	1.414	4.359	12.367	66282
JFRC2013	ANTs A	4.841	3.786	1.000	3.808	10.271	73940
	ANTs B	5.863	4.432	1.414	4.690	12.000	63096
	ANTs C	5.789	4.415	1.414	4.583	11.937	59998
	CMTK A	5.166	4.027	1.225	4.123	10.724	72824
	CMTK B	5.404	4.078	1.225	4.359	11.180	70880
	CMTK C	5.706	4.186	1.414	4.583	11.937	63138
	Elastix A	4.885	3.756	1.225	3.808	10.247	70494
	Elastix B	4.837	3.748	1.000	3.808	10.149	69148
JRC2018	ANTs A	4.852	3.796	1.000	3.808	10.400	75608
	ANTs B	4.791	3.770	1.000	3.674	10.296	76030
	ANTs C	4.962	3.862	1.225	3.808	10.536	73184
	CMTK A	4.825	3.827	1.000	3.674	10.464	74458
	CMTK B	4.767	3.787	1.000	3.606	10.344	74242
	CMTK C	4.802	3.813	1.000	3.606	10.392	74460
	Elastix A	4.803	3.679	1.000	3.808	10.124	74648
	Elastix B	4.874	3.765	1.000	3.808	10.344	70694
FCWB	ANTs A	4.935	4.184	1.000	3.808	10.440	74328
	ANTs B	4.656	3.935	1.000	3.536	10.149	60650
	ANTs C	5.295	4.425	1.225	4.062	11.203	61662
	CMTK A	5.696	4.149	1.581	4.528	11.619	48344
	CMTK B	5.692	4.266	1.581	4.528	11.726	49326
	CMTK C	4.718	3.796	1.000	3.606	10.392	63940
	Elastix A	5.827	4.159	1.414	4.950	11.747	64586
	Elastix B	5.037	4.151	1.000	3.606	11.402	60602
Tefor	ANTs A	4.904	3.799	1.000	3.808	10.464	76484
	ANTs B	4.951	3.772	1.225	3.873	10.512	70062
	ANTs C	5.143	3.862	1.225	4.123	10.794	74764
	CMTK A	5.835	5.576	1.000	4.000	13.304	69062
	CMTK B	5.863	5.852	1.000	3.808	13.678	66370
	CMTK C	5.104	4.305	1.000	3.808	11.023	71454
	Elastix A	5.061	3.945	1.225	3.873	10.817	68372
	Elastix B	5.727	4.716	1.225	4.243	12.884	64534

Table S9. MB_PED_R : pedunculus of adult mushroom body

Template	Algorithm	Mean	Std dev	10th perc	median	90th perc	N
JFRC2010	ANTs A	6.250	5.399	1.414	4.583	13.865	148586
	ANTs B	5.022	3.986	1.414	4.062	9.513	104580
	ANTs C	5.003	3.820	1.414	4.123	9.618	120192
	CMTK A	5.027	4.511	1.000	3.674	11.000	118312
	CMTK B	6.132	7.278	1.000	3.808	14.248	114094
	CMTK C	6.397	6.223	1.581	4.359	14.663	93648
	Elastix A	4.394	3.757	1.000	3.536	8.660	121992
	Elastix B	4.544	3.910	1.225	3.606	8.276	134566
JFRC2013	ANTs A	5.091	3.742	1.414	4.301	9.721	116878
	ANTs B	5.689	4.373	1.581	4.528	11.180	100764
	ANTs C	5.703	4.106	1.581	4.743	11.158	97684
	CMTK A	4.824	3.670	1.225	4.062	9.327	118996
	CMTK B	5.142	3.702	1.581	4.359	9.721	112736
	CMTK C	6.148	4.094	1.732	5.196	11.576	71658
	Elastix A	4.220	3.618	1.000	3.240	8.185	128582
	Elastix B	4.297	3.732	1.000	3.317	8.093	133324
JRC2018	ANTs A	4.199	3.521	1.000	3.317	7.810	128470
	ANTs B	4.913	3.808	1.414	3.873	9.644	116984
	ANTs C	4.865	3.836	1.225	3.873	9.539	124096
	CMTK A	4.170	3.537	1.000	3.240	7.906	125576
	CMTK B	4.288	3.640	1.000	3.464	8.307	121318
	CMTK C	4.467	3.658	1.000	3.606	8.718	120790
	Elastix A	4.216	3.690	1.000	3.240	8.093	120206
	Elastix B	4.263	3.622	1.000	3.464	7.937	128328
FCWB	ANTs A	5.574	4.400	1.414	4.472	11.203	94886
	ANTs B	5.305	3.943	1.581	4.359	9.849	71238
	ANTs C	6.004	4.187	1.732	5.000	11.683	72886
	CMTK A	4.882	3.616	1.414	4.062	9.460	103216
	CMTK B	4.958	3.757	1.581	4.000	9.747	95366
	CMTK C	4.734	3.311	1.414	4.062	8.860	72578
	Elastix A	4.724	3.838	1.225	3.808	9.028	96340
	Elastix B	4.311	3.889	1.000	3.464	7.778	116388
Tefor	ANTs A	4.196	3.536	1.000	3.464	7.906	128050
	ANTs B	4.670	3.621	1.414	3.808	8.746	110182
	ANTs C	4.626	3.545	1.225	3.808	8.775	123050
	CMTK A	6.937	8.519	1.000	3.808	18.655	111368
	CMTK B	7.278	9.252	1.225	3.873	19.028	106430
	CMTK C	5.438	4.413	1.581	4.301	10.700	96924
	Elastix A	4.248	3.632	1.000	3.464	8.031	118540
	Elastix B	4.553	4.048	1.225	3.606	8.367	126070

Table S10. MB_VL_R : vertical lobe of adult mushroom body

Template	Algorithm	Mean	Std dev	10th perc	median	90th perc	N
JFRC2010	ANTs A	6.145	5.543	1.000	4.062	14.933	234816
	ANTs B	3.859	3.646	0.707	2.915	8.031	274290
	ANTs C	4.286	3.780	1.000	3.240	8.660	243720
	CMTK A	6.737	8.623	1.000	3.674	17.015	217274
	CMTK B	5.740	6.691	0.707	3.536	13.435	227764
	CMTK C	5.712	7.195	0.707	3.464	13.509	273894
	Elastix A	3.832	3.735	0.707	2.915	7.810	285614
	Elastix B	5.354	5.249	1.000	3.606	12.430	217368
JFRC2013	ANTs A	4.009	3.817	1.000	2.915	8.093	255556
	ANTs B	4.958	4.391	1.000	3.674	10.440	202548
	ANTs C	5.385	4.809	1.225	3.873	11.597	176168
	CMTK A	4.716	4.169	1.000	3.536	9.925	221616
	CMTK B	4.728	4.190	1.000	3.536	10.025	214090
	CMTK C	5.401	4.579	1.225	4.062	11.358	150036
	Elastix A	4.182	3.908	1.000	3.082	8.367	251898
	Elastix B	4.602	4.264	1.000	3.464	9.327	227968
JRC2018	ANTs A	4.031	3.839	1.000	2.915	8.031	258948
	ANTs B	3.986	3.757	0.707	2.915	8.093	257518
	ANTs C	4.227	3.829	1.000	3.162	8.396	249260
	CMTK A	4.172	3.922	1.000	3.082	8.396	249050
	CMTK B	4.162	3.868	1.000	3.082	8.396	244526
	CMTK C	4.092	3.771	1.000	3.000	8.307	245308
	Elastix A	4.164	3.812	1.000	3.162	8.276	252466
	Elastix B	4.867	4.504	1.000	3.606	10.025	230630
FCWB	ANTs A	4.441	4.299	1.000	3.162	9.301	220032
	ANTs B	5.193	4.423	1.225	4.062	10.464	142946
	ANTs C	5.800	4.831	1.414	4.528	12.166	131222
	CMTK A	5.238	4.257	1.225	4.062	10.724	211104
	CMTK B	4.978	4.098	1.225	3.808	10.223	219434
	CMTK C	4.454	3.875	1.000	3.317	9.220	196264
	Elastix A	5.595	4.525	1.225	4.359	11.533	209372
	Elastix B	6.610	6.433	1.000	4.243	16.583	187980
Tefor	ANTs A	4.081	3.838	1.000	3.000	8.185	254684
	ANTs B	3.871	3.607	0.707	2.915	7.842	267642
	ANTs C	4.381	3.767	1.000	3.240	8.775	255450
	CMTK A	4.782	3.948	1.000	3.674	9.721	245312
	CMTK B	4.813	4.047	1.000	3.674	9.899	239164
	CMTK C	4.671	4.179	1.000	3.536	9.925	279808
	Elastix A	4.214	3.832	0.707	3.162	8.515	251118
	Elastix B	7.834	7.745	1.414	4.950	20.267	149546

Table S11. MB_ML_R : medial lobe of adult mushroom body

Template	Algorithm	Mean	Std dev	10th perc	median	90th perc	N
JFRC2010	ANTs A	5.474	8.066	0.707	2.915	11.597	354210
	ANTs B	3.321	2.687	0.707	2.828	6.364	377092
	ANTs C	3.267	2.787	0.707	2.646	6.442	419414
	CMTK A	3.680	4.159	0.707	2.550	7.382	352410
	CMTK B	3.637	4.142	0.707	2.550	7.280	361674
	CMTK C	3.641	3.605	0.707	2.646	7.416	348282
	Elastix A	2.724	2.391	0.707	2.236	5.339	368494
	Elastix B	2.707	2.404	0.707	2.236	5.292	365130
JFRC2013	ANTs A	2.676	2.259	0.707	2.121	5.339	399670
	ANTs B	4.493	3.953	1.000	3.606	8.631	262524
	ANTs C	4.066	3.673	1.000	3.240	7.681	272910
	CMTK A	2.641	2.233	0.707	2.121	5.196	424002
	CMTK B	2.628	2.202	0.707	2.121	5.196	410600
	CMTK C	3.378	2.734	0.707	2.915	6.403	303820
	Elastix A	2.547	2.201	0.707	2.121	5.000	364092
	Elastix B	2.534	2.236	0.707	2.121	5.000	396174
JRC2018	ANTs A	2.420	2.106	0.707	2.000	4.743	408766
	ANTs B	3.128	2.713	0.707	2.550	6.205	384048
	ANTs C	3.450	3.071	0.707	2.646	6.819	379300
	CMTK A	2.459	2.190	0.707	2.121	4.899	449412
	CMTK B	2.465	2.233	0.707	2.121	4.796	421730
	CMTK C	2.471	2.173	0.707	2.121	4.950	402876
	Elastix A	2.551	2.158	0.707	2.121	5.000	398878
	Elastix B	2.616	2.482	0.707	2.121	5.148	389906
FCWB	ANTs A	2.524	2.465	0.707	2.121	5.000	422600
	ANTs B	3.848	3.358	1.000	3.082	7.246	303022
	ANTs C	3.835	3.391	1.000	3.000	7.348	316062
	CMTK A	3.217	2.742	0.707	2.646	6.325	393608
	CMTK B	3.221	2.928	0.707	2.646	6.124	391004
	CMTK C	3.130	2.665	0.707	2.550	6.124	336788
	Elastix A	4.111	4.140	0.707	3.240	7.937	305028
	Elastix B	3.161	2.643	0.707	2.550	6.364	371708
Tefor	ANTs A	2.495	2.341	0.707	2.121	5.000	426156
	ANTs B	3.402	2.944	0.707	2.828	6.557	366804
	ANTs C	3.480	3.206	0.707	2.646	6.745	414964
	CMTK A	3.079	2.677	0.707	2.449	6.364	379488
	CMTK B	3.319	3.008	0.707	2.550	6.964	363494
	CMTK C	3.703	3.878	0.707	2.646	6.964	363462
	Elastix A	2.881	2.397	0.707	2.345	5.657	369246
	Elastix B	3.062	2.759	0.707	2.550	6.000	344700

Table S12. FLA_R : flange

Template	Algorithm	Mean	Std dev	10th perc	median	90th perc	N
JFRC2010	ANTs A	8.661	6.730	2.345	6.928	17.132	475654
	ANTs B	6.802	4.843	2.121	5.745	12.767	388000
	ANTs C	7.298	4.926	2.121	6.325	13.583	458102
	CMTK A	8.485	5.918	2.236	7.106	16.628	509662
	CMTK B	7.640	5.088	2.121	6.519	14.832	460728
	CMTK C	7.212	4.914	2.121	6.083	13.946	497312
	Elastix A	5.645	4.406	1.581	4.528	10.863	368684
	Elastix B	5.415	4.242	1.581	4.472	10.296	403768
JFRC2013	ANTs A	6.053	5.289	1.581	4.743	11.937	442126
	ANTs B	6.657	5.179	1.732	5.385	13.000	445352
	ANTs C	7.051	5.202	2.000	5.874	13.528	444334
	CMTK A	7.234	6.544	1.581	5.196	15.182	426364
	CMTK B	7.264	6.489	1.732	5.339	14.866	424412
	CMTK C	6.756	4.706	2.121	5.701	12.767	449680
	Elastix A	5.801	4.865	1.581	4.528	11.424	450286
	Elastix B	5.711	4.972	1.414	4.472	11.336	482592
JRC2018	ANTs A	5.652	4.929	1.581	4.359	11.068	439706
	ANTs B	5.683	4.802	1.581	4.528	11.000	435238
	ANTs C	6.006	4.981	1.581	4.743	11.832	457370
	CMTK A	5.610	4.934	1.414	4.359	11.045	468788
	CMTK B	5.604	4.911	1.581	4.359	10.909	458608
	CMTK C	5.760	5.062	1.581	4.528	11.068	448426
	Elastix A	5.604	4.814	1.581	4.359	10.886	437394
	Elastix B	5.545	5.104	1.581	4.359	10.724	469178
FCWB	ANTs A	6.767	5.505	1.732	5.431	13.153	411806
	ANTs B	6.731	4.922	2.000	5.568	12.748	359238
	ANTs C	6.791	4.888	2.121	5.701	12.767	392490
	CMTK A	7.542	5.486	2.121	6.245	14.387	416848
	CMTK B	7.397	5.463	2.121	6.083	14.160	391324
	CMTK C	7.686	5.412	2.236	6.364	14.866	404082
	Elastix A	8.417	7.709	2.121	6.671	15.827	426276
	Elastix B	7.598	9.293	1.732	5.568	13.528	391602
Tefor	ANTs A	5.807	5.075	1.581	4.528	11.358	436920
	ANTs B	6.259	4.860	1.732	5.148	11.790	378356
	ANTs C	6.948	5.085	2.000	5.831	13.304	416008
	CMTK A	7.139	5.660	1.732	5.701	14.230	471104
	CMTK B	7.117	5.625	1.732	5.701	13.946	455220
	CMTK C	6.553	5.365	1.732	5.196	12.430	409754
	Elastix A	5.593	4.687	1.581	4.416	10.886	376232
	Elastix B	5.431	4.587	1.581	4.301	10.630	384092

Table S13. LOP_R : lobula plate

Template	Algorithm	Mean	Std dev	10th perc	median	90th perc	N
JFRC2010	ANTs A	5.630	5.639	1.414	3.808	13.675	239966
	ANTs B	3.587	2.022	1.225	3.240	6.245	267694
	ANTs C	3.702	2.098	1.225	3.464	6.481	259974
	CMTK A	6.327	7.018	1.581	4.359	11.336	246854
	CMTK B	5.277	4.698	1.581	4.062	9.849	244064
	CMTK C	5.338	4.636	1.581	4.183	9.721	246128
	Elastix A	3.402	1.932	1.000	3.082	6.000	293972
	Elastix B	4.024	2.695	1.225	3.536	7.517	293234
JFRC2013	ANTs A	3.489	1.992	1.225	3.240	6.083	269494
	ANTs B	3.909	2.298	1.414	3.606	6.964	287216
	ANTs C	3.825	2.245	1.225	3.536	6.819	279940
	CMTK A	3.627	2.071	1.225	3.317	6.364	263754
	CMTK B	3.718	2.138	1.225	3.464	6.557	271152
	CMTK C	3.374	2.116	1.000	2.915	6.205	355066
	Elastix A	3.543	1.984	1.225	3.240	6.124	281728
	Elastix B	3.470	1.948	1.225	3.240	6.083	293524
JRC2018	ANTs A	3.477	1.982	1.225	3.162	6.083	269700
	ANTs B	3.547	1.995	1.225	3.240	6.164	266400
	ANTs C	3.597	2.017	1.225	3.317	6.245	266182
	CMTK A	3.601	2.110	1.225	3.240	6.403	242150
	CMTK B	3.603	2.092	1.225	3.240	6.364	243970
	CMTK C	3.583	2.018	1.225	3.240	6.205	256428
	Elastix A	3.558	1.989	1.225	3.240	6.124	265772
	Elastix B	3.570	2.055	1.225	3.240	6.325	276532
FCWB	ANTs A	3.468	1.976	1.225	3.162	6.042	265968
	ANTs B	3.525	2.414	1.000	3.000	6.856	373646
	ANTs C	3.636	2.399	1.000	3.082	6.964	345156
	CMTK A	5.688	4.228	1.581	4.528	11.683	302648
	CMTK B	5.225	3.956	1.414	4.123	10.817	302658
	CMTK C	3.877	2.675	1.000	3.240	7.583	330860
	Elastix A	4.572	3.166	1.414	3.873	8.746	384774
	Elastix B	4.932	3.963	1.414	3.808	9.721	247436
Tefor	ANTs A	3.474	1.984	1.000	3.162	6.083	267228
	ANTs B	3.589	2.025	1.225	3.317	6.205	258562
	ANTs C	3.596	2.055	1.225	3.317	6.364	272178
	CMTK A	4.521	2.941	1.414	3.873	8.396	240954
	CMTK B	4.452	2.870	1.414	3.808	8.307	242188
	CMTK C	4.120	2.555	1.414	3.606	7.550	302688
	Elastix A	3.495	2.007	1.225	3.162	6.124	281684
	Elastix B	4.676	3.586	1.414	3.808	9.220	260680

Table S14. EB : ellipsoid body

Template	Algorithm	Mean	Std dev	10th perc	median	90th perc	N
JFRC2010	ANTs A	5.255	6.244	1.225	3.464	10.817	1405498
	ANTs B	3.445	2.357	1.000	3.000	6.000	1443546
	ANTs C	3.525	2.492	1.225	3.082	6.083	1542696
	CMTK A	4.328	4.241	1.225	3.240	7.649	1383128
	CMTK B	4.419	4.625	1.225	3.240	7.746	1366692
	CMTK C	4.983	5.726	1.225	3.464	9.110	1307010
	Elastix A	3.232	2.074	1.000	2.915	5.568	1456306
	Elastix B	3.285	2.093	1.000	3.000	5.701	1500946
JFRC2013	ANTs A	3.215	2.012	1.000	2.915	5.568	1517212
	ANTs B	4.297	3.658	1.225	3.317	8.246	1094568
	ANTs C	4.446	4.027	1.225	3.317	8.746	1157590
	CMTK A	3.505	2.526	1.000	3.000	6.083	1436332
	CMTK B	3.548	2.519	1.000	3.082	6.205	1403924
	CMTK C	3.778	2.778	1.225	3.162	6.964	1085438
	Elastix A	3.216	2.060	1.000	2.915	5.523	1497794
	Elastix B	3.194	2.043	1.000	2.915	5.523	1549406
JRC2018	ANTs A	3.144	1.980	1.000	2.915	5.431	1549380
	ANTs B	3.343	2.076	1.000	3.000	5.745	1520126
	ANTs C	3.473	2.145	1.225	3.162	6.083	1530696
	CMTK A	3.152	1.987	1.000	2.915	5.431	1545220
	CMTK B	3.165	1.989	1.000	2.915	5.431	1555946
	CMTK C	3.213	2.012	1.000	2.915	5.523	1561296
	Elastix A	3.220	2.019	1.000	2.915	5.523	1557730
	Elastix B	3.201	2.031	1.000	2.915	5.523	1547092
FCWB	ANTs A	3.468	2.336	1.000	3.082	6.042	1524310
	ANTs B	3.959	3.016	1.225	3.240	7.280	1045354
	ANTs C	4.367	3.836	1.225	3.464	8.246	1108210
	CMTK A	3.575	2.365	1.225	3.162	6.205	1536540
	CMTK B	3.578	2.458	1.225	3.162	6.205	1510018
	CMTK C	3.720	2.562	1.225	3.240	6.557	1328746
	Elastix A	3.644	2.546	1.225	3.240	6.364	1463492
	Elastix B	3.451	2.230	1.225	3.082	6.083	1487654
Tefor	ANTs A	3.175	2.013	1.000	2.915	5.523	1543712
	ANTs B	3.390	2.263	1.000	3.000	5.874	1526548
	ANTs C	3.696	3.147	1.225	3.162	6.205	1584950
	CMTK A	3.725	2.591	1.225	3.240	6.708	1540136
	CMTK B	3.703	2.566	1.225	3.240	6.671	1554432
	CMTK C	3.884	3.081	1.225	3.240	6.964	1531560
	Elastix A	3.291	2.106	1.000	3.000	5.701	1509564
	Elastix B	3.646	2.421	1.225	3.240	6.519	1498242

Table S15. AL_R : adult antennal lobe

Template	Algorithm	Mean	Std dev	10th perc	median	90th perc	N
JFRC2010	ANTs A	6.367	6.837	1.581	4.743	11.402	3492090
	ANTs B	5.040	4.515	1.581	4.243	8.860	2962300
	ANTs C	5.358	4.467	1.581	4.528	9.513	3300890
	CMTK A	5.502	4.495	1.581	4.637	10.050	3257940
	CMTK B	5.198	4.364	1.581	4.528	9.220	3184846
	CMTK C	5.766	4.471	1.732	5.000	10.271	3006892
	Elastix A	4.629	4.184	1.581	4.062	8.031	3067542
	Elastix B	4.741	4.283	1.581	4.123	8.246	3114026
JFRC2013	ANTs A	4.812	4.251	1.581	4.123	8.544	3210516
	ANTs B	5.407	4.875	1.581	4.528	9.721	3020702
	ANTs C	5.796	4.699	1.732	5.000	10.344	3127004
	CMTK A	5.781	5.388	1.581	4.690	10.025	2897718
	CMTK B	6.030	5.700	1.732	4.796	10.416	2829810
	CMTK C	6.238	5.048	2.121	5.385	10.630	2760140
	Elastix A	4.653	4.131	1.581	4.062	8.185	3280756
	Elastix B	4.628	4.071	1.414	4.062	8.124	3247838
JRC2018	ANTs A	4.601	4.073	1.414	4.000	8.093	3275738
	ANTs B	5.017	4.679	1.581	4.183	8.775	3215812
	ANTs C	5.493	5.040	1.581	4.528	9.899	3275980
	CMTK A	4.632	4.189	1.414	4.062	8.093	3215146
	CMTK B	4.670	4.144	1.581	4.062	8.155	3236520
	CMTK C	4.832	4.261	1.581	4.183	8.515	3271916
	Elastix A	4.615	4.321	1.581	4.062	7.937	3205366
	Elastix B	4.653	4.120	1.581	4.062	8.093	3201122
FCWB	ANTs A	4.795	4.176	1.581	4.123	8.426	3302824
	ANTs B	5.485	4.647	1.581	4.690	9.644	3156232
	ANTs C	6.412	5.015	2.000	5.523	11.446	3176428
	CMTK A	6.061	5.185	1.732	5.000	11.068	3333848
	CMTK B	5.863	5.094	1.732	4.796	10.583	3310852
	CMTK C	6.024	4.836	1.732	5.196	10.770	3069468
	Elastix A	6.627	6.804	2.000	5.385	12.490	3344776
	Elastix B	5.859	6.269	1.581	4.690	10.886	3331994
Tefor	ANTs A	4.633	4.197	1.414	4.000	8.185	3254842
	ANTs B	5.102	4.661	1.581	4.183	9.055	2995846
	ANTs C	5.812	4.841	1.581	4.950	10.392	3193460
	CMTK A	6.927	7.527	1.581	4.743	14.370	2953692
	CMTK B	6.898	7.427	1.581	4.743	14.177	2969518
	CMTK C	6.883	8.348	1.581	4.743	12.124	2878476
	Elastix A	4.589	4.112	1.581	4.000	8.031	3103026
	Elastix B	4.674	4.183	1.581	4.062	8.185	3084188

Table S16. ME_R : medulla

Template	Algorithm	Mean	Std dev	10th perc	median	90th perc	N
JFRC2010	ANTs A	5.048	5.468	1.000	3.000	12.410	1232930
	ANTs B	3.309	2.410	1.000	2.646	6.481	1139068
	ANTs C	3.804	2.625	1.000	3.162	7.416	978550
	CMTK A	5.801	6.424	1.000	3.606	13.077	980474
	CMTK B	5.218	5.666	1.000	3.240	11.597	1010442
	CMTK C	5.590	5.957	1.000	3.808	11.683	899172
	Elastix A	2.846	2.128	0.707	2.345	5.568	1484426
	Elastix B	4.096	3.661	1.000	2.915	9.055	1327662
JFRC2013	ANTs A	2.866	2.161	0.707	2.345	5.568	1182032
	ANTs B	3.689	2.667	1.000	3.000	7.314	1066842
	ANTs C	3.585	2.573	1.000	2.915	7.106	1061936
	CMTK A	3.059	2.334	0.707	2.550	6.042	1038332
	CMTK B	3.172	2.384	0.707	2.550	6.205	1104670
	CMTK C	3.088	2.402	0.707	2.550	6.205	1491892
	Elastix A	2.591	2.023	0.707	2.121	5.148	1532410
	Elastix B	3.130	2.347	0.707	2.550	6.245	1314074
JRC2018	ANTs A	2.852	2.162	0.707	2.236	5.568	1186334
	ANTs B	2.886	2.178	0.707	2.345	5.701	1202048
	ANTs C	3.011	2.261	0.707	2.449	5.874	1109464
	CMTK A	3.088	2.394	0.707	2.550	6.083	981638
	CMTK B	3.043	2.366	0.707	2.449	6.042	986594
	CMTK C	2.906	2.216	0.707	2.345	5.701	1130808
	Elastix A	2.790	2.084	0.707	2.236	5.431	1350790
	Elastix B	3.109	2.276	0.707	2.550	6.083	1221460
FCWB	ANTs A	2.883	2.187	0.707	2.345	5.701	1171342
	ANTs B	3.570	2.724	1.000	2.915	7.416	1313512
	ANTs C	3.664	2.666	1.000	3.000	7.348	1167140
	CMTK A	4.423	3.630	1.000	3.317	9.460	1075760
	CMTK B	4.064	3.449	1.000	3.000	8.718	1131062
	CMTK C	3.777	2.946	1.000	3.000	7.810	1222786
	Elastix A	4.905	4.068	1.000	3.808	9.950	1113916
	Elastix B	4.592	4.286	1.000	3.240	9.950	1104420
Tefor	ANTs A	2.855	2.166	0.707	2.236	5.568	1183568
	ANTs B	3.241	2.314	1.000	2.646	6.205	1104308
	ANTs C	3.460	2.504	1.000	2.915	6.671	1056266
	CMTK A	4.151	3.544	1.000	3.082	8.718	959660
	CMTK B	4.119	3.581	1.000	3.000	8.631	959546
	CMTK C	3.706	2.728	1.000	3.000	7.211	1116838
	Elastix A	3.119	2.260	0.707	2.550	6.124	1392394
	Elastix B	4.474	4.006	1.000	3.162	9.925	1202828

Table S17. FB : fan-shaped body

Template	Algorithm	Mean	Std dev	10th perc	median	90th perc	N
JFRC2010	ANTs A	6.076	5.901	1.000	4.359	13.058	554674
	ANTs B	4.294	4.281	0.707	2.915	9.849	527882
	ANTs C	4.400	4.364	0.707	3.000	9.874	607662
	CMTK A	4.388	5.115	0.707	2.449	10.700	533858
	CMTK B	4.230	4.929	0.707	2.449	10.025	538746
	CMTK C	4.618	5.462	0.707	2.646	11.000	488598
	Elastix A	3.670	4.166	0.707	2.236	8.544	479446
	Elastix B	4.066	4.622	0.707	2.345	9.644	486562
JFRC2013	ANTs A	3.919	4.223	0.707	2.345	9.192	572468
	ANTs B	4.502	4.416	0.707	3.000	10.392	544380
	ANTs C	4.397	4.204	0.707	3.000	9.925	600090
	CMTK A	4.714	4.469	0.707	3.317	10.320	488296
	CMTK B	4.836	4.415	1.000	3.606	10.392	496718
	CMTK C	5.572	4.328	1.225	4.528	11.446	476410
	Elastix A	3.777	4.257	0.707	2.236	8.746	520120
	Elastix B	3.781	4.266	0.707	2.236	8.746	521868
JRC2018	ANTs A	3.668	4.104	0.707	2.236	8.396	567250
	ANTs B	4.115	4.200	0.707	2.828	9.220	537770
	ANTs C	4.129	4.166	0.707	2.915	9.301	605432
	CMTK A	3.765	4.167	0.707	2.345	8.631	566692
	CMTK B	3.674	4.092	0.707	2.236	8.367	559170
	CMTK C	3.728	4.005	0.707	2.345	8.426	570396
	Elastix A	3.593	4.034	0.707	2.236	8.185	538056
	Elastix B	3.699	4.099	0.707	2.236	8.544	536820
FCWB	ANTs A	4.160	4.482	0.707	2.646	9.539	556460
	ANTs B	5.174	4.663	1.000	3.808	11.203	508568
	ANTs C	5.233	4.592	1.000	3.873	11.247	580398
	CMTK A	4.882	4.203	1.000	3.808	10.000	531436
	CMTK B	4.957	4.259	1.000	3.873	10.223	537216
	CMTK C	4.685	4.879	0.707	3.000	10.770	519088
	Elastix A	4.645	4.958	0.707	2.915	11.091	498018
	Elastix B	4.401	4.801	0.707	2.646	10.512	507776
Tefor	ANTs A	3.653	4.029	0.707	2.236	8.396	581644
	ANTs B	4.273	4.032	0.707	3.000	9.247	596168
	ANTs C	4.294	4.136	0.707	3.000	9.460	685590
	CMTK A	4.831	6.287	0.707	2.236	12.923	560746
	CMTK B	4.883	6.282	0.707	2.449	12.530	564910
	CMTK C	4.645	4.783	0.707	3.082	10.416	608104
	Elastix A	3.708	4.143	0.707	2.236	8.485	540494
	Elastix B	3.954	4.428	0.707	2.345	9.327	533868

Table S18. SLP_R : superior lateral protocerebrum

Template	Algorithm	Mean	Std dev	10th perc	median	90th perc	N
JFRC2010	ANTs A	7.575	5.727	1.581	6.245	15.199	204222
	ANTs B	5.244	4.159	1.225	4.183	10.416	102366
	ANTs C	5.392	4.289	1.225	4.301	10.886	116736
	CMTK A	5.940	5.114	1.225	4.528	12.767	105298
	CMTK B	5.471	4.547	1.225	4.243	10.909	101264
	CMTK C	6.197	5.491	1.414	4.528	13.528	98082
	Elastix A	5.209	4.207	1.225	4.183	10.271	104036
	Elastix B	5.570	4.131	1.225	4.690	10.977	122510
JFRC2013	ANTs A	4.967	3.983	1.000	3.873	9.950	108890
	ANTs B	5.609	4.816	1.225	4.243	11.747	93530
	ANTs C	5.407	4.535	1.225	4.123	11.380	102344
	CMTK A	5.623	4.337	1.225	4.583	11.269	118528
	CMTK B	5.757	4.399	1.414	4.690	11.769	117768
	CMTK C	5.766	4.395	1.414	4.637	11.790	104072
	Elastix A	5.003	4.098	1.000	3.873	10.025	113408
	Elastix B	5.004	3.964	1.000	4.062	9.874	114900
JRC2018	ANTs A	4.809	3.954	1.000	3.808	9.618	111998
	ANTs B	5.013	4.123	1.000	3.873	10.223	108210
	ANTs C	5.263	4.129	1.225	4.243	10.630	109810
	CMTK A	4.777	4.025	1.000	3.674	9.618	111720
	CMTK B	4.790	4.076	1.000	3.674	9.618	107786
	CMTK C	4.889	4.121	1.000	3.808	9.849	106268
	Elastix A	4.679	3.873	1.225	3.606	9.220	107928
	Elastix B	4.810	3.777	1.225	3.808	9.487	114296
FCWB	ANTs A	6.009	5.202	1.225	4.472	12.826	93454
	ANTs B	5.783	5.018	1.225	4.243	12.767	87372
	ANTs C	5.802	4.799	1.225	4.472	12.288	99798
	CMTK A	5.872	4.723	1.581	4.583	12.104	102856
	CMTK B	5.891	4.753	1.581	4.528	12.288	102774
	CMTK C	4.843	4.433	1.000	3.536	10.392	118616
	Elastix A	5.806	4.555	1.581	4.528	11.895	109416
	Elastix B	5.494	4.143	1.581	4.528	10.794	120090
Tefor	ANTs A	4.873	3.964	1.000	3.808	9.874	115056
	ANTs B	4.940	3.859	1.225	3.873	9.899	115340
	ANTs C	5.046	4.024	1.225	4.062	10.392	128406
	CMTK A	6.835	6.888	1.225	4.528	15.811	108018
	CMTK B	6.723	6.262	1.225	4.528	15.716	108284
	CMTK C	5.414	4.475	1.225	4.183	11.023	141996
	Elastix A	4.973	4.117	1.000	3.873	10.025	104854
	Elastix B	5.432	4.113	1.225	4.528	10.817	115676

Table S19. SIP_R : superior intermediate protocerebrum

Template	Algorithm	Mean	Std dev	10th perc	median	90th perc	N
JFRC2010	ANTs A	4.344	4.449	0.707	2.915	10.149	2145362
	ANTs B	2.678	2.441	0.707	2.121	5.701	2003074
	ANTs C	2.726	2.483	0.707	2.121	5.788	2169630
	CMTK A	4.002	5.297	0.707	2.345	8.544	1631608
	CMTK B	3.727	4.456	0.707	2.236	8.337	1653970
	CMTK C	3.889	5.259	0.707	2.236	8.396	1886180
	Elastix A	2.773	2.542	0.707	2.121	5.831	1719396
	Elastix B	3.650	3.492	0.707	2.550	8.093	1589316
JFRC2013	ANTs A	2.489	2.351	0.707	1.732	5.292	1971190
	ANTs B	2.799	2.583	0.707	2.121	5.916	1889764
	ANTs C	2.804	2.629	0.707	2.121	6.042	1873850
	CMTK A	2.739	2.587	0.707	2.000	5.874	1856670
	CMTK B	2.860	2.638	0.707	2.121	6.164	1832456
	CMTK C	3.399	2.853	0.707	2.646	7.141	1670644
	Elastix A	2.595	2.461	0.707	2.000	5.431	1868890
	Elastix B	2.817	2.723	0.707	2.121	6.000	1851748
JRC2018	ANTs A	2.531	2.454	0.707	1.732	5.431	1976502
	ANTs B	2.569	2.464	0.707	2.000	5.431	1908536
	ANTs C	2.583	2.501	0.707	1.732	5.523	1983574
	CMTK A	2.515	2.362	0.707	2.000	5.196	1963182
	CMTK B	2.547	2.379	0.707	2.000	5.196	1884060
	CMTK C	2.598	2.432	0.707	2.000	5.385	1856570
	Elastix A	2.639	2.456	0.707	2.000	5.523	1831450
	Elastix B	2.779	2.623	0.707	2.121	5.874	1873824
FCWB	ANTs A	2.694	2.593	0.707	2.000	5.745	1984280
	ANTs B	3.214	2.842	0.707	2.449	6.708	1554920
	ANTs C	3.343	3.080	0.707	2.550	7.071	1532000
	CMTK A	3.123	2.787	0.707	2.345	6.557	1858790
	CMTK B	3.052	2.743	0.707	2.236	6.442	1759028
	CMTK C	3.273	2.850	0.707	2.449	7.036	1688338
	Elastix A	3.892	3.776	0.707	2.646	8.631	1591698
	Elastix B	3.542	3.348	0.707	2.550	7.778	1661438
Tefor	ANTs A	2.519	2.438	0.707	1.732	5.385	1968522
	ANTs B	2.654	2.423	0.707	2.000	5.701	2000184
	ANTs C	2.682	2.415	0.707	2.121	5.701	2215018
	CMTK A	3.435	4.027	0.707	2.236	7.382	1750938
	CMTK B	3.379	3.966	0.707	2.236	7.106	1758512
	CMTK C	3.123	3.003	0.707	2.236	6.708	1928770
	Elastix A	2.797	2.604	0.707	2.121	5.874	1803676
	Elastix B	3.623	3.452	0.707	2.550	8.185	1746914

Table S20. SMP_R : superior medial protocerebrum

Template	Algorithm	Mean	Std dev	10th perc	median	90th perc	N
JFRC2010	ANTs A	5.328	4.559	1.581	4.359	9.539	21022
	ANTs B	4.721	2.711	1.581	4.472	8.307	22324
	ANTs C	4.854	2.793	1.581	4.528	8.544	21462
	CMTK A	4.916	2.653	1.581	4.583	8.602	19230
	CMTK B	4.778	2.701	1.581	4.472	8.515	19312
	CMTK C	5.485	3.217	1.732	5.000	9.670	19076
	Elastix A	4.435	2.446	1.581	4.123	7.906	20304
	Elastix B	4.399	2.359	1.581	4.123	7.681	18320
JFRC2013	ANTs A	3.966	2.251	1.225	3.606	7.106	22506
	ANTs B	4.513	2.463	1.581	4.183	7.906	20674
	ANTs C	4.457	2.584	1.414	4.183	8.031	22488
	CMTK A	4.903	4.014	1.581	3.873	8.746	23436
	CMTK B	4.725	3.632	1.581	4.000	8.185	23594
	CMTK C	4.572	3.361	1.414	4.062	7.810	22442
	Elastix A	3.940	2.246	1.225	3.606	7.106	22708
	Elastix B	4.360	2.360	1.581	4.062	7.681	19070
JRC2018	ANTs A	3.985	2.257	1.225	3.674	7.106	22296
	ANTs B	4.104	2.348	1.225	3.808	7.246	23672
	ANTs C	4.189	2.410	1.225	3.873	7.517	25636
	CMTK A	4.127	2.239	1.414	3.808	7.280	22846
	CMTK B	4.110	2.241	1.414	3.808	7.211	22464
	CMTK C	4.105	2.268	1.414	3.808	7.141	23220
	Elastix A	3.948	2.271	1.225	3.606	7.106	21308
	Elastix B	4.300	2.349	1.581	4.062	7.583	19508
FCWB	ANTs A	3.976	2.155	1.414	3.674	7.000	22106
	ANTs B	4.863	2.699	1.581	4.472	8.660	15848
	ANTs C	5.271	3.076	1.581	4.899	9.721	21440
	CMTK A	4.842	3.363	1.581	4.123	9.083	20416
	CMTK B	4.630	2.956	1.581	4.123	8.307	21260
	CMTK C	4.759	2.536	1.581	4.528	8.337	18488
	Elastix A	4.887	3.985	1.414	4.123	8.307	23268
	Elastix B	4.011	2.391	1.225	3.606	7.314	26318
Tefor	ANTs A	4.222	2.363	1.414	3.873	7.517	20588
	ANTs B	4.435	2.530	1.395	4.183	7.906	23460
	ANTs C	4.775	2.903	1.414	4.359	8.888	36240
	CMTK A	5.384	4.025	1.581	4.528	9.407	20008
	CMTK B	5.079	3.479	1.581	4.528	8.944	19784
	CMTK C	4.585	2.997	1.225	4.123	8.515	22322
	Elastix A	4.339	2.475	1.414	4.062	7.810	22868
	Elastix B	4.434	2.432	1.581	4.123	7.810	19622

Table S21. AME_R : accessory medulla

Template	Algorithm	Mean	Std dev	10th perc	median	90th perc	N
JFRC2010	ANTs A	7.648	6.824	1.732	5.568	16.263	636130
	ANTs B	5.618	3.997	1.581	4.743	10.886	609838
	ANTs C	5.737	4.157	1.581	4.743	11.247	675888
	CMTK A	6.115	4.523	1.581	5.050	12.104	690236
	CMTK B	6.010	4.515	1.581	4.950	12.000	669340
	CMTK C	6.487	5.247	1.581	5.196	12.767	577554
	Elastix A	5.060	3.534	1.414	4.359	9.539	665630
	Elastix B	5.627	4.177	1.414	4.637	11.045	714568
JFRC2013	ANTs A	4.934	3.495	1.414	4.183	9.301	682792
	ANTs B	6.238	4.374	1.581	5.196	12.124	610754
	ANTs C	6.265	4.588	1.581	5.196	12.510	631998
	CMTK A	5.675	4.003	1.581	4.743	10.840	639244
	CMTK B	5.795	4.097	1.581	4.950	11.136	616978
	CMTK C	6.599	4.397	2.000	5.701	12.430	491772
	Elastix A	4.974	3.459	1.414	4.301	9.301	691716
	Elastix B	4.997	3.527	1.414	4.243	9.460	709604
JRC2018	ANTs A	4.907	3.465	1.414	4.183	9.301	690704
	ANTs B	5.110	3.605	1.414	4.359	9.644	665526
	ANTs C	5.286	3.727	1.414	4.528	10.100	701290
	CMTK A	4.881	3.416	1.414	4.183	9.165	680930
	CMTK B	4.901	3.459	1.414	4.183	9.220	670036
	CMTK C	4.990	3.538	1.414	4.243	9.434	655926
	Elastix A	4.956	3.476	1.414	4.243	9.301	669066
	Elastix B	4.975	3.490	1.414	4.243	9.434	684326
FCWB	ANTs A	5.468	4.045	1.414	4.528	10.677	661724
	ANTs B	6.114	4.267	1.581	5.196	11.790	519096
	ANTs C	6.485	4.682	1.581	5.431	12.748	542078
	CMTK A	6.167	4.299	1.581	5.196	11.958	642618
	CMTK B	5.998	4.256	1.581	5.050	11.662	618072
	CMTK C	6.080	4.249	1.581	5.148	11.853	534082
	Elastix A	6.115	4.193	1.581	5.196	11.683	653536
	Elastix B	5.540	4.195	1.414	4.528	10.724	657850
Tefor	ANTs A	4.941	3.490	1.414	4.183	9.301	707608
	ANTs B	5.279	3.596	1.581	4.528	9.849	654438
	ANTs C	5.499	3.863	1.581	4.690	10.464	698276
	CMTK A	6.070	5.111	1.414	4.743	12.207	751188
	CMTK B	6.105	5.215	1.414	4.743	12.430	731016
	CMTK C	5.735	4.566	1.581	4.637	11.023	686572
	Elastix A	5.028	3.540	1.414	4.359	9.460	667658
	Elastix B	5.790	4.438	1.414	4.743	11.424	750524

Table S22. AVLP_R : anterior ventrolateral protocerebrum

Template	Algorithm	Mean	Std dev	10th perc	median	90th perc	N
JFRC2010	ANTs A	8.850	8.167	1.732	6.083	21.225	258570
	ANTs B	5.796	4.041	1.581	5.000	10.977	234778
	ANTs C	6.246	4.321	1.581	5.431	11.853	238116
	CMTK A	7.740	6.956	1.581	5.745	16.462	248546
	CMTK B	7.136	6.372	1.581	5.292	15.017	238766
	CMTK C	7.116	5.549	1.732	5.701	14.474	236602
	Elastix A	5.201	3.824	1.414	4.359	9.950	255894
	Elastix B	5.506	3.975	1.581	4.583	10.654	249718
JFRC2013	ANTs A	4.917	3.730	1.225	4.062	9.618	259998
	ANTs B	6.159	4.270	1.581	5.292	11.853	241008
	ANTs C	5.926	4.120	1.581	5.099	11.158	238628
	CMTK A	5.523	4.238	1.414	4.528	10.817	261984
	CMTK B	5.565	4.226	1.414	4.528	10.977	261318
	CMTK C	6.056	4.148	1.581	5.196	11.489	257662
	Elastix A	4.986	3.809	1.225	4.062	9.721	257162
	Elastix B	4.987	3.771	1.225	4.062	9.721	247356
JRC2018	ANTs A	4.949	3.758	1.225	4.062	9.644	261926
	ANTs B	5.112	3.790	1.225	4.243	9.925	260726
	ANTs C	5.137	3.807	1.225	4.301	9.950	255404
	CMTK A	4.996	3.717	1.225	4.123	9.721	249954
	CMTK B	5.002	3.749	1.225	4.123	9.823	250250
	CMTK C	5.020	3.780	1.225	4.123	9.747	252970
	Elastix A	4.997	3.771	1.225	4.123	9.644	260550
	Elastix B	5.043	3.778	1.225	4.123	9.874	251554
FCWB	ANTs A	5.107	3.833	1.225	4.183	10.050	258754
	ANTs B	6.447	4.355	1.732	5.568	12.186	225392
	ANTs C	6.809	4.569	1.732	5.874	12.767	222422
	CMTK A	6.412	4.594	1.581	5.431	12.227	241870
	CMTK B	6.228	4.468	1.581	5.196	12.124	247198
	CMTK C	6.431	4.558	1.581	5.431	12.510	223584
	Elastix A	7.233	5.142	1.732	6.164	13.748	272934
	Elastix B	5.672	4.057	1.581	4.743	11.158	254500
Tefor	ANTs A	4.970	3.767	1.225	4.062	9.644	265258
	ANTs B	5.573	3.957	1.581	4.743	10.630	258156
	ANTs C	5.740	4.029	1.581	4.950	10.909	255876
	CMTK A	7.151	6.753	1.414	5.000	16.371	257948
	CMTK B	7.290	7.204	1.414	4.950	17.000	253230
	CMTK C	6.315	5.193	1.581	4.950	13.058	264876
	Elastix A	5.191	3.862	1.414	4.301	9.950	258122
	Elastix B	5.595	4.156	1.414	4.583	10.977	250188

Table S23. PVLP_R : posterior ventrolateral protocerebrum

Template	Algorithm	Mean	Std dev	10th perc	median	90th perc	N
JFRC2010	ANTs A	6.466	5.088	1.732	5.000	13.675	201136
	ANTs B	5.086	3.309	1.732	4.528	8.718	175108
	ANTs C	5.113	3.279	1.732	4.528	8.944	186916
	CMTK A	5.684	4.142	1.732	4.743	10.607	180800
	CMTK B	5.385	3.625	1.581	4.690	9.721	176222
	CMTK C	5.692	3.809	1.732	5.000	10.416	174120
	Elastix A	4.780	3.210	1.581	4.243	8.124	168430
	Elastix B	4.626	2.993	1.581	4.183	7.906	173564
JFRC2013	ANTs A	4.645	3.065	1.581	4.183	7.906	170612
	ANTs B	5.310	3.343	1.732	4.743	9.247	189622
	ANTs C	5.131	3.290	1.732	4.583	8.803	194948
	CMTK A	4.812	3.194	1.581	4.243	8.337	177804
	CMTK B	4.942	3.330	1.581	4.359	8.631	175958
	CMTK C	5.533	3.653	1.732	4.743	10.000	181296
	Elastix A	4.743	3.168	1.581	4.183	8.185	166272
	Elastix B	4.647	3.050	1.581	4.183	7.906	167734
JRC2018	ANTs A	4.610	3.003	1.581	4.183	7.810	173092
	ANTs B	4.915	3.120	1.581	4.472	8.307	172192
	ANTs C	4.939	3.032	1.581	4.528	8.367	180874
	CMTK A	4.701	3.026	1.581	4.243	8.031	170442
	CMTK B	4.726	3.054	1.581	4.243	8.031	166110
	CMTK C	4.775	3.109	1.581	4.301	8.093	166222
	Elastix A	4.690	3.087	1.581	4.183	8.031	167460
	Elastix B	4.603	3.010	1.581	4.123	7.842	168266
FCWB	ANTs A	4.804	2.970	1.581	4.359	8.185	185122
	ANTs B	5.373	3.337	1.732	4.743	9.487	177740
	ANTs C	5.374	3.392	1.732	4.743	9.327	180198
	CMTK A	5.578	3.668	1.732	4.950	9.849	197506
	CMTK B	5.321	3.365	1.732	4.743	9.220	191900
	CMTK C	5.287	3.297	1.732	4.743	9.220	188836
	Elastix A	5.823	4.310	1.732	5.000	10.223	206366
	Elastix B	5.360	3.746	1.581	4.583	9.721	192782
Tefor	ANTs A	4.698	3.037	1.581	4.243	7.906	178492
	ANTs B	5.106	3.190	1.732	4.583	8.631	183638
	ANTs C	5.146	3.204	1.732	4.637	8.746	191264
	CMTK A	5.566	3.793	1.581	4.796	10.149	202582
	CMTK B	5.607	3.896	1.581	4.796	10.440	198986
	CMTK C	5.389	3.535	1.732	4.743	9.407	201050
	Elastix A	4.763	3.052	1.581	4.301	8.031	164536
	Elastix B	4.713	2.886	1.581	4.301	8.031	186088

Table S24. IVLP_R : wedge

Template	Algorithm	Mean	Std dev	10th perc	median	90th perc	N
JFRC2010	ANTs A	8.138	7.834	1.000	5.292	20.012	436308
	ANTs B	3.868	4.330	0.707	2.345	9.301	431512
	ANTs C	4.288	4.362	0.707	2.828	9.950	405618
	CMTK A	4.167	5.672	0.707	2.121	11.000	408410
	CMTK B	4.087	5.280	0.707	2.121	10.464	408162
	CMTK C	4.759	5.428	0.707	2.915	11.619	392684
	Elastix A	3.515	4.163	0.707	2.121	8.337	420146
	Elastix B	3.369	4.185	0.707	2.000	8.307	416076
JFRC2013	ANTs A	3.491	4.434	0.707	2.000	8.631	426466
	ANTs B	4.013	4.123	0.707	2.646	9.220	436614
	ANTs C	3.941	4.192	0.707	2.550	9.220	446714
	CMTK A	4.096	4.460	0.707	2.550	9.925	394782
	CMTK B	4.233	4.546	0.707	2.646	9.925	396906
	CMTK C	4.599	4.552	0.707	3.000	10.724	417880
	Elastix A	3.444	4.263	0.707	2.000	8.485	407894
	Elastix B	3.320	4.261	0.707	1.732	8.276	416212
JRC2018	ANTs A	3.424	4.378	0.707	1.732	8.573	433598
	ANTs B	3.581	4.304	0.707	2.121	8.544	421340
	ANTs C	3.550	4.278	0.707	2.121	8.544	437168
	CMTK A	3.426	4.491	0.707	1.732	8.485	412332
	CMTK B	3.393	4.419	0.707	1.732	8.276	408696
	CMTK C	3.416	4.308	0.707	2.000	8.246	406596
	Elastix A	3.353	4.264	0.707	2.000	8.093	416490
	Elastix B	3.350	4.296	0.707	1.732	8.185	411904
FCWB	ANTs A	3.640	4.550	0.707	2.121	9.000	471818
	ANTs B	4.230	4.373	0.707	2.646	9.849	425034
	ANTs C	4.165	4.300	0.707	2.646	9.460	428146
	CMTK A	6.287	6.209	1.000	4.062	15.067	357542
	CMTK B	5.840	5.666	1.000	3.873	13.342	326860
	CMTK C	4.421	5.056	0.707	2.646	10.700	408458
	Elastix A	6.252	5.924	0.707	4.359	14.491	382002
	Elastix B	4.167	4.870	0.707	2.236	10.886	423918
Tefor	ANTs A	3.436	4.397	0.707	1.732	8.631	443520
	ANTs B	3.818	4.130	0.707	2.345	9.028	435004
	ANTs C	3.974	4.276	0.707	2.449	9.460	434722
	CMTK A	4.141	5.693	0.707	2.121	10.607	405412
	CMTK B	4.286	5.878	0.707	2.121	11.068	406718
	CMTK C	4.370	5.011	0.707	2.550	10.607	418774
	Elastix A	3.531	4.427	0.707	2.000	8.660	421524
	Elastix B	3.351	4.235	0.707	1.732	8.485	416838

Table S25. PLP_R : posterior lateral protocerebrum

Template	Algorithm	Mean	Std dev	10th perc	median	90th perc	N
JFRC2010	ANTs A	13.329	10.282	2.121	10.863	28.328	17992
	ANTs B	7.807	8.316	1.000	4.062	21.331	14808
	ANTs C	8.087	7.943	1.225	5.000	20.917	14984
	CMTK A	8.705	8.213	1.225	5.339	21.953	16612
	CMTK B	9.651	9.371	1.225	5.385	25.159	16454
	CMTK C	9.807	9.479	1.581	5.701	25.060	13412
	Elastix A	8.053	7.717	1.225	5.148	19.873	17078
	Elastix B	7.727	7.692	1.000	4.528	20.075	17320
JFRC2013	ANTs A	7.026	7.092	1.000	4.301	17.479	17460
	ANTs B	6.472	7.142	1.000	3.606	16.703	14228
	ANTs C	6.378	7.364	1.000	3.240	17.944	13420
	CMTK A	10.651	9.174	1.414	7.583	24.208	19620
	CMTK B	10.417	8.960	1.414	7.681	23.633	19254
	CMTK C	10.034	8.353	2.000	7.280	22.372	18320
	Elastix A	7.791	7.466	1.000	5.000	19.085	16856
	Elastix B	7.241	7.267	1.000	4.472	18.083	18682
JRC2018	ANTs A	7.155	7.195	1.000	4.301	17.776	17150
	ANTs B	7.451	7.594	1.000	4.301	19.209	16210
	ANTs C	7.851	7.668	1.225	4.690	20.261	15504
	CMTK A	7.115	6.907	1.000	4.472	17.331	17054
	CMTK B	7.108	7.046	1.000	4.359	17.819	17370
	CMTK C	7.315	7.216	1.000	4.528	18.371	15946
	Elastix A	7.141	7.254	1.000	4.183	18.044	16760
	Elastix B	6.963	6.918	1.000	4.359	17.517	18784
FCWB	ANTs A	7.659	7.969	1.000	4.472	19.456	16620
	ANTs B	7.112	8.124	1.000	4.359	17.972	10226
	ANTs C	8.176	8.485	1.225	5.000	22.683	13788
	CMTK A	9.582	8.345	1.581	6.708	22.159	15260
	CMTK B	9.158	8.109	1.581	6.245	21.691	15174
	CMTK C	7.059	8.317	1.000	3.464	20.846	17600
	Elastix A	8.114	7.969	1.000	4.796	21.483	16684
	Elastix B	6.995	6.898	1.000	4.359	17.751	18760
Tefor	ANTs A	7.077	6.970	1.000	4.359	17.578	17286
	ANTs B	7.911	7.031	1.581	5.701	18.152	13698
	ANTs C	6.938	6.834	1.000	4.472	17.357	17156
	CMTK A	10.065	8.432	1.414	7.416	22.858	19664
	CMTK B	9.895	8.227	1.414	7.280	22.749	19914
	CMTK C	7.788	7.484	1.225	4.583	19.515	23164
	Elastix A	7.023	7.338	1.000	3.808	18.155	18980
	Elastix B	7.508	7.435	1.225	4.528	18.345	19840

Table S26. AOTU_R : anterior optic tubercle

Template	Algorithm	Mean	Std dev	10th perc	median	90th perc	N
JFRC2010	ANTs A	7.788	8.293	1.225	5.000	19.962	113000
	ANTs B	3.837	3.175	0.707	2.915	8.185	105928
	ANTs C	4.132	3.291	0.707	3.240	8.775	103418
	CMTK A	5.092	6.903	0.707	2.915	11.269	84104
	CMTK B	5.052	6.506	0.707	2.915	11.068	85226
	CMTK C	5.238	6.329	1.000	3.162	11.769	98656
	Elastix A	3.411	2.662	0.707	2.915	6.708	94852
	Elastix B	3.594	2.928	0.707	2.915	7.246	88476
JFRC2013	ANTs A	3.239	2.488	0.707	2.646	6.671	101422
	ANTs B	4.387	3.451	1.000	3.536	9.220	99430
	ANTs C	4.344	3.432	1.000	3.464	9.220	97998
	CMTK A	3.365	2.706	0.707	2.646	7.071	84030
	CMTK B	3.425	2.794	0.707	2.646	7.106	86578
	CMTK C	4.234	3.308	1.000	3.536	8.000	99184
	Elastix A	3.355	2.576	0.707	2.646	6.856	92320
	Elastix B	3.236	2.484	0.707	2.646	6.671	90826
JRC2018	ANTs A	3.212	2.472	0.707	2.646	6.557	99712
	ANTs B	3.614	2.878	0.707	2.915	7.616	103552
	ANTs C	3.364	2.580	0.707	2.646	6.856	97756
	CMTK A	3.384	2.692	0.707	2.646	7.141	81058
	CMTK B	3.361	2.638	0.707	2.646	7.071	82722
	CMTK C	3.223	2.431	0.707	2.646	6.481	96996
	Elastix A	3.298	2.399	0.707	2.828	6.481	107920
	Elastix B	3.197	2.385	0.707	2.646	6.442	99008
FCWB	ANTs A	3.224	2.556	0.707	2.550	6.671	97924
	ANTs B	3.967	2.743	1.000	3.317	7.649	94372
	ANTs C	3.714	2.654	1.000	3.082	7.246	94252
	CMTK A	6.950	7.080	1.000	4.301	18.348	67048
	CMTK B	6.223	6.145	1.000	4.062	15.232	80174
	CMTK C	3.882	3.410	0.707	2.915	8.031	102780
	Elastix A	9.584	9.620	1.000	5.523	26.182	86106
	Elastix B	4.043	3.799	0.707	2.915	8.631	99540
Tefor	ANTs A	3.170	2.431	0.707	2.550	6.481	98684
	ANTs B	3.797	3.090	0.707	2.915	8.031	107810
	ANTs C	4.030	3.142	0.707	3.240	8.307	101590
	CMTK A	4.006	4.605	0.707	2.646	8.185	82046
	CMTK B	3.913	4.397	0.707	2.646	8.031	82136
	CMTK C	3.854	3.416	1.000	3.000	7.517	106304
	Elastix A	3.301	2.545	0.707	2.646	6.671	94924
	Elastix B	3.731	2.989	1.000	2.915	7.616	90522

Table S27. GOR_R : gorget

Template	Algorithm	Mean	Std dev	10th perc	median	90th perc	N
JFRC2010	ANTs A	4.842	3.579	1.225	4.000	9.644	449372
	ANTs B	5.478	4.268	1.414	4.359	11.091	284214
	ANTs C	4.769	3.397	1.225	4.062	9.301	374730
	CMTK A	5.117	4.587	1.000	3.808	10.724	364534
	CMTK B	4.714	3.852	1.000	3.674	9.301	360984
	CMTK C	5.814	5.695	1.225	4.183	11.597	292448
	Elastix A	4.104	2.753	1.225	3.536	7.906	353136
	Elastix B	3.748	2.496	1.000	3.240	7.141	385158
JFRC2013	ANTs A	5.162	3.945	1.225	4.123	10.607	309960
	ANTs B	5.486	4.208	1.414	4.359	11.068	312332
	ANTs C	5.548	4.330	1.414	4.359	11.424	305446
	CMTK A	3.896	2.553	1.000	3.317	7.416	238400
	CMTK B	4.203	2.846	1.225	3.606	8.093	228912
	CMTK C	5.285	3.729	1.414	4.472	10.271	184862
	Elastix A	3.729	2.415	1.000	3.240	7.071	319332
	Elastix B	3.576	2.326	1.000	3.082	6.819	328444
JRC2018	ANTs A	3.546	2.446	1.000	2.915	7.000	421666
	ANTs B	4.481	3.102	1.225	3.808	8.660	400478
	ANTs C	4.315	2.930	1.225	3.674	8.276	417118
	CMTK A	3.648	2.524	1.000	3.000	7.106	423462
	CMTK B	3.774	2.534	1.000	3.162	7.280	407944
	CMTK C	3.967	2.564	1.000	3.536	7.517	390700
	Elastix A	4.153	2.860	1.000	3.536	8.093	400538
	Elastix B	3.720	2.532	1.000	3.162	7.211	406424
FCWB	ANTs A	4.132	3.032	1.000	3.464	8.093	315408
	ANTs B	5.556	4.440	1.414	4.359	11.424	262056
	ANTs C	5.035	3.930	1.225	4.062	10.223	304860
	CMTK A	5.056	4.063	1.000	3.873	10.512	375234
	CMTK B	4.835	3.779	1.000	3.808	9.670	370578
	CMTK C	5.338	4.104	1.225	4.301	10.817	240104
	Elastix A	6.264	5.314	1.225	4.583	14.195	267858
	Elastix B	4.907	4.293	1.000	3.606	10.223	308952
Tefor	ANTs A	3.444	2.382	1.000	2.915	6.708	414600
	ANTs B	5.301	4.154	1.225	4.123	11.000	262940
	ANTs C	5.297	4.199	1.225	4.123	11.269	316732
	CMTK A	4.965	4.998	1.000	3.464	10.700	338350
	CMTK B	5.116	5.223	1.000	3.536	10.817	320050
	CMTK C	5.524	4.721	1.225	4.183	11.336	242994
	Elastix A	3.923	2.675	1.000	3.317	7.517	344336
	Elastix B	3.640	2.457	1.000	3.082	7.000	381100

Table S28. MB_CA_R : calyx of adult mushroom body

Template	Algorithm	Mean	Std dev	10th perc	median	90th perc	N
JFRC2010	ANTs A	7.065	5.820	1.581	5.431	15.017	416104
	ANTs B	5.342	4.028	1.414	4.301	10.817	403054
	ANTs C	5.702	4.104	1.414	4.743	11.402	430918
	CMTK A	7.125	6.977	1.414	4.743	16.279	309582
	CMTK B	6.271	5.801	1.414	4.528	13.454	318590
	CMTK C	6.349	5.999	1.414	4.472	13.910	423128
	Elastix A	4.247	3.271	1.000	3.536	8.396	367332
	Elastix B	4.470	3.646	1.000	3.536	9.000	331736
JFRC2013	ANTs A	4.777	3.821	1.000	3.808	9.644	365592
	ANTs B	5.869	4.310	1.581	4.796	11.853	354298
	ANTs C	5.653	4.082	1.581	4.690	11.136	379870
	CMTK A	4.890	3.882	1.225	3.808	10.000	309642
	CMTK B	4.890	3.848	1.225	3.808	9.925	319706
	CMTK C	5.413	4.224	1.414	4.301	11.045	394108
	Elastix A	4.472	3.561	1.000	3.606	8.972	359646
	Elastix B	4.512	3.673	1.000	3.536	9.220	343302
JRC2018	ANTs A	4.609	3.635	1.000	3.606	9.460	365344
	ANTs B	5.140	3.904	1.225	4.123	10.392	368634
	ANTs C	5.137	3.830	1.414	4.183	10.124	366652
	CMTK A	4.998	3.878	1.225	4.062	10.124	313232
	CMTK B	4.955	3.877	1.225	4.000	10.000	311026
	CMTK C	4.998	3.844	1.225	4.062	9.925	329436
	Elastix A	4.650	3.672	1.000	3.674	9.381	353656
	Elastix B	4.652	3.781	1.000	3.606	9.460	333390
FCWB	ANTs A	5.113	3.889	1.225	4.123	10.271	392366
	ANTs B	5.396	4.056	1.414	4.359	10.977	426566
	ANTs C	5.244	3.855	1.414	4.301	10.512	415894
	CMTK A	6.272	5.706	1.414	4.690	13.000	314618
	CMTK B	5.998	5.601	1.414	4.359	12.530	331114
	CMTK C	5.694	4.890	1.414	4.301	12.104	368580
	Elastix A	6.563	5.646	1.414	4.743	14.933	406550
	Elastix B	5.316	4.694	1.000	3.873	11.247	386478
Tefor	ANTs A	4.693	3.644	1.000	3.808	9.539	388030
	ANTs B	5.100	3.892	1.225	4.123	10.247	446012
	ANTs C	5.550	4.065	1.414	4.528	10.977	415622
	CMTK A	5.513	4.931	1.225	4.123	11.597	323322
	CMTK B	5.493	4.770	1.225	4.123	11.424	327944
	CMTK C	5.336	4.139	1.414	4.243	10.724	438296
	Elastix A	4.558	3.545	1.000	3.606	9.000	352978
	Elastix B	4.667	3.701	1.000	3.674	9.301	317442

Table S29. SPS_R : superior posterior slope

Template	Algorithm	Mean	Std dev	10th perc	median	90th perc	N
JFRC2010	ANTs A	8.126	5.735	2.121	6.671	16.401	342866
	ANTs B	6.740	4.685	1.732	5.701	13.342	245712
	ANTs C	7.951	5.083	2.449	6.856	15.182	201122
	CMTK A	7.647	5.359	2.000	6.205	15.668	229274
	CMTK B	7.248	5.118	1.732	5.831	14.983	231834
	CMTK C	7.370	4.969	2.000	6.205	14.714	222776
	Elastix A	4.699	3.819	1.000	3.606	9.925	306590
	Elastix B	4.888	4.050	1.225	3.674	10.416	308932
JFRC2013	ANTs A	5.746	4.481	1.414	4.528	12.166	301130
	ANTs B	7.402	4.879	2.236	6.205	14.577	219492
	ANTs C	7.204	4.724	2.121	6.124	13.946	230954
	CMTK A	5.630	4.317	1.414	4.528	11.576	282136
	CMTK B	5.786	4.335	1.581	4.690	11.705	277218
	CMTK C	6.513	4.554	1.732	5.339	13.134	293938
	Elastix A	4.714	3.943	1.000	3.606	10.000	325016
	Elastix B	4.913	4.014	1.000	3.808	10.512	302166
JRC2018	ANTs A	4.866	3.894	1.225	3.808	10.025	313820
	ANTs B	6.137	4.565	1.581	5.000	12.550	273266
	ANTs C	6.024	4.265	1.581	5.000	11.832	268456
	CMTK A	5.262	4.008	1.414	4.243	10.512	290294
	CMTK B	5.347	4.095	1.414	4.301	10.724	271122
	CMTK C	5.732	4.272	1.581	4.583	11.489	249432
	Elastix A	4.668	3.871	1.000	3.606	9.721	322516
	Elastix B	4.906	3.956	1.225	3.808	10.223	304868
FCWB	ANTs A	5.664	4.176	1.414	4.583	11.424	323078
	ANTs B	6.484	4.423	1.732	5.431	12.728	261148
	ANTs C	6.926	4.620	2.121	5.916	13.229	225102
	CMTK A	7.538	5.218	2.000	6.245	15.248	304856
	CMTK B	6.912	4.943	1.732	5.701	14.053	299012
	CMTK C	6.947	4.662	2.000	5.874	13.675	254194
	Elastix A	7.752	5.436	2.121	6.519	15.297	333362
	Elastix B	6.437	5.103	1.581	5.000	13.583	373960
Tefor	ANTs A	5.070	3.997	1.225	4.062	10.536	320492
	ANTs B	6.728	4.517	2.000	5.701	13.172	265242
	ANTs C	7.193	4.684	2.121	6.124	13.730	240284
	CMTK A	7.271	6.902	1.414	4.796	17.000	316638
	CMTK B	7.316	6.931	1.581	4.950	17.015	312412
	CMTK C	6.579	5.477	1.581	5.000	14.036	346162
	Elastix A	4.592	3.740	1.000	3.606	9.513	324824
	Elastix B	4.867	3.865	1.225	3.808	10.025	317084

Table S30. IPS_R : inferior posterior slope

Template	Algorithm	Mean	Std dev	10th perc	median	90th perc	N
JFRC2010	ANTs A	6.050	5.921	0.707	3.873	14.491	315090
	ANTs B	2.961	3.347	0.707	1.732	6.856	335148
	ANTs C	3.032	3.282	0.707	2.000	6.964	312856
	CMTK A	3.547	5.024	0.707	1.581	9.899	333098
	CMTK B	3.487	4.911	0.707	1.581	9.110	333226
	CMTK C	3.763	4.899	0.707	2.000	9.460	325342
	Elastix A	2.674	3.202	0.707	1.581	6.042	325408
	Elastix B	2.968	3.756	0.707	1.581	7.036	327182
JFRC2013	ANTs A	2.535	2.978	0.707	1.581	5.745	345434
	ANTs B	3.090	3.512	0.707	1.732	7.314	316090
	ANTs C	3.092	3.441	0.707	2.000	7.211	326860
	CMTK A	2.849	3.231	0.707	1.732	6.164	343224
	CMTK B	3.025	3.283	0.707	2.121	6.519	338554
	CMTK C	3.707	3.545	0.707	2.646	7.937	299000
	Elastix A	2.616	3.048	0.707	1.581	5.874	339380
	Elastix B	2.640	3.116	0.707	1.581	5.831	352042
JRC2018	ANTs A	2.446	2.855	0.707	1.581	5.431	347798
	ANTs B	2.720	2.903	0.707	1.732	5.874	345134
	ANTs C	2.757	2.925	0.707	1.732	6.083	351358
	CMTK A	2.463	2.939	0.707	1.581	5.431	346020
	CMTK B	2.466	2.979	0.707	1.581	5.431	346934
	CMTK C	2.513	3.020	0.707	1.581	5.568	343496
	Elastix A	2.603	3.102	0.707	1.581	5.701	340310
	Elastix B	2.645	3.127	0.707	1.581	5.831	348782
FCWB	ANTs A	3.277	3.556	0.707	2.121	7.517	301648
	ANTs B	3.632	3.923	0.707	2.236	8.631	288116
	ANTs C	3.550	3.730	0.707	2.236	8.276	304246
	CMTK A	4.284	4.827	0.707	2.550	10.654	244114
	CMTK B	4.021	4.340	0.707	2.550	9.434	250492
	CMTK C	3.317	3.993	0.707	2.000	7.416	317350
	Elastix A	4.578	5.212	0.707	2.550	11.832	270764
	Elastix B	3.208	4.433	0.707	1.732	7.211	316894
Tefor	ANTs A	2.496	2.879	0.707	1.581	5.523	349796
	ANTs B	2.749	3.009	0.707	1.732	6.124	334934
	ANTs C	2.853	3.174	0.707	1.732	6.442	345290
	CMTK A	3.185	4.757	0.707	1.581	7.036	336610
	CMTK B	3.206	4.688	0.707	1.581	7.517	331342
	CMTK C	3.161	4.003	0.707	2.000	6.708	339266
	Elastix A	2.631	3.229	0.707	1.581	5.874	334922
	Elastix B	2.947	3.491	0.707	1.732	7.000	321002

Table S31. SCL_R : superior clamp

Template	Algorithm	Mean	Std dev	10th perc	median	90th perc	N
JFRC2010	ANTs A	8.079	8.073	1.581	5.196	19.300	765240
	ANTs B	5.244	4.284	1.581	4.183	9.950	635108
	ANTs C	5.892	4.745	1.581	4.583	11.937	612046
	CMTK A	8.762	7.817	1.732	5.874	20.857	674518
	CMTK B	7.278	6.555	1.581	5.148	16.202	633590
	CMTK C	7.782	6.612	1.732	5.745	16.523	709042
	Elastix A	4.646	3.713	1.414	3.808	8.515	600342
	Elastix B	4.479	3.533	1.414	3.674	8.185	611184
JFRC2013	ANTs A	4.590	4.037	1.225	3.606	8.515	630492
	ANTs B	5.339	4.529	1.581	4.123	10.512	625254
	ANTs C	5.221	4.461	1.414	4.123	10.025	601610
	CMTK A	6.059	5.748	1.581	4.243	13.304	689216
	CMTK B	6.337	6.124	1.581	4.359	14.457	681688
	CMTK C	5.830	4.451	1.581	4.583	11.790	660042
	Elastix A	4.439	3.599	1.225	3.606	7.937	639570
	Elastix B	4.400	3.598	1.225	3.606	7.937	654960
JRC2018	ANTs A	4.368	3.696	1.225	3.606	7.906	633808
	ANTs B	4.542	3.821	1.414	3.606	8.307	649512
	ANTs C	4.659	3.911	1.414	3.808	8.544	633012
	CMTK A	4.361	3.625	1.225	3.606	7.906	650942
	CMTK B	4.359	3.594	1.225	3.606	7.906	628218
	CMTK C	4.443	3.657	1.225	3.606	8.062	623624
	Elastix A	4.386	3.570	1.225	3.606	7.842	649538
	Elastix B	4.541	3.737	1.414	3.674	8.185	644400
FCWB	ANTs A	4.659	4.054	1.225	3.606	8.631	629916
	ANTs B	5.370	4.123	1.581	4.359	10.025	738100
	ANTs C	6.037	4.515	1.732	4.950	11.597	704222
	CMTK A	8.843	7.562	1.732	6.164	20.408	621926
	CMTK B	7.667	6.647	1.732	5.339	17.692	579792
	CMTK C	6.573	5.154	1.732	5.099	13.620	739520
	Elastix A	8.836	7.008	2.121	6.708	18.520	766336
	Elastix B	7.715	7.530	1.581	5.148	17.776	692590
Tefor	ANTs A	4.395	3.671	1.225	3.606	8.031	639674
	ANTs B	4.926	4.055	1.414	3.873	9.220	612498
	ANTs C	5.096	4.334	1.414	4.062	9.644	582010
	CMTK A	7.184	6.627	1.581	4.796	16.688	722676
	CMTK B	7.202	6.647	1.581	4.950	16.583	714756
	CMTK C	6.569	5.881	1.581	4.690	14.577	613750
	Elastix A	4.570	3.677	1.414	3.808	8.307	597264
	Elastix B	4.457	3.577	1.225	3.606	8.093	613856

Table S32. LO_R : lobula

Template	Algorithm	Mean	Std dev	10th perc	median	90th perc	N
JFRC2010	ANTs A	10.110	9.192	1.581	7.000	24.870	20032
	ANTs B	5.442	5.156	1.000	3.606	13.229	15862
	ANTs C	7.146	6.935	1.000	4.123	18.507	13508
	CMTK A	6.936	9.153	1.000	3.240	18.180	12426
	CMTK B	6.429	8.057	1.000	3.240	15.716	12974
	CMTK C	6.017	6.591	1.000	3.536	15.379	10854
	Elastix A	3.909	3.004	1.000	3.162	7.810	16124
	Elastix B	4.172	2.910	1.000	3.536	8.307	22564
JFRC2013	ANTs A	3.902	3.014	1.000	3.000	8.276	20466
	ANTs B	7.986	6.650	1.732	5.568	19.157	10842
	ANTs C	8.399	6.698	1.581	5.701	18.722	12826
	CMTK A	3.623	2.706	1.000	2.915	7.649	16792
	CMTK B	3.685	2.840	1.000	2.915	7.681	17748
	CMTK C	5.969	5.275	1.225	4.359	12.590	16420
	Elastix A	3.793	2.980	1.000	2.915	8.031	21572
	Elastix B	3.781	3.007	1.000	2.915	8.093	21214
JRC2018	ANTs A	3.883	2.876	1.000	3.082	8.031	20550
	ANTs B	4.840	4.506	1.000	3.240	11.023	19126
	ANTs C	4.767	4.333	1.000	3.536	10.000	16668
	CMTK A	3.478	2.658	0.707	2.915	7.036	17250
	CMTK B	3.395	2.573	0.707	2.646	7.036	17552
	CMTK C	3.707	2.808	1.000	2.915	7.906	18484
	Elastix A	3.956	3.037	1.000	3.000	8.396	18924
	Elastix B	3.709	2.816	1.000	2.915	7.649	19554
FCWB	ANTs A	4.427	3.643	1.000	3.240	9.407	18974
	ANTs B	5.636	4.773	1.414	4.183	12.266	20592
	ANTs C	5.450	4.966	1.000	3.873	12.207	20774
	CMTK A	5.640	5.752	1.000	3.808	12.826	11616
	CMTK B	6.464	5.187	1.414	5.050	12.981	14828
	CMTK C	5.664	5.591	1.000	3.536	13.601	20246
	Elastix A	11.371	8.555	1.225	10.392	23.611	14924
	Elastix B	7.076	5.746	1.000	5.196	15.588	27258
Tefor	ANTs A	3.765	2.803	1.000	3.000	7.906	20450
	ANTs B	6.679	6.131	1.000	4.301	15.906	15576
	ANTs C	6.193	5.221	1.414	4.610	13.366	14578
	CMTK A	5.613	8.651	0.707	2.646	10.794	15534
	CMTK B	5.580	8.523	0.707	2.646	10.607	16134
	CMTK C	5.280	4.433	1.000	4.000	11.180	20264
	Elastix A	4.129	3.043	1.000	3.240	8.746	19768
	Elastix B	4.306	3.201	1.000	3.536	8.718	20036

Table S33. EPA_R : epaulette

Template	Algorithm	Mean	Std dev	10th perc	median	90th perc	N
JFRC2010	ANTs A	7.024	5.606	1.732	5.568	13.802	1889608
	ANTs B	6.817	4.626	2.121	5.788	12.748	1696632
	ANTs C	6.951	4.770	2.121	5.874	13.077	1812676
	CMTK A	6.567	4.732	1.732	5.431	12.767	1859654
	CMTK B	6.409	4.684	1.732	5.292	12.369	1736788
	CMTK C	6.917	4.713	2.121	5.874	13.000	1711540
	Elastix A	5.671	4.258	1.581	4.583	11.045	1588272
	Elastix B	5.702	4.328	1.581	4.583	11.180	1604856
JFRC2013	ANTs A	5.917	4.639	1.581	4.743	11.853	1676118
	ANTs B	7.171	5.031	2.121	6.000	13.601	1611632
	ANTs C	7.246	5.104	2.121	6.083	13.802	1658494
	CMTK A	5.859	4.324	1.581	4.796	11.336	1614130
	CMTK B	5.933	4.318	1.581	4.950	11.402	1613038
	CMTK C	6.907	4.692	2.121	5.831	13.058	1507918
	Elastix A	5.507	4.373	1.581	4.359	10.863	1641038
	Elastix B	5.450	4.396	1.414	4.301	10.817	1621388
JRC2018	ANTs A	5.419	4.431	1.414	4.243	10.886	1645444
	ANTs B	6.766	4.646	2.000	5.701	12.826	1637668
	ANTs C	6.908	4.798	2.121	5.788	13.115	1688056
	CMTK A	5.416	4.405	1.414	4.243	10.817	1654410
	CMTK B	5.454	4.421	1.581	4.301	10.817	1601386
	CMTK C	5.715	4.440	1.581	4.528	11.136	1595976
	Elastix A	5.504	4.367	1.581	4.359	10.817	1570950
	Elastix B	5.533	4.386	1.581	4.359	10.886	1596056
FCWB	ANTs A	5.715	4.390	1.581	4.583	11.180	1668296
	ANTs B	6.499	4.468	2.121	5.523	12.166	1722224
	ANTs C	6.654	4.623	2.121	5.568	12.510	1752242
	CMTK A	6.772	4.818	2.000	5.701	13.000	1771876
	CMTK B	6.638	4.791	2.000	5.523	12.689	1691152
	CMTK C	6.546	4.528	2.000	5.523	12.590	1636296
	Elastix A	7.302	5.378	2.121	6.083	13.802	1681564
	Elastix B	6.459	4.579	1.732	5.385	12.410	1917728
Tefor	ANTs A	5.610	4.474	1.581	4.472	11.158	1660500
	ANTs B	7.064	4.853	2.121	6.000	13.304	1680796
	ANTs C	7.435	5.237	2.121	6.205	14.213	1731516
	CMTK A	6.500	5.088	1.581	5.148	13.210	1815200
	CMTK B	6.564	5.118	1.581	5.148	13.285	1809270
	CMTK C	7.222	5.560	2.121	5.831	13.892	1738810
	Elastix A	5.597	4.333	1.581	4.528	10.977	1646730
	Elastix B	6.153	4.573	1.581	5.000	12.062	1619762

Table S34. GNG : adult gnathal ganglion

Template	Algorithm	Mean	Std dev	10th perc	median	90th perc	N
JFRC2010	ANTs A	5.224	4.249	1.000	4.062	10.977	43164
	ANTs B	4.061	3.022	1.000	3.464	7.906	51038
	ANTs C	4.322	3.015	1.225	3.674	8.093	51848
	CMTK A	5.213	3.991	1.414	4.183	10.271	53416
	CMTK B	4.750	3.427	1.225	4.062	9.028	52090
	CMTK C	4.946	3.577	1.414	4.123	9.301	56954
	Elastix A	3.603	2.707	1.000	3.000	6.745	50786
	Elastix B	3.363	2.412	1.000	2.915	6.124	52898
JFRC2013	ANTs A	3.577	2.998	1.000	2.915	6.964	43970
	ANTs B	4.209	2.729	1.414	3.808	7.550	60532
	ANTs C	4.321	2.589	1.414	4.000	7.778	59536
	CMTK A	3.938	3.282	1.000	3.162	7.616	38334
	CMTK B	3.967	3.216	1.000	3.240	7.616	39398
	CMTK C	3.997	2.608	1.225	3.606	7.141	57542
	Elastix A	3.657	2.926	1.000	3.000	7.000	55624
	Elastix B	3.664	2.828	1.000	3.000	7.036	48880
JRC2018	ANTs A	3.536	2.998	0.707	2.915	6.928	44366
	ANTs B	3.941	3.021	1.000	3.240	7.616	47364
	ANTs C	4.016	3.119	1.000	3.317	7.649	43868
	CMTK A	4.200	3.509	1.000	3.536	7.937	33850
	CMTK B	4.138	3.534	1.000	3.317	7.906	34784
	CMTK C	3.831	3.216	1.000	3.082	7.382	39328
	Elastix A	3.709	2.918	1.000	3.000	7.071	48002
	Elastix B	3.757	2.986	1.000	3.082	7.106	43726
FCWB	ANTs A	3.741	3.325	0.707	2.915	7.314	41796
	ANTs B	3.957	2.289	1.414	3.606	7.106	63002
	ANTs C	4.189	2.394	1.414	3.808	7.517	59292
	CMTK A	5.897	4.179	1.581	5.339	10.271	67418
	CMTK B	5.304	3.810	1.414	4.528	9.925	48736
	CMTK C	4.471	2.809	1.414	4.062	8.062	55758
	Elastix A	4.392	2.908	1.225	3.808	8.307	98264
	Elastix B	4.422	3.122	1.225	3.808	8.367	61480
Tefor	ANTs A	3.479	2.937	0.707	2.915	6.745	44098
	ANTs B	4.101	3.041	1.000	3.536	7.906	50334
	ANTs C	4.229	3.220	1.000	3.536	8.276	45398
	CMTK A	4.588	3.424	1.225	3.808	8.868	42418
	CMTK B	4.754	3.476	1.225	4.062	9.110	44458
	CMTK C	4.581	3.167	1.225	3.873	8.746	58062
	Elastix A	3.498	2.564	1.000	3.000	6.442	52472
	Elastix B	3.616	2.450	1.000	3.162	6.557	57842

Table S35. NO : nodulus

Template	Algorithm	Mean	Std dev	10th perc	median	90th perc	N
JFRC2010	ANTs A	5.451	6.308	1.414	3.873	8.660	410994
	ANTs B	4.951	3.782	1.581	4.301	8.544	390412
	ANTs C	4.836	3.714	1.581	4.243	8.337	452552
	CMTK A	4.467	4.126	1.225	3.674	7.517	417922
	CMTK B	4.306	3.549	1.414	3.808	7.280	410878
	CMTK C	4.613	3.522	1.414	4.062	7.906	383418
	Elastix A	3.839	3.271	1.000	3.464	6.557	452338
	Elastix B	3.936	3.128	1.000	3.536	6.671	448592
JFRC2013	ANTs A	5.287	4.569	1.414	4.123	10.583	368712
	ANTs B	7.394	5.886	2.000	5.523	16.016	197600
	ANTs C	6.672	5.305	1.732	5.099	14.036	215962
	CMTK A	4.308	3.505	1.000	3.674	7.550	425532
	CMTK B	4.414	3.515	1.225	3.808	7.746	402130
	CMTK C	5.602	4.139	1.581	4.583	10.817	245386
	Elastix A	3.935	3.234	1.000	3.536	6.671	416116
	Elastix B	3.938	3.324	1.000	3.536	6.708	419422
JRC2018	ANTs A	3.771	3.098	1.000	3.317	6.403	452490
	ANTs B	5.119	4.005	1.581	4.301	9.381	385182
	ANTs C	5.495	4.301	1.581	4.528	10.247	403048
	CMTK A	3.694	3.086	1.000	3.240	6.245	467804
	CMTK B	3.749	3.136	1.000	3.317	6.364	458750
	CMTK C	3.860	3.118	1.000	3.536	6.519	443426
	Elastix A	3.770	3.107	1.000	3.464	6.364	441406
	Elastix B	3.861	3.516	1.000	3.464	6.481	435064
FCWB	ANTs A	4.263	3.615	1.000	3.808	7.314	460912
	ANTs B	6.585	5.218	1.732	5.099	13.675	280454
	ANTs C	7.174	6.479	1.581	5.000	16.763	341950
	CMTK A	4.270	3.651	1.000	3.674	7.649	463556
	CMTK B	4.281	3.740	1.000	3.808	7.550	449328
	CMTK C	4.521	3.681	1.000	3.808	8.246	373002
	Elastix A	4.855	4.014	1.225	4.183	8.660	408028
	Elastix B	4.065	3.510	1.000	3.606	7.106	532386
Tefor	ANTs A	3.862	3.146	1.000	3.536	6.557	446818
	ANTs B	5.076	4.005	1.581	4.301	8.944	387362
	ANTs C	5.220	4.459	1.581	4.243	9.220	421106
	CMTK A	4.090	3.315	1.000	3.606	7.106	457468
	CMTK B	4.399	3.671	1.225	3.674	7.681	445144
	CMTK C	5.448	5.667	1.581	4.183	8.631	389430
	Elastix A	3.905	3.120	1.225	3.536	6.557	424520
	Elastix B	4.507	3.591	1.414	3.808	7.778	394342

Table S36. PRW : prow

Template	Algorithm	Mean	Std dev	10th perc	median	90th perc	N
JFRC2010	ANTs A	8.650	6.583	3.240	7.280	13.058	12776
	ANTs B	6.726	3.628	2.121	6.325	11.853	5332
	ANTs C	6.919	3.736	2.236	6.442	12.124	5164
	CMTK A	8.269	4.077	3.240	7.921	13.675	4902
	CMTK B	8.348	4.733	2.828	7.649	15.100	5636
	CMTK C	8.120	4.036	3.082	7.649	13.802	4300
	Elastix A	7.205	4.097	2.121	6.671	12.683	5184
	Elastix B	7.425	4.349	2.000	7.106	13.019	5252
JFRC2013	ANTs A	6.483	3.928	1.732	5.745	11.937	6140
	ANTs B	7.392	3.944	2.636	6.856	13.058	5440
	ANTs C	7.707	3.666	3.082	7.280	12.865	5362
	CMTK A	7.079	3.788	2.550	6.557	12.186	6426
	CMTK B	7.120	4.025	2.449	6.519	12.530	6260
	CMTK C	8.871	3.816	3.606	8.888	13.675	4532
	Elastix A	6.524	3.889	2.121	5.831	11.726	5546
	Elastix B	6.757	4.029	2.121	6.083	12.104	5690
JRC2018	ANTs A	6.542	4.016	1.732	5.745	12.104	6208
	ANTs B	6.646	4.030	1.732	5.895	12.166	6202
	ANTs C	6.856	4.018	2.121	6.083	12.367	6582
	CMTK A	6.834	4.189	2.121	6.083	12.245	5732
	CMTK B	6.755	4.151	2.000	5.916	12.430	6162
	CMTK C	6.748	4.089	2.121	6.083	12.186	5888
	Elastix A	6.671	4.026	2.000	6.000	12.104	5226
	Elastix B	7.546	4.414	2.345	6.708	13.600	5462
FCWB	ANTs A	6.297	3.421	2.000	5.916	11.136	5590
	ANTs B	8.126	4.053	2.550	8.031	13.611	3196
	ANTs C	8.335	4.082	2.915	8.124	13.946	4078
	CMTK A	7.034	4.278	2.345	6.124	12.767	6136
	CMTK B	6.829	4.076	2.236	6.083	12.124	5632
	CMTK C	7.609	3.586	3.000	7.550	12.349	4046
	Elastix A	8.167	4.621	2.646	7.550	14.526	6008
	Elastix B	7.350	4.343	2.236	6.442	13.601	5688
Tefor	ANTs A	6.669	4.165	1.732	5.831	12.104	6206
	ANTs B	6.988	4.027	2.000	6.442	12.081	6332
	ANTs C	7.073	3.991	2.236	6.557	12.590	7242
	CMTK A	7.247	3.744	2.449	7.000	12.166	7508
	CMTK B	7.146	3.944	2.550	6.557	12.410	6372
	CMTK C	7.636	3.922	2.828	7.106	12.748	5630
	Elastix A	6.943	4.088	2.000	6.519	12.349	5154
	Elastix B	7.311	4.060	2.345	6.856	12.826	5682

Table S37. GA_R : gall

Template	Algorithm	Mean	Std dev	10th perc	median	90th perc	N
JFRC2010	ANTs A	6.332	6.591	1.414	4.183	15.556	30288
	ANTs B	4.387	2.400	1.581	4.123	7.649	26644
	ANTs C	4.460	2.496	1.581	4.183	7.778	27714
	CMTK A	5.377	4.685	1.414	4.123	10.100	29566
	CMTK B	5.202	4.786	1.414	4.000	9.381	32256
	CMTK C	6.248	5.083	2.000	4.950	11.958	23192
	Elastix A	4.103	2.433	1.225	3.808	7.382	37556
	Elastix B	4.014	2.260	1.225	3.808	7.106	32506
JFRC2013	ANTs A	3.770	2.246	1.225	3.536	6.819	31780
	ANTs B	4.478	2.626	1.581	4.123	7.810	20492
	ANTs C	4.489	2.555	1.581	4.123	8.016	24386
	CMTK A	4.145	2.878	1.225	3.606	7.382	33056
	CMTK B	4.430	2.991	1.414	3.808	8.031	31064
	CMTK C	4.468	2.792	1.581	4.062	7.550	24506
	Elastix A	3.665	2.178	1.000	3.317	6.557	35180
	Elastix B	3.831	2.174	1.225	3.606	6.708	34084
JRC2018	ANTs A	3.791	2.250	1.000	3.536	6.964	34898
	ANTs B	3.872	2.352	1.000	3.536	7.106	32926
	ANTs C	4.057	2.519	1.000	3.606	7.416	32226
	CMTK A	3.733	2.176	1.225	3.464	6.671	35186
	CMTK B	3.774	2.195	1.225	3.464	6.745	35516
	CMTK C	3.781	2.232	1.000	3.536	6.856	35504
	Elastix A	3.832	2.249	1.225	3.536	6.856	36706
	Elastix B	3.967	2.208	1.414	3.674	6.964	34706
FCWB	ANTs A	3.977	2.203	1.225	3.808	6.964	30514
	ANTs B	4.686	2.763	1.581	4.243	8.307	18914
	ANTs C	4.556	2.921	1.225	4.062	8.544	20840
	CMTK A	4.182	2.362	1.414	3.808	7.382	31992
	CMTK B	4.035	2.340	1.414	3.606	7.246	31278
	CMTK C	5.682	4.936	1.581	4.359	10.536	18984
	Elastix A	5.871	6.539	1.581	4.472	10.794	27614
	Elastix B	4.667	6.330	1.225	3.606	7.382	36610
Tefor	ANTs A	3.938	2.297	1.225	3.606	7.106	31502
	ANTs B	4.125	2.447	1.225	3.808	7.517	30136
	ANTs C	4.171	2.517	1.225	3.808	7.616	31770
	CMTK A	4.897	3.875	1.414	3.873	9.487	33304
	CMTK B	4.968	3.794	1.414	4.062	9.747	33040
	CMTK C	4.499	3.312	1.225	3.606	8.986	27266
	Elastix A	4.147	2.548	1.000	3.808	7.649	34694
	Elastix B	4.103	2.512	1.225	3.674	7.550	33598

Table S38. AME_L : accessory medulla

Template	Algorithm	Mean	Std dev	10th perc	median	90th perc	N
JFRC2010	ANTs A	8.731	7.644	1.732	6.083	20.100	622814
	ANTs B	5.583	4.275	1.581	4.583	10.512	661486
	ANTs C	5.919	4.652	1.581	4.743	11.533	681546
	CMTK A	8.658	7.349	1.732	6.124	19.736	686186
	CMTK B	7.789	6.776	1.581	5.523	18.028	664092
	CMTK C	7.343	5.857	1.732	5.701	15.264	772914
	Elastix A	5.059	3.798	1.581	4.301	9.247	609834
	Elastix B	4.918	3.891	1.581	4.123	9.000	589106
JFRC2013	ANTs A	4.978	4.250	1.414	4.000	9.434	641886
	ANTs B	5.418	4.306	1.581	4.359	10.392	642512
	ANTs C	5.490	4.560	1.581	4.359	10.536	632428
	CMTK A	6.465	7.065	1.581	4.472	13.304	662426
	CMTK B	6.590	7.125	1.581	4.528	13.620	653836
	CMTK C	5.806	4.269	1.581	4.743	11.269	670968
	Elastix A	4.868	3.992	1.414	4.000	9.110	629478
	Elastix B	4.810	4.067	1.414	3.873	8.905	641258
JRC2018	ANTs A	4.845	4.067	1.414	3.873	9.000	634598
	ANTs B	4.940	4.083	1.414	4.062	9.192	642236
	ANTs C	5.280	4.354	1.581	4.301	9.849	638966
	CMTK A	4.785	4.034	1.414	3.808	8.775	627192
	CMTK B	4.766	3.958	1.414	3.873	8.746	613616
	CMTK C	4.864	4.034	1.414	4.000	8.972	616346
	Elastix A	4.799	3.924	1.414	3.873	8.746	643384
	Elastix B	5.128	4.489	1.414	4.123	9.487	641146
FCWB	ANTs A	5.162	4.383	1.414	4.123	9.874	623178
	ANTs B	5.577	4.106	1.581	4.637	10.392	712332
	ANTs C	5.870	4.359	1.581	4.950	10.977	704666
	CMTK A	8.595	7.030	2.000	6.364	18.974	632218
	CMTK B	7.645	6.496	1.732	5.568	16.823	590680
	CMTK C	7.306	6.005	2.000	5.523	15.330	701258
	Elastix A	10.169	8.568	2.236	7.746	20.797	836600
	Elastix B	9.163	10.367	1.732	5.701	20.616	604174
Tefor	ANTs A	4.861	4.035	1.414	3.873	9.055	634782
	ANTs B	5.207	4.293	1.581	4.183	9.721	610874
	ANTs C	5.611	4.759	1.581	4.472	10.724	594648
	CMTK A	7.049	6.689	1.581	4.899	16.047	686944
	CMTK B	7.033	6.672	1.581	4.899	16.140	677718
	CMTK C	6.696	6.132	1.581	4.950	14.300	606444
	Elastix A	4.887	3.901	1.414	4.062	9.055	609784
	Elastix B	4.872	3.934	1.414	4.062	9.000	599672

Table S39. LO_L : lobula

Template	Algorithm	Mean	Std dev	10th perc	median	90th perc	N
JFRC2010	ANTs A	7.689	7.344	2.000	5.701	12.845	20148
	ANTs B	5.515	3.217	1.732	5.148	9.747	10686
	ANTs C	6.080	3.447	1.732	5.745	10.700	9926
	CMTK A	8.695	6.939	1.732	6.403	19.853	8478
	CMTK B	8.106	6.006	2.121	6.364	17.734	9118
	CMTK C	9.887	6.314	3.606	8.031	20.287	4994
	Elastix A	6.345	3.739	2.121	5.788	11.023	10304
	Elastix B	6.434	5.257	1.414	4.796	14.521	12294
JFRC2013	ANTs A	4.800	3.118	1.414	4.183	9.165	13144
	ANTs B	5.842	3.593	1.732	5.148	10.675	14382
	ANTs C	5.230	2.962	1.732	4.743	9.110	14040
	CMTK A	4.605	3.110	1.414	4.000	8.746	14896
	CMTK B	4.467	3.068	1.225	3.674	8.726	16488
	CMTK C	4.619	3.180	1.414	3.873	8.660	21730
	Elastix A	4.863	3.062	1.581	4.359	8.860	15960
	Elastix B	4.817	3.138	1.414	4.301	8.860	15224
JRC2018	ANTs A	4.877	3.062	1.414	4.301	9.192	11708
	ANTs B	4.876	3.135	1.414	4.301	9.110	13174
	ANTs C	5.203	3.239	1.581	4.528	9.487	11572
	CMTK A	4.662	3.097	1.414	4.062	8.803	15284
	CMTK B	4.667	3.142	1.414	4.062	8.972	15040
	CMTK C	4.638	3.055	1.414	4.062	8.803	15242
	Elastix A	4.895	3.078	1.581	4.359	8.746	14802
	Elastix B	4.942	3.323	1.414	4.243	9.407	15086
FCWB	ANTs A	5.045	3.134	1.581	4.528	9.220	10884
	ANTs B	6.541	4.049	1.732	5.874	12.268	9206
	ANTs C	6.165	3.632	2.000	5.745	10.462	8972
	CMTK A	7.467	5.148	1.732	6.557	15.379	9084
	CMTK B	7.050	5.135	1.581	5.874	14.782	11732
	CMTK C	5.562	3.968	1.414	4.472	11.151	15624
	Elastix A	8.932	5.869	1.732	8.170	17.270	7836
	Elastix B	10.732	8.484	2.121	8.631	24.052	5380
Tefor	ANTs A	4.979	3.086	1.581	4.359	9.487	11456
	ANTs B	5.660	3.355	1.732	5.148	10.075	10102
	ANTs C	5.951	3.286	2.121	5.523	10.536	10986
	CMTK A	7.099	6.071	1.581	5.196	15.362	11576
	CMTK B	7.159	5.585	1.581	5.568	15.524	11120
	CMTK C	6.302	3.811	1.732	5.568	11.853	12474
	Elastix A	6.509	3.890	2.121	6.000	11.351	10114
	Elastix B	7.278	5.157	1.732	6.000	14.933	8990

Table S40. BU_L : bulb

Template	Algorithm	Mean	Std dev	10th perc	median	90th perc	N
JFRC2010	ANTs A	6.131	4.686	1.581	5.000	12.104	273626
	ANTs B	5.764	4.971	1.414	4.528	11.402	271974
	ANTs C	6.337	5.179	1.581	5.000	12.430	282188
	CMTK A	6.438	4.694	1.581	5.339	12.826	438598
	CMTK B	6.349	4.623	1.581	5.196	12.530	401034
	CMTK C	7.004	4.965	1.732	5.788	13.946	397448
	Elastix A	4.973	3.639	1.414	4.183	9.327	269426
	Elastix B	5.390	3.897	1.581	4.528	10.271	277598
JFRC2013	ANTs A	4.914	3.565	1.225	4.183	9.327	305420
	ANTs B	6.759	5.441	1.732	5.431	13.077	268188
	ANTs C	6.399	5.541	1.581	5.000	12.530	295292
	CMTK A	5.221	3.812	1.414	4.359	10.124	329340
	CMTK B	5.047	3.592	1.414	4.243	9.670	321452
	CMTK C	5.661	4.459	1.225	4.528	11.511	344122
	Elastix A	4.969	3.514	1.414	4.183	9.460	272544
	Elastix B	4.888	3.525	1.414	4.123	9.220	295686
JRC2018	ANTs A	4.787	3.415	1.225	4.123	9.028	320566
	ANTs B	5.584	4.404	1.414	4.528	10.794	297424
	ANTs C	5.671	4.244	1.581	4.690	11.068	326468
	CMTK A	4.845	3.348	1.414	4.183	9.000	322432
	CMTK B	4.854	3.356	1.414	4.183	9.028	309956
	CMTK C	5.107	3.580	1.414	4.359	9.618	297512
	Elastix A	4.932	3.403	1.414	4.243	9.220	286506
	Elastix B	5.025	3.441	1.414	4.359	9.301	300684
FCWB	ANTs A	5.304	3.605	1.414	4.583	10.050	333478
	ANTs B	6.691	5.234	1.732	5.431	12.590	251700
	ANTs C	6.766	5.062	1.732	5.568	13.229	280818
	CMTK A	6.283	5.506	1.581	5.000	11.489	283790
	CMTK B	5.714	4.789	1.581	4.743	10.223	292828
	CMTK C	6.162	4.331	1.732	5.196	11.619	272040
	Elastix A	7.355	5.445	1.732	6.000	14.933	240540
	Elastix B	6.728	6.016	1.581	5.148	13.210	300976
Tefor	ANTs A	4.792	3.468	1.225	4.123	9.028	318946
	ANTs B	5.730	4.661	1.414	4.583	11.091	292186
	ANTs C	6.158	5.294	1.581	4.796	11.853	323302
	CMTK A	7.814	9.068	1.414	4.690	19.812	270274
	CMTK B	8.033	9.262	1.414	4.743	21.083	266200
	CMTK C	6.962	7.775	1.414	4.743	13.675	283214
	Elastix A	4.958	3.537	1.414	4.183	9.327	291076
	Elastix B	5.504	3.924	1.581	4.690	10.512	291692

Table S41. LH_L : lateral horn

Template	Algorithm	Mean	Std dev	10th perc	median	90th perc	N
JFRC2010	ANTs A	10.240	6.320	2.345	9.721	19.118	121430
	ANTs B	5.447	4.414	1.581	4.301	10.909	73844
	ANTs C	5.306	3.819	1.581	4.472	10.247	74074
	CMTK A	8.578	5.994	2.121	7.000	17.464	100410
	CMTK B	8.907	5.981	2.236	7.616	17.521	86462
	CMTK C	7.021	5.576	1.581	5.148	15.313	135452
	Elastix A	5.717	5.143	1.581	4.183	12.288	55186
	Elastix B	5.670	4.622	1.581	4.359	11.937	63698
JFRC2013	ANTs A	5.205	5.115	1.000	3.606	11.705	54360
	ANTs B	7.383	4.926	2.121	6.205	14.577	91644
	ANTs C	6.899	4.498	2.000	5.874	13.342	72940
	CMTK A	6.475	5.635	1.581	4.583	14.577	43294
	CMTK B	7.081	5.565	1.581	5.431	14.883	47452
	CMTK C	5.981	4.454	1.581	4.796	12.207	89800
	Elastix A	5.019	4.734	1.225	3.606	10.392	63628
	Elastix B	4.886	4.696	1.225	3.536	10.025	63058
JRC2018	ANTs A	5.203	5.151	1.000	3.606	11.853	53098
	ANTs B	5.453	4.800	1.414	4.123	11.533	56144
	ANTs C	5.274	4.182	1.581	4.243	9.892	71594
	CMTK A	5.436	5.279	1.225	3.808	12.369	39660
	CMTK B	5.468	5.254	1.225	3.873	12.186	39286
	CMTK C	5.667	5.387	1.225	4.062	12.530	37522
	Elastix A	5.682	5.225	1.414	4.123	12.669	44346
	Elastix B	5.567	4.978	1.414	4.123	12.042	45054
FCWB	ANTs A	6.462	4.882	1.581	5.148	13.509	51556
	ANTs B	6.121	4.137	1.581	5.148	12.021	120738
	ANTs C	6.829	4.370	2.121	5.916	12.923	100878
	CMTK A	10.556	6.073	3.240	9.747	19.092	112532
	CMTK B	9.697	6.011	2.646	8.718	17.958	53776
	CMTK C	7.610	5.184	2.121	6.364	15.199	65444
	Elastix A	9.033	6.060	2.236	7.616	18.111	199898
	Elastix B	10.309	6.318	2.828	9.301	19.609	100424
Tefor	ANTs A	5.186	5.031	1.225	3.606	11.576	52200
	ANTs B	6.187	4.873	1.581	4.743	13.058	46310
	ANTs C	7.951	5.609	2.000	6.442	16.186	60762
	CMTK A	9.464	6.190	2.121	8.544	18.000	67854
	CMTK B	9.415	6.090	2.236	8.337	18.184	69588
	CMTK C	11.129	6.969	2.915	10.000	21.190	60618
	Elastix A	6.152	5.289	1.581	4.528	13.583	49102
	Elastix B	8.314	6.203	2.000	6.364	17.621	103728

Table S42. LAL_L : lateral accessory lobe

Template	Algorithm	Mean	Std dev	10th perc	median	90th perc	N
JFRC2010	ANTs A	9.178	8.034	1.732	6.519	22.471	19372
	ANTs B	4.415	2.860	1.225	3.873	8.276	29064
	ANTs C	4.149	3.045	1.000	3.464	8.307	47732
	CMTK A	6.167	4.918	1.414	4.950	12.767	55044
	CMTK B	5.427	3.647	1.414	4.690	10.440	48774
	CMTK C	5.454	3.460	1.581	4.975	9.823	40106
	Elastix A	4.994	2.999	1.581	4.528	8.972	16106
	Elastix B	4.680	2.960	1.414	4.243	8.544	22274
JFRC2013	ANTs A	4.549	3.076	1.225	3.873	8.631	23450
	ANTs B	3.661	2.605	1.000	3.082	7.071	43696
	ANTs C	3.624	2.660	1.000	3.000	7.280	42390
	CMTK A	4.543	3.047	1.414	3.873	8.738	24904
	CMTK B	4.474	3.080	1.225	3.808	8.573	25474
	CMTK C	4.436	2.772	1.414	3.873	8.062	40470
	Elastix A	4.353	3.057	1.000	3.674	8.515	26294
	Elastix B	4.620	3.131	1.225	4.000	8.775	24252
JRC2018	ANTs A	4.378	3.041	1.225	3.674	8.485	24512
	ANTs B	4.506	3.037	1.225	3.808	8.515	24614
	ANTs C	4.316	2.850	1.000	3.808	8.093	28654
	CMTK A	4.710	3.125	1.414	4.062	8.888	26174
	CMTK B	4.584	3.055	1.225	3.873	8.746	25826
	CMTK C	4.543	3.108	1.225	3.808	8.746	25846
	Elastix A	4.491	3.149	1.225	3.808	8.631	24382
	Elastix B	4.691	3.151	1.414	4.062	8.746	24012
FCWB	ANTs A	4.290	2.902	1.225	3.606	8.185	24878
	ANTs B	4.211	3.012	1.000	3.536	8.185	27174
	ANTs C	3.897	2.760	1.000	3.317	7.382	28356
	CMTK A	4.296	2.864	1.000	3.808	8.062	73796
	CMTK B	4.522	3.127	1.225	3.873	8.367	39270
	CMTK C	4.203	2.886	1.000	3.606	8.093	45984
	Elastix A	4.496	3.587	1.225	3.808	8.062	47902
	Elastix B	4.921	3.166	1.414	4.301	9.407	50172
Tefor	ANTs A	4.597	3.061	1.414	3.873	8.660	23362
	ANTs B	4.360	2.945	1.414	3.674	8.276	22966
	ANTs C	4.423	2.995	1.000	3.808	8.426	34566
	CMTK A	4.499	2.806	1.414	4.062	8.155	30862
	CMTK B	4.396	2.804	1.414	3.873	8.093	30122
	CMTK C	5.168	3.147	1.581	4.743	9.513	20416
	Elastix A	4.475	2.692	1.414	4.123	8.031	16314
	Elastix B	4.425	2.658	1.414	4.062	7.906	27170

Table S43. CAN_L : cantle

Template	Algorithm	Mean	Std dev	10th perc	median	90th perc	N
JFRC2010	ANTs A	5.702	5.274	1.581	4.472	10.817	199226
	ANTs B	5.715	6.225	1.581	4.301	9.618	165386
	ANTs C	5.753	5.989	1.581	4.528	9.618	187140
	CMTK A	5.132	5.145	1.414	4.062	8.803	218338
	CMTK B	4.996	4.845	1.414	4.062	8.631	218734
	CMTK C	5.457	5.774	1.581	4.183	9.407	200846
	Elastix A	4.937	5.199	1.225	3.808	8.426	189012
	Elastix B	4.852	4.941	1.225	3.808	8.246	200538
JFRC2013	ANTs A	4.993	5.454	1.225	3.808	8.602	178108
	ANTs B	6.124	6.457	1.581	4.528	10.607	144664
	ANTs C	5.968	6.282	1.581	4.359	10.440	150050
	CMTK A	5.528	5.617	1.581	4.243	9.644	173606
	CMTK B	5.683	5.882	1.581	4.359	9.950	164712
	CMTK C	6.089	5.811	1.581	4.637	10.770	145314
	Elastix A	4.909	5.689	1.225	3.606	8.276	192508
	Elastix B	4.845	5.514	1.225	3.606	8.185	198670
JRC2018	ANTs A	4.730	5.167	1.000	3.674	7.937	197440
	ANTs B	5.373	5.741	1.414	4.123	9.301	181890
	ANTs C	5.681	5.736	1.581	4.359	10.050	192824
	CMTK A	4.842	5.356	1.000	3.674	8.185	197642
	CMTK B	4.868	5.361	1.000	3.674	8.307	194334
	CMTK C	4.977	5.309	1.225	3.808	8.515	193026
	Elastix A	4.875	5.211	1.225	3.808	8.185	195568
	Elastix B	5.161	5.565	1.225	3.873	8.860	197934
FCWB	ANTs A	5.020	5.213	1.225	3.808	8.746	191558
	ANTs B	5.986	6.269	1.581	4.528	10.607	145952
	ANTs C	6.040	6.381	1.581	4.528	10.607	155918
	CMTK A	5.365	4.788	1.581	4.359	9.247	192852
	CMTK B	5.442	4.986	1.581	4.359	9.434	193322
	CMTK C	5.592	5.494	1.581	4.243	9.950	173954
	Elastix A	6.366	7.889	1.581	4.583	10.607	194944
	Elastix B	5.446	6.128	1.414	4.123	9.301	198834
Tefor	ANTs A	4.738	5.086	1.225	3.674	7.937	199464
	ANTs B	5.561	6.031	1.581	4.183	9.434	174346
	ANTs C	5.980	6.428	1.581	4.472	10.512	183560
	CMTK A	5.149	5.134	1.225	4.000	9.434	218888
	CMTK B	5.087	5.109	1.225	4.062	9.000	219208
	CMTK C	5.239	5.104	1.581	4.183	8.972	207136
	Elastix A	4.970	5.408	1.225	3.808	8.515	197682
	Elastix B	4.964	4.944	1.414	4.062	8.337	210416

Table S44. AMMC_L : antennal mechanosensory and motor center

Template	Algorithm	Mean	Std dev	10th perc	median	90th perc	N
JFRC2010	ANTs A	4.630	5.270	0.707	3.000	9.874	320720
	ANTs B	3.816	3.557	0.707	2.646	8.573	258504
	ANTs C	4.282	3.797	0.707	3.162	9.460	250196
	CMTK A	5.171	6.012	0.707	2.646	14.577	265902
	CMTK B	4.996	5.497	0.707	2.828	13.134	270070
	CMTK C	5.192	5.249	0.707	3.240	12.610	266652
	Elastix A	3.301	2.947	0.707	2.345	7.314	263970
	Elastix B	3.519	3.570	0.707	2.236	8.185	271836
JFRC2013	ANTs A	2.964	2.740	0.707	2.121	6.519	274140
	ANTs B	3.956	3.560	0.707	2.915	8.775	273296
	ANTs C	3.621	3.400	0.707	2.550	8.093	272964
	CMTK A	3.401	3.014	0.707	2.550	7.211	251372
	CMTK B	3.548	3.139	0.707	2.646	7.517	250490
	CMTK C	4.381	3.392	1.000	3.536	9.000	252590
	Elastix A	3.141	2.831	0.707	2.236	6.856	269952
	Elastix B	3.077	2.832	0.707	2.236	6.708	274714
JRC2018	ANTs A	2.936	2.696	0.707	2.121	6.442	277906
	ANTs B	3.112	2.860	0.707	2.236	6.856	275250
	ANTs C	3.129	2.864	0.707	2.236	6.856	270738
	CMTK A	2.915	2.794	0.707	2.121	6.442	247334
	CMTK B	2.904	2.782	0.707	2.121	6.364	250582
	CMTK C	2.940	2.756	0.707	2.121	6.442	256194
	Elastix A	3.018	2.764	0.707	2.121	6.557	270890
	Elastix B	3.035	2.836	0.707	2.121	6.671	271324
FCWB	ANTs A	3.200	2.942	0.707	2.236	7.106	285442
	ANTs B	3.882	3.600	0.707	2.646	8.660	266108
	ANTs C	3.829	3.367	0.707	2.828	8.573	261238
	CMTK A	5.664	5.290	1.000	3.808	12.923	244344
	CMTK B	5.217	5.143	1.000	3.606	11.424	228770
	CMTK C	4.132	4.121	0.707	2.646	9.670	249120
	Elastix A	7.432	6.613	1.000	5.292	17.132	243274
	Elastix B	4.629	4.765	0.707	2.915	10.998	273922
Tefor	ANTs A	2.932	2.682	0.707	2.121	6.364	274682
	ANTs B	3.530	3.179	0.707	2.550	7.810	263076
	ANTs C	3.692	3.349	0.707	2.646	8.276	259718
	CMTK A	4.028	4.995	0.707	2.236	9.721	250548
	CMTK B	4.129	5.001	0.707	2.236	10.392	254716
	CMTK C	3.852	3.775	0.707	2.646	8.631	251828
	Elastix A	3.176	2.807	0.707	2.236	6.856	270686
	Elastix B	3.731	3.463	0.707	2.646	8.276	266274

Table S45. ICL_L : inferior clamp

Template	Algorithm	Mean	Std dev	10th perc	median	90th perc	N
JFRC2010	ANTs A	6.841	6.256	1.581	5.099	13.379	22424
	ANTs B	4.086	2.934	1.000	3.240	8.307	11390
	ANTs C	4.507	3.029	1.225	3.808	8.944	10434
	CMTK A	6.861	6.411	0.707	4.637	17.117	8222
	CMTK B	7.242	7.921	1.000	4.743	17.641	7780
	CMTK C	8.124	5.609	2.449	7.000	16.509	5620
	Elastix A	4.611	3.085	1.000	4.062	9.110	8760
	Elastix B	5.793	5.397	0.707	3.808	13.675	10622
JFRC2013	ANTs A	3.843	2.835	1.000	3.000	7.937	11872
	ANTs B	5.089	3.287	1.225	4.528	9.670	8524
	ANTs C	4.723	3.322	1.000	3.873	9.618	9558
	CMTK A	3.947	2.867	1.000	3.240	7.906	13272
	CMTK B	4.215	2.919	1.000	3.606	8.276	13264
	CMTK C	4.151	2.845	1.000	3.536	8.093	14454
	Elastix A	4.034	2.971	1.000	3.162	8.544	11234
	Elastix B	4.181	2.877	1.000	3.606	8.276	11774
JRC2018	ANTs A	3.780	2.795	1.000	3.000	7.778	11756
	ANTs B	3.733	2.846	0.707	2.915	7.906	11878
	ANTs C	3.867	2.873	1.000	3.000	8.093	10400
	CMTK A	3.581	2.687	0.707	2.915	7.246	13240
	CMTK B	3.521	2.683	0.707	2.828	7.382	13728
	CMTK C	3.545	2.768	0.707	2.646	7.649	13292
	Elastix A	3.646	2.756	1.000	2.915	7.681	12872
	Elastix B	3.947	2.887	0.707	3.240	8.124	12502
FCWB	ANTs A	3.985	2.916	1.000	3.162	8.093	11384
	ANTs B	5.016	3.673	1.000	4.243	10.149	6992
	ANTs C	4.931	3.364	1.000	4.243	9.849	7812
	CMTK A	4.931	4.047	1.000	3.606	10.886	10626
	CMTK B	5.382	4.323	1.000	4.183	11.726	12736
	CMTK C	4.243	3.157	1.000	3.464	8.944	12936
	Elastix A	6.866	5.075	1.225	5.745	14.089	9312
	Elastix B	7.656	6.481	1.732	6.042	15.925	7320
Tefor	ANTs A	3.797	2.739	1.000	3.000	7.810	12254
	ANTs B	4.168	2.814	1.000	3.536	8.246	10092
	ANTs C	4.115	2.670	1.000	3.536	8.093	12988
	CMTK A	5.519	5.626	0.707	4.031	10.886	12094
	CMTK B	5.515	5.538	0.707	3.808	11.023	11236
	CMTK C	4.592	2.858	1.414	4.062	8.860	14446
	Elastix A	4.895	3.334	1.000	4.243	9.513	9732
	Elastix B	6.338	5.427	1.000	4.528	14.560	8606

Table S46. BU_R : bulb

Template	Algorithm	Mean	Std dev	10th perc	median	90th perc	N
JFRC2010	ANTs A	8.553	6.912	1.581	6.364	18.722	127354
	ANTs B	4.516	3.277	1.225	3.674	8.888	247654
	ANTs C	4.862	3.569	1.225	4.062	9.747	276772
	CMTK A	6.364	5.110	1.414	4.950	13.656	181760
	CMTK B	6.464	5.207	1.414	4.950	14.160	161820
	CMTK C	5.854	4.722	1.414	4.528	12.227	258396
	Elastix A	4.170	3.497	1.000	3.240	8.246	127674
	Elastix B	4.304	3.391	1.000	3.464	8.544	117868
JFRC2013	ANTs A	4.252	3.412	1.000	3.464	8.426	115754
	ANTs B	4.414	3.302	1.225	3.606	8.631	279706
	ANTs C	4.619	3.383	1.225	3.808	9.192	254522
	CMTK A	4.824	3.874	1.000	3.808	9.849	104886
	CMTK B	4.860	3.763	1.225	3.808	9.849	109968
	CMTK C	4.822	3.441	1.414	4.062	9.434	194618
	Elastix A	4.441	3.515	1.000	3.606	8.631	107718
	Elastix B	4.440	3.540	1.000	3.606	8.775	106336
JRC2018	ANTs A	4.268	3.460	1.000	3.464	8.544	114502
	ANTs B	4.623	3.490	1.225	3.808	9.083	141690
	ANTs C	4.590	3.383	1.225	3.808	9.192	145422
	CMTK A	4.504	3.553	1.000	3.606	9.000	106820
	CMTK B	4.472	3.555	1.000	3.606	8.944	101196
	CMTK C	4.553	3.671	1.000	3.606	8.972	103616
	Elastix A	4.296	3.409	1.000	3.536	8.337	113174
	Elastix B	4.257	3.389	1.000	3.464	8.396	112718
FCWB	ANTs A	4.399	3.629	1.000	3.464	8.860	116374
	ANTs B	4.546	3.278	1.414	3.808	8.631	230142
	ANTs C	4.721	3.336	1.414	4.062	8.972	212550
	CMTK A	7.129	5.518	1.581	5.701	15.067	201758
	CMTK B	7.042	5.441	1.581	5.568	14.866	151708
	CMTK C	6.447	5.039	1.581	5.000	13.675	164182
	Elastix A	6.541	5.142	1.581	5.148	13.472	263744
	Elastix B	7.034	5.573	1.581	5.523	14.731	178076
Tefor	ANTs A	4.269	3.440	1.000	3.464	8.485	120644
	ANTs B	4.489	3.298	1.225	3.674	8.803	210210
	ANTs C	5.060	3.693	1.414	4.123	10.075	177482
	CMTK A	7.087	5.766	1.414	5.339	15.906	165070
	CMTK B	7.404	6.048	1.581	5.523	16.643	161920
	CMTK C	6.849	5.351	1.581	5.292	14.731	164558
	Elastix A	4.036	3.284	1.000	3.240	7.906	161986
	Elastix B	4.821	3.675	1.225	3.873	9.618	185564

Table S47. VES_L : vest

Template	Algorithm	Mean	Std dev	10th perc	median	90th perc	N
JFRC2010	ANTs A	4.882	4.210	1.000	3.606	10.583	232736
	ANTs B	4.080	3.540	0.707	3.000	8.718	256620
	ANTs C	4.103	3.172	1.000	3.240	8.544	390228
	CMTK A	4.728	4.691	0.707	3.162	10.583	242754
	CMTK B	4.884	5.420	0.707	3.082	11.269	254096
	CMTK C	4.620	4.982	1.000	3.000	10.512	338184
	Elastix A	2.999	2.482	0.707	2.345	5.874	255814
	Elastix B	3.499	2.962	0.707	2.646	7.280	213680
JFRC2013	ANTs A	3.054	2.715	0.707	2.236	6.442	223036
	ANTs B	4.110	3.312	1.000	3.240	8.396	358766
	ANTs C	4.159	3.331	1.000	3.240	8.944	370482
	CMTK A	3.197	2.739	0.707	2.550	6.442	190126
	CMTK B	3.244	2.740	0.707	2.550	6.557	188414
	CMTK C	4.745	3.770	1.000	3.606	10.440	230108
	Elastix A	2.953	2.545	0.707	2.236	5.831	229984
	Elastix B	3.015	2.666	0.707	2.236	6.083	204902
JRC2018	ANTs A	2.930	2.662	0.707	2.236	6.042	223680
	ANTs B	3.316	3.020	0.707	2.345	7.211	224980
	ANTs C	3.240	2.907	0.707	2.345	7.036	228840
	CMTK A	3.193	2.959	0.707	2.345	6.708	195902
	CMTK B	3.107	2.839	0.707	2.236	6.557	190138
	CMTK C	3.035	2.702	0.707	2.236	6.364	197050
	Elastix A	3.059	2.679	0.707	2.345	6.205	206600
	Elastix B	2.988	2.646	0.707	2.236	6.083	201614
FCWB	ANTs A	3.394	3.016	0.707	2.550	7.280	226180
	ANTs B	4.588	3.574	1.000	3.606	9.618	298536
	ANTs C	3.784	2.918	1.000	3.000	7.746	366938
	CMTK A	6.488	5.469	1.414	4.796	14.107	190292
	CMTK B	5.715	5.134	1.000	4.062	12.767	195356
	CMTK C	4.265	3.892	0.707	3.000	9.539	250136
	Elastix A	7.710	7.839	1.225	5.050	18.083	267584
	Elastix B	4.410	4.285	0.707	3.000	9.618	245972
Tefor	ANTs A	3.053	2.744	0.707	2.236	6.364	224680
	ANTs B	3.920	3.501	0.707	2.915	8.544	228986
	ANTs C	3.954	3.505	0.707	2.915	8.860	252914
	CMTK A	4.384	3.963	0.707	3.000	10.296	206084
	CMTK B	4.337	3.946	0.707	3.000	10.124	208340
	CMTK C	4.332	3.818	0.707	3.162	9.539	203452
	Elastix A	3.112	2.640	0.707	2.449	6.205	228276
	Elastix B	3.587	3.086	0.707	2.646	7.616	206388

Table S48. IB_L : inferior bridge

Template	Algorithm	Mean	Std dev	10th perc	median	90th perc	N
JFRC2010	ANTs A	3.926	2.876	1.000	3.240	7.550	81610
	ANTs B	3.925	2.699	1.000	3.317	7.583	80346
	ANTs C	4.335	2.831	1.225	3.808	8.276	87234
	CMTK A	5.173	5.092	1.000	3.808	9.849	76872
	CMTK B	4.921	4.715	1.000	3.606	9.434	77040
	CMTK C	4.630	3.994	1.000	3.674	8.367	81250
	Elastix A	3.316	2.154	1.000	2.915	6.124	87064
	Elastix B	3.583	2.412	1.000	3.000	7.000	91986
JFRC2013	ANTs A	3.112	2.133	1.000	2.646	5.831	79242
	ANTs B	4.120	2.866	1.000	3.536	8.062	96460
	ANTs C	4.268	3.111	1.000	3.536	8.972	84404
	CMTK A	3.225	2.191	1.000	2.828	6.083	74162
	CMTK B	3.450	2.314	1.000	3.000	6.519	70046
	CMTK C	4.372	2.934	1.225	3.808	8.337	67292
	Elastix A	3.155	2.036	1.000	2.828	5.701	77368
	Elastix B	3.127	2.053	1.000	2.646	5.788	81338
JRC2018	ANTs A	3.006	2.071	0.707	2.550	5.701	80720
	ANTs B	3.131	2.137	1.000	2.646	5.788	74448
	ANTs C	3.297	2.190	1.000	2.915	6.083	74372
	CMTK A	3.208	2.179	1.000	2.828	6.083	79102
	CMTK B	3.173	2.136	1.000	2.646	6.042	78520
	CMTK C	3.158	2.128	1.000	2.646	5.874	77660
	Elastix A	3.248	2.125	1.000	2.915	6.042	76480
	Elastix B	3.083	2.079	1.000	2.646	5.831	83538
FCWB	ANTs A	3.393	2.406	1.000	2.915	6.245	77720
	ANTs B	4.198	2.897	1.225	3.536	8.515	85288
	ANTs C	4.050	2.615	1.225	3.536	7.649	95052
	CMTK A	5.627	5.160	1.414	4.123	11.597	63522
	CMTK B	5.410	5.394	1.225	3.674	11.790	63758
	CMTK C	3.874	2.625	1.000	3.317	7.211	66826
	Elastix A	6.525	7.232	1.414	4.528	11.790	83412
	Elastix B	4.357	3.178	1.000	3.606	8.544	89336
Tefor	ANTs A	3.012	2.067	0.707	2.550	5.701	80992
	ANTs B	3.660	2.400	1.000	3.162	6.708	77408
	ANTs C	3.975	2.649	1.000	3.536	7.550	72348
	CMTK A	3.683	2.532	1.000	3.082	7.211	90296
	CMTK B	3.724	2.548	1.000	3.162	7.314	92468
	CMTK C	3.980	2.613	1.000	3.536	7.382	81148
	Elastix A	3.209	2.125	1.000	2.828	6.042	91852
	Elastix B	3.361	2.214	1.000	2.915	6.442	99390

Table S49. ATL_L : antler

Template	Algorithm	Mean	Std dev	10th perc	median	90th perc	N
JFRC2010	ANTs A	7.843	6.117	1.414	6.364	16.310	239816
	ANTs B	4.882	4.247	1.000	3.606	10.536	169636
	ANTs C	5.226	4.288	1.225	4.062	10.817	145186
	CMTK A	7.478	6.081	1.581	5.745	16.093	112646
	CMTK B	6.826	5.505	1.414	5.339	14.440	115154
	CMTK C	6.944	5.331	1.732	5.568	13.675	145274
	Elastix A	5.228	4.305	1.225	4.062	10.863	138834
	Elastix B	7.382	6.197	1.414	5.339	16.523	114000
JFRC2013	ANTs A	4.344	3.985	1.000	3.082	9.301	156584
	ANTs B	6.904	5.199	1.581	5.523	14.248	130784
	ANTs C	5.645	4.975	1.225	4.123	12.268	149384
	CMTK A	5.374	4.354	1.225	4.123	11.247	139624
	CMTK B	5.432	4.392	1.225	4.183	11.358	142824
	CMTK C	5.417	4.644	1.225	4.123	11.180	178982
	Elastix A	5.023	4.069	1.225	3.873	10.124	141276
	Elastix B	5.548	4.683	1.225	4.183	11.597	129230
JRC2018	ANTs A	4.425	3.963	1.000	3.162	9.487	150742
	ANTs B	4.932	4.289	1.000	3.606	10.700	145352
	ANTs C	5.012	4.227	1.000	3.808	10.583	144126
	CMTK A	4.675	4.011	1.000	3.536	9.849	128454
	CMTK B	4.576	3.993	1.000	3.464	9.644	127400
	CMTK C	4.661	3.978	1.000	3.536	9.706	135654
	Elastix A	4.838	3.958	1.000	3.808	9.899	130144
	Elastix B	5.844	5.171	1.000	4.183	13.077	121474
FCWB	ANTs A	5.445	5.399	1.000	3.536	12.806	154238
	ANTs B	8.573	5.625	2.236	7.416	16.538	96860
	ANTs C	7.817	5.851	1.732	6.519	15.588	124264
	CMTK A	6.758	5.417	1.581	5.099	14.832	115928
	CMTK B	6.119	5.018	1.414	4.528	13.533	124330
	CMTK C	6.238	5.030	1.414	4.637	13.675	142558
	Elastix A	8.272	5.892	1.732	7.036	16.447	117624
	Elastix B	9.648	7.001	2.121	7.937	20.100	94772
Tefor	ANTs A	4.256	3.872	1.000	3.000	9.192	153390
	ANTs B	4.722	4.137	1.000	3.464	10.223	161208
	ANTs C	4.981	4.023	1.000	3.808	10.607	169534
	CMTK A	7.305	5.380	1.581	6.000	15.379	129854
	CMTK B	6.964	5.274	1.581	5.568	14.782	122228
	CMTK C	5.181	4.484	1.000	3.808	11.336	209880
	Elastix A	5.339	4.350	1.225	4.062	11.380	121224
	Elastix B	9.298	7.235	1.732	7.314	19.925	97360

Table S50. CRE_L : crepine

Template	Algorithm	Mean	Std dev	10th perc	median	90th perc	N
JFRC2010	ANTs A	7.145	8.007	1.581	4.743	13.964	91380
	ANTs B	6.229	4.282	1.581	5.292	12.410	60494
	ANTs C	6.416	4.304	1.581	5.523	12.590	58210
	CMTK A	6.655	5.238	1.414	5.050	14.387	60120
	CMTK B	6.406	4.971	1.581	5.000	13.583	81492
	CMTK C	6.463	4.580	1.581	5.339	13.285	74938
	Elastix A	5.762	4.296	1.581	4.528	12.137	62808
	Elastix B	6.287	4.869	1.581	4.950	13.229	61956
JFRC2013	ANTs A	5.118	3.964	1.225	3.873	10.817	67314
	ANTs B	6.594	4.532	1.732	5.568	12.806	54366
	ANTs C	5.766	4.191	1.581	4.743	11.489	54350
	CMTK A	5.550	4.136	1.414	4.359	11.511	68540
	CMTK B	5.647	4.233	1.414	4.528	11.597	69752
	CMTK C	5.630	4.180	1.414	4.583	11.424	58542
	Elastix A	5.424	4.127	1.414	4.243	11.511	64554
	Elastix B	5.225	4.026	1.414	4.062	11.091	65702
JRC2018	ANTs A	5.159	3.957	1.225	4.062	10.724	69302
	ANTs B	5.290	4.014	1.225	4.183	10.909	68308
	ANTs C	5.245	3.992	1.414	4.123	10.817	66266
	CMTK A	5.158	4.013	1.225	3.873	10.886	67844
	CMTK B	5.155	3.989	1.225	3.873	10.863	67512
	CMTK C	5.207	4.011	1.414	4.062	10.977	67636
	Elastix A	5.180	3.918	1.414	4.062	10.817	69618
	Elastix B	5.303	4.103	1.414	4.123	11.358	64394
FCWB	ANTs A	5.690	4.372	1.414	4.528	11.769	56720
	ANTs B	5.220	4.126	1.000	4.062	11.068	55698
	ANTs C	5.146	3.926	1.000	4.123	10.464	59852
	CMTK A	6.471	4.991	1.414	5.099	13.472	51692
	CMTK B	6.402	4.772	1.581	5.196	13.151	52692
	CMTK C	5.146	4.140	1.000	3.873	11.068	63016
	Elastix A	6.927	4.825	1.581	5.874	13.802	62632
	Elastix B	6.015	5.928	1.000	3.873	13.638	57326
Tefor	ANTs A	5.088	3.903	1.225	4.000	10.607	71160
	ANTs B	5.438	3.996	1.414	4.359	11.068	62306
	ANTs C	5.736	4.044	1.581	4.743	11.380	67984
	CMTK A	6.099	5.023	1.414	4.472	13.416	67056
	CMTK B	6.055	4.971	1.414	4.301	13.472	64964
	CMTK C	5.667	4.403	1.414	4.359	12.042	67684
	Elastix A	5.611	4.253	1.414	4.359	11.726	63094
	Elastix B	6.480	5.079	1.581	4.950	13.892	57146

Table S51. MB_PED_L : pedunculus of adult mushroom body

Template	Algorithm	Mean	Std dev	10th perc	median	90th perc	N
JFRC2010	ANTs A	5.895	5.046	1.581	4.528	11.597	135494
	ANTs B	5.647	4.232	1.581	4.583	10.794	93942
	ANTs C	6.059	4.522	1.581	5.000	11.619	98208
	CMTK A	5.182	3.897	1.581	4.301	9.618	124950
	CMTK B	5.238	4.127	1.581	4.243	9.849	121466
	CMTK C	5.962	4.319	1.732	5.050	10.977	88886
	Elastix A	4.942	4.069	1.414	3.873	9.513	122750
	Elastix B	5.055	4.310	1.414	3.873	10.025	131996
JFRC2013	ANTs A	5.296	4.152	1.581	4.359	9.721	111872
	ANTs B	6.160	4.326	1.732	5.196	11.554	93068
	ANTs C	5.942	4.297	1.581	5.000	10.977	101550
	CMTK A	5.691	4.508	1.581	4.528	11.068	115624
	CMTK B	5.785	4.455	1.581	4.743	10.863	108634
	CMTK C	6.098	4.247	1.732	5.196	11.203	95824
	Elastix A	4.934	3.981	1.414	3.873	9.487	130894
	Elastix B	4.890	4.075	1.225	3.808	9.644	133500
JRC2018	ANTs A	4.678	4.021	1.225	3.606	9.000	131582
	ANTs B	5.279	4.156	1.581	4.243	10.000	115318
	ANTs C	5.384	4.236	1.581	4.359	10.464	122964
	CMTK A	4.724	4.148	1.225	3.606	9.247	128888
	CMTK B	4.944	4.139	1.414	3.808	9.434	126064
	CMTK C	5.323	4.164	1.581	4.301	9.950	119020
	Elastix A	4.905	4.078	1.414	3.808	9.460	123552
	Elastix B	4.965	4.286	1.414	3.808	9.849	123732
FCWB	ANTs A	5.525	4.317	1.581	4.472	10.724	111644
	ANTs B	5.282	3.922	1.581	4.359	10.124	79092
	ANTs C	5.348	3.989	1.581	4.472	9.950	89654
	CMTK A	5.570	4.269	1.581	4.528	10.724	102496
	CMTK B	5.517	4.203	1.581	4.472	10.654	97158
	CMTK C	5.266	3.814	1.581	4.359	9.899	80306
	Elastix A	5.428	4.186	1.581	4.472	10.149	100274
	Elastix B	5.563	4.439	1.581	4.359	10.909	112672
Tefor	ANTs A	4.681	3.993	1.225	3.606	9.055	133834
	ANTs B	5.442	4.207	1.581	4.359	10.223	98620
	ANTs C	5.421	4.099	1.581	4.472	10.392	106004
	CMTK A	6.814	6.928	1.414	4.359	17.044	138644
	CMTK B	6.370	5.807	1.581	4.528	14.765	129842
	CMTK C	6.186	4.424	1.732	5.148	11.853	98746
	Elastix A	4.993	4.136	1.414	4.000	9.539	125584
	Elastix B	5.336	4.609	1.414	4.062	10.724	122576

Table S52. MB_VL_L : vertical lobe of adult mushroom body

Template	Algorithm	Mean	Std dev	10th perc	median	90th perc	N
JFRC2010	ANTs A	6.261	6.063	1.225	4.183	14.036	219070
	ANTs B	4.228	3.847	1.000	3.162	8.631	246608
	ANTs C	4.538	3.955	1.000	3.464	9.327	226850
	CMTK A	6.603	6.355	1.414	4.472	15.556	201488
	CMTK B	5.414	4.843	1.000	3.873	11.769	209146
	CMTK C	5.735	5.508	1.225	4.000	12.410	235838
	Elastix A	4.264	3.803	1.000	3.240	8.544	252902
	Elastix B	5.639	5.303	1.000	3.873	12.826	221790
JFRC2013	ANTs A	4.092	3.702	1.000	3.000	8.276	229810
	ANTs B	5.867	4.824	1.581	4.528	12.021	161752
	ANTs C	5.250	4.780	1.000	3.808	10.794	168914
	CMTK A	5.086	4.257	1.225	3.808	10.630	207446
	CMTK B	5.164	4.277	1.225	3.873	10.677	206382
	CMTK C	4.820	4.394	1.000	3.536	10.100	182974
	Elastix A	4.470	3.881	1.000	3.536	8.860	219734
	Elastix B	4.781	4.117	1.000	3.606	9.721	209946
JRC2018	ANTs A	4.045	3.695	1.000	3.000	8.093	239818
	ANTs B	4.313	3.797	1.000	3.240	8.746	224826
	ANTs C	4.363	3.819	1.000	3.317	8.573	224532
	CMTK A	4.353	3.779	1.000	3.317	8.573	223708
	CMTK B	4.317	3.745	1.000	3.317	8.515	219828
	CMTK C	4.439	3.793	1.000	3.464	8.775	217188
	Elastix A	4.363	3.737	1.000	3.464	8.544	230346
	Elastix B	5.155	4.515	1.225	3.808	10.724	211664
FCWB	ANTs A	4.683	4.373	1.000	3.317	10.025	194636
	ANTs B	5.355	4.389	1.414	4.123	11.158	155400
	ANTs C	5.271	4.507	1.225	3.873	10.977	184730
	CMTK A	5.598	4.750	1.414	4.183	11.790	193610
	CMTK B	5.410	4.572	1.414	4.062	11.489	192134
	CMTK C	5.022	4.183	1.225	3.808	10.607	178880
	Elastix A	6.116	4.738	1.581	4.950	12.288	183072
	Elastix B	7.718	7.008	1.581	5.196	18.628	162264
Tefor	ANTs A	4.103	3.678	1.000	3.082	8.185	238708
	ANTs B	4.272	3.742	1.000	3.240	8.746	229654
	ANTs C	4.688	4.236	1.000	3.536	9.618	222946
	CMTK A	5.672	4.650	1.414	4.359	11.937	218430
	CMTK B	5.610	4.568	1.414	4.359	11.597	218734
	CMTK C	5.211	4.507	1.225	3.808	11.180	226658
	Elastix A	4.530	3.883	1.000	3.536	9.220	245986
	Elastix B	8.394	7.716	1.581	5.431	20.445	150204

Table S53. MB_ML_L : medial lobe of adult mushroom body

Template	Algorithm	Mean	Std dev	10th perc	median	90th perc	N
JFRC2010	ANTs A	5.633	6.448	0.707	3.240	15.636	336696
	ANTs B	3.763	3.134	0.707	3.000	7.314	351018
	ANTs C	3.552	3.075	0.707	2.915	7.071	401822
	CMTK A	3.703	3.276	0.707	2.915	7.280	369328
	CMTK B	3.687	3.450	0.707	2.915	7.106	373918
	CMTK C	4.291	3.846	1.000	3.240	8.544	336458
	Elastix A	2.995	2.373	0.707	2.550	5.745	388882
	Elastix B	2.908	2.325	0.707	2.345	5.701	398758
JFRC2013	ANTs A	2.870	2.450	0.707	2.236	5.701	392302
	ANTs B	4.250	3.381	1.000	3.536	8.000	277230
	ANTs C	3.783	3.161	1.000	3.000	7.211	295372
	CMTK A	2.938	2.383	0.707	2.345	5.788	420952
	CMTK B	2.952	2.406	0.707	2.345	5.788	412536
	CMTK C	3.427	2.583	1.000	2.915	6.557	335392
	Elastix A	2.970	2.436	0.707	2.449	5.745	375750
	Elastix B	2.922	2.414	0.707	2.345	5.745	397916
JRC2018	ANTs A	2.814	2.398	0.707	2.236	5.568	397460
	ANTs B	3.253	2.697	0.707	2.646	6.364	381326
	ANTs C	3.358	2.782	0.707	2.646	6.557	385464
	CMTK A	2.841	2.378	0.707	2.236	5.568	441218
	CMTK B	2.820	2.371	0.707	2.236	5.568	421384
	CMTK C	2.869	2.410	0.707	2.345	5.701	403114
	Elastix A	2.966	2.398	0.707	2.449	5.745	391286
	Elastix B	2.990	2.569	0.707	2.345	5.831	386058
FCWB	ANTs A	2.879	2.536	0.707	2.236	5.701	408720
	ANTs B	3.688	2.858	1.000	3.000	7.071	325596
	ANTs C	3.547	3.059	0.707	2.915	6.928	361668
	CMTK A	3.415	2.967	0.707	2.915	6.557	379116
	CMTK B	3.280	3.081	0.707	2.646	6.245	397938
	CMTK C	3.345	2.687	0.707	2.646	6.519	341304
	Elastix A	4.480	4.266	1.000	3.808	8.307	305690
	Elastix B	3.419	2.633	0.707	2.915	6.671	373924
Tefor	ANTs A	2.856	2.475	0.707	2.236	5.701	403824
	ANTs B	3.583	2.955	0.707	2.915	6.856	346276
	ANTs C	3.522	2.991	0.707	2.915	6.708	395204
	CMTK A	3.302	2.756	0.707	2.646	6.671	431620
	CMTK B	3.429	2.851	0.707	2.828	6.856	402012
	CMTK C	3.736	3.057	1.000	3.000	7.106	375272
	Elastix A	3.240	2.631	0.707	2.646	6.124	356548
	Elastix B	3.346	2.919	0.707	2.828	6.403	327064

Table S54. FLA_L : flange

Template	Algorithm	Mean	Std dev	10th perc	median	90th perc	N
JFRC2010	ANTs A	8.631	6.875	2.236	6.856	16.778	362688
	ANTs B	6.772	5.477	2.000	5.523	12.590	338210
	ANTs C	7.036	5.551	2.000	5.701	13.285	401672
	CMTK A	8.762	6.656	2.236	6.964	17.958	409008
	CMTK B	7.881	5.869	2.121	6.364	15.906	383972
	CMTK C	7.383	5.528	2.121	5.916	14.526	433212
	Elastix A	5.820	5.269	1.581	4.359	11.597	335456
	Elastix B	5.722	5.012	1.581	4.359	11.269	352972
JFRC2013	ANTs A	5.921	5.503	1.581	4.528	11.619	398430
	ANTs B	6.585	5.388	1.732	5.196	12.845	388774
	ANTs C	6.848	5.833	1.581	5.292	13.711	393766
	CMTK A	7.142	7.562	1.581	5.000	14.387	424038
	CMTK B	7.345	7.940	1.581	5.099	14.213	422190
	CMTK C	6.931	5.242	2.000	5.701	13.285	434874
	Elastix A	5.742	5.123	1.581	4.359	11.358	427686
	Elastix B	5.650	5.276	1.414	4.183	11.402	438740
JRC2018	ANTs A	5.663	5.194	1.414	4.243	11.336	405342
	ANTs B	5.857	5.177	1.581	4.528	11.402	389376
	ANTs C	6.158	5.304	1.581	4.743	12.166	399826
	CMTK A	5.602	5.111	1.414	4.243	11.068	426888
	CMTK B	5.636	5.213	1.414	4.243	11.180	414846
	CMTK C	5.794	5.173	1.581	4.472	11.336	401586
	Elastix A	5.619	5.051	1.414	4.301	11.091	403720
	Elastix B	5.529	5.317	1.414	4.123	10.724	436688
FCWB	ANTs A	6.237	5.717	1.581	4.690	12.268	398870
	ANTs B	6.452	5.341	1.732	5.000	12.669	336656
	ANTs C	6.778	5.600	1.732	5.339	13.058	359718
	CMTK A	8.018	6.591	2.121	6.364	15.668	388196
	CMTK B	7.788	6.416	2.121	6.083	15.395	365136
	CMTK C	8.460	6.896	2.121	6.442	17.479	344492
	Elastix A	9.498	8.798	2.345	7.416	17.903	384198
	Elastix B	8.059	9.535	1.732	5.568	15.199	405130
Tefor	ANTs A	5.687	5.248	1.414	4.301	11.358	403146
	ANTs B	6.159	5.413	1.581	4.796	11.874	354400
	ANTs C	6.979	5.497	2.000	5.701	13.379	360948
	CMTK A	7.749	6.901	1.581	5.568	16.643	400468
	CMTK B	7.790	6.803	1.581	5.701	16.523	388860
	CMTK C	7.053	5.804	1.732	5.523	14.053	371694
	Elastix A	5.595	5.035	1.414	4.183	11.180	360282
	Elastix B	5.576	5.172	1.414	4.183	11.068	382420

Table S55. LOP_L : lobula plate

Template	Algorithm	Mean	Std dev	10th perc	median	90th perc	N
JFRC2010	ANTs A	5.083	4.229	1.000	3.808	10.977	272138
	ANTs B	4.445	3.253	1.000	3.606	9.083	193424
	ANTs C	4.761	3.300	1.000	4.062	9.539	229120
	CMTK A	5.421	4.942	1.000	3.674	12.767	215634
	CMTK B	4.547	4.221	1.000	3.240	9.874	236674
	CMTK C	4.402	3.624	1.000	3.240	9.592	220518
	Elastix A	3.227	2.491	0.707	2.646	6.481	226828
	Elastix B	3.266	2.638	0.707	2.550	6.671	249262
JFRC2013	ANTs A	3.837	3.072	0.707	3.000	8.185	219702
	ANTs B	4.390	3.105	1.000	3.674	8.746	226656
	ANTs C	4.608	3.175	1.000	3.873	9.110	228424
	CMTK A	3.381	2.529	0.707	2.646	6.964	180538
	CMTK B	3.580	2.653	1.000	2.915	7.246	166454
	CMTK C	4.730	3.301	1.000	4.062	9.460	136042
	Elastix A	3.323	2.621	0.707	2.646	6.819	220190
	Elastix B	3.246	2.504	0.707	2.646	6.557	232012
JRC2018	ANTs A	3.377	2.643	0.707	2.646	6.856	233940
	ANTs B	3.677	2.885	0.707	2.915	7.416	234506
	ANTs C	3.980	3.241	1.000	3.082	8.155	247940
	CMTK A	3.578	2.779	0.707	2.915	7.416	231020
	CMTK B	3.521	2.729	0.707	2.915	7.246	229874
	CMTK C	3.531	2.737	0.707	2.915	7.246	228440
	Elastix A	3.542	2.666	0.707	2.915	7.106	216916
	Elastix B	3.319	2.578	0.707	2.646	6.708	235262
FCWB	ANTs A	4.420	3.391	1.000	3.536	9.220	189324
	ANTs B	5.077	3.406	1.225	4.359	9.849	186316
	ANTs C	5.080	3.429	1.225	4.359	9.874	205584
	CMTK A	5.807	5.115	1.225	4.528	11.358	135822
	CMTK B	5.475	5.004	1.000	4.243	10.724	167728
	CMTK C	4.986	3.759	1.000	4.062	10.536	147642
	Elastix A	6.782	6.050	1.581	5.568	11.874	162078
	Elastix B	4.127	3.456	1.000	3.082	8.888	193698
Tefor	ANTs A	3.341	2.612	0.707	2.646	6.708	228940
	ANTs B	4.523	3.262	1.000	3.808	9.165	176804
	ANTs C	5.212	3.790	1.225	4.301	10.583	195598
	CMTK A	4.028	3.049	1.000	3.240	8.426	215812
	CMTK B	4.164	3.211	1.000	3.317	8.746	207496
	CMTK C	4.652	3.359	1.000	3.808	9.301	169744
	Elastix A	3.223	2.494	0.707	2.646	6.481	221016
	Elastix B	3.029	2.442	0.707	2.345	6.325	256792

Table S56. PB : protocerebral bridge

Template	Algorithm	Mean	Std dev	10th perc	median	90th perc	N
JFRC2010	ANTs A	5.077	5.521	1.225	3.536	10.320	1320934
JFRC2010	ANTs B	3.626	2.491	1.225	3.162	6.364	1378504
JFRC2010	ANTs C	3.687	2.552	1.225	3.240	6.442	1538688
JFRC2010	CMTK A	4.100	3.519	1.225	3.240	7.280	1392898
JFRC2010	CMTK B	3.798	2.808	1.225	3.240	6.708	1415636
JFRC2010	CMTK C	4.126	3.226	1.225	3.464	7.550	1351974
JFRC2010	Elastix A	3.282	2.046	1.000	3.000	5.745	1433024
JFRC2010	Elastix B	3.365	2.045	1.000	3.000	5.916	1500192
JFRC2013	ANTs A	3.265	1.967	1.000	3.000	5.745	1545570
JFRC2013	ANTs B	4.382	3.871	1.225	3.317	8.396	1084472
JFRC2013	ANTs C	4.133	3.487	1.225	3.240	7.649	1202770
JFRC2013	CMTK A	3.513	2.322	1.000	3.082	6.205	1462514
JFRC2013	CMTK B	3.561	2.364	1.000	3.082	6.364	1412544
JFRC2013	CMTK C	3.782	2.740	1.225	3.162	6.856	1126876
JFRC2013	Elastix A	3.266	1.984	1.000	3.000	5.745	1508478
JFRC2013	Elastix B	3.241	1.938	1.000	2.915	5.701	1567854
JRC2018	ANTs A	3.177	1.884	1.000	2.915	5.568	1575740
JRC2018	ANTs B	3.551	2.333	1.000	3.162	6.205	1454164
JRC2018	ANTs C	3.697	2.457	1.225	3.240	6.557	1474432
JRC2018	CMTK A	3.191	1.878	1.000	2.915	5.568	1604044
JRC2018	CMTK B	3.201	1.867	1.000	2.915	5.568	1605078
JRC2018	CMTK C	3.283	1.956	1.000	3.000	5.745	1577672
JRC2018	Elastix A	3.278	1.957	1.000	3.000	5.745	1571160
JRC2018	Elastix B	3.258	1.923	1.000	3.000	5.701	1577384
FCWB	ANTs A	3.610	2.495	1.000	3.082	6.403	1499740
FCWB	ANTs B	4.067	3.255	1.225	3.240	7.517	1022826
FCWB	ANTs C	4.352	3.775	1.225	3.317	8.307	1150918
FCWB	CMTK A	3.710	2.592	1.225	3.240	6.557	1546656
FCWB	CMTK B	3.731	2.659	1.225	3.240	6.557	1535038
FCWB	CMTK C	3.763	2.616	1.225	3.240	6.671	1414018
FCWB	Elastix A	3.745	2.440	1.225	3.317	6.633	1449056
FCWB	Elastix B	3.669	2.483	1.000	3.162	6.557	1464562
Tefor	ANTs A	3.219	1.905	1.000	2.915	5.657	1573310
Tefor	ANTs B	3.530	2.354	1.000	3.082	6.164	1479424
Tefor	ANTs C	3.860	3.124	1.225	3.240	6.708	1554168
Tefor	CMTK A	3.786	2.626	1.000	3.240	6.856	1546958
Tefor	CMTK B	3.754	2.596	1.000	3.240	6.708	1583232
Tefor	CMTK C	4.052	3.224	1.225	3.240	7.416	1560302
Tefor	Elastix A	3.358	2.037	1.000	3.000	5.874	1516002
Tefor	Elastix B	3.752	2.534	1.225	3.240	6.819	1508102

Table S57. AL_L : adult antennal lobe

Template	Algorithm	Mean	Std dev	10th perc	median	90th perc	N
JFRC2010	ANTs A	6.214	5.692	1.581	4.950	11.726	3208676
	ANTs B	5.101	4.599	1.581	4.301	8.860	2887600
	ANTs C	5.413	4.467	1.581	4.583	9.513	3165268
	CMTK A	6.169	5.528	1.581	4.950	11.424	3100936
	CMTK B	5.857	5.420	1.581	4.690	10.607	3023396
	CMTK C	6.269	5.209	1.732	5.148	11.203	2840876
	Elastix A	4.853	4.440	1.581	4.123	8.396	3003908
	Elastix B	4.947	4.383	1.581	4.243	8.602	3015102
JFRC2013	ANTs A	4.898	4.452	1.581	4.123	8.544	3117410
	ANTs B	5.354	4.713	1.581	4.528	9.327	2892702
	ANTs C	5.629	4.817	1.581	4.743	9.950	3018516
	CMTK A	6.786	7.513	1.732	5.000	11.853	2609038
	CMTK B	7.041	8.260	1.732	5.050	11.937	2576206
	CMTK C	6.090	4.471	2.121	5.339	10.464	2663474
	Elastix A	4.892	4.286	1.581	4.183	8.544	3069924
	Elastix B	4.922	4.402	1.581	4.183	8.631	3050764
JRC2018	ANTs A	4.778	4.232	1.581	4.062	8.337	3135938
	ANTs B	5.037	4.500	1.581	4.243	8.775	3102894
	ANTs C	5.550	4.647	1.581	4.583	10.000	3145260
	CMTK A	4.826	4.367	1.581	4.123	8.337	3073602
	CMTK B	4.821	4.426	1.581	4.123	8.307	3085844
	CMTK C	4.912	4.370	1.581	4.183	8.515	3136598
	Elastix A	4.898	4.860	1.581	4.123	8.337	3036246
	Elastix B	4.960	4.595	1.581	4.183	8.544	3024912
FCWB	ANTs A	5.014	4.453	1.581	4.243	8.775	3183810
	ANTs B	5.481	4.718	1.581	4.690	9.513	3063022
	ANTs C	6.039	4.628	2.000	5.196	10.700	3139250
	CMTK A	6.419	5.405	1.732	5.148	12.247	3092300
	CMTK B	6.099	5.175	1.732	5.000	11.247	3118278
	CMTK C	6.466	5.424	2.000	5.339	11.511	2966210
	Elastix A	7.160	7.213	2.121	5.745	13.172	3312420
	Elastix B	6.300	6.954	1.732	5.000	11.424	3294236
Tefor	ANTs A	4.747	4.246	1.414	4.062	8.307	3107898
	ANTs B	5.063	4.669	1.581	4.183	8.860	2912902
	ANTs C	5.747	5.048	1.581	4.743	10.124	3115598
	CMTK A	6.855	7.161	1.581	4.743	14.248	2831230
	CMTK B	6.860	7.124	1.581	4.743	14.300	2839816
	CMTK C	6.573	7.246	1.581	4.743	11.874	2736262
	Elastix A	4.830	4.531	1.581	4.123	8.367	2928176
	Elastix B	4.871	4.529	1.581	4.123	8.515	2906158

Table S58. ME_L : medulla

Template	Algorithm	Mean	Std dev	10th perc	median	90th perc	N
JFRC2010	ANTs A	5.477	6.140	0.707	3.317	13.229	606516
	ANTs B	4.670	4.987	0.707	2.915	11.068	542156
	ANTs C	4.853	5.318	0.707	2.915	11.619	587638
	CMTK A	5.890	6.810	0.707	3.162	15.395	642956
	CMTK B	6.572	7.608	0.707	3.606	17.521	698112
	CMTK C	6.180	6.991	0.707	3.240	16.263	566916
	Elastix A	3.834	4.291	0.707	2.236	9.110	492408
	Elastix B	4.177	4.764	0.707	2.236	10.512	510852
JFRC2013	ANTs A	4.129	4.721	0.707	2.345	10.075	580284
	ANTs B	5.679	5.772	0.707	3.808	13.416	500620
	ANTs C	5.339	5.576	0.707	3.464	12.767	558630
	CMTK A	5.027	4.664	1.000	3.606	10.909	493046
	CMTK B	5.031	4.580	1.000	3.674	10.817	514176
	CMTK C	5.827	5.227	1.000	4.243	12.649	486968
	Elastix A	3.814	4.247	0.707	2.236	9.055	545258
	Elastix B	3.862	4.464	0.707	2.236	9.083	539860
JRC2018	ANTs A	3.767	4.286	0.707	2.121	9.083	568444
	ANTs B	4.289	5.007	0.707	2.236	10.817	565658
	ANTs C	4.532	5.175	0.707	2.646	11.000	601494
	CMTK A	3.818	4.186	0.707	2.236	9.055	573080
	CMTK B	3.792	4.268	0.707	2.236	9.083	560844
	CMTK C	4.090	4.713	0.707	2.236	9.950	575064
	Elastix A	3.712	4.114	0.707	2.236	8.803	533126
	Elastix B	3.859	4.255	0.707	2.236	9.247	543210
FCWB	ANTs A	5.032	5.245	0.707	3.464	11.068	560066
	ANTs B	5.343	5.448	0.707	3.606	12.390	493738
	ANTs C	5.289	5.464	0.707	3.536	12.227	574654
	CMTK A	5.321	5.640	0.707	3.536	12.450	535526
	CMTK B	5.213	5.470	0.707	3.536	12.021	544466
	CMTK C	4.920	5.019	0.707	3.240	11.446	526058
	Elastix A	5.324	5.923	0.707	3.162	13.285	490822
	Elastix B	4.630	5.219	0.707	2.550	11.769	492544
Tefor	ANTs A	3.820	4.392	0.707	2.121	9.220	579900
	ANTs B	4.547	4.824	0.707	2.915	10.770	584556
	ANTs C	4.987	5.100	0.707	3.317	11.424	631596
	CMTK A	5.635	7.627	0.707	2.449	15.906	586094
	CMTK B	5.655	7.379	0.707	2.646	15.182	581586
	CMTK C	5.305	5.935	0.707	3.162	13.058	631288
	Elastix A	3.724	4.277	0.707	2.121	8.972	539944
	Elastix B	4.110	4.671	0.707	2.236	10.247	546230

Table S59. SLP_L : superior lateral protocerebrum

Template	Algorithm	Mean	Std dev	10th perc	median	90th perc	N
JFRC2010	ANTs A	8.202	5.958	2.121	6.964	15.953	157422
	ANTs B	5.008	4.548	1.000	3.674	10.536	99662
	ANTs C	5.419	4.869	1.000	4.000	11.533	100246
	CMTK A	5.625	4.722	1.000	4.359	12.042	93114
	CMTK B	5.574	4.760	1.000	4.243	12.042	91852
	CMTK C	6.369	5.292	1.225	4.796	14.000	89592
	Elastix A	5.266	4.358	1.000	4.123	10.817	99198
	Elastix B	5.711	4.604	1.225	4.528	11.790	100632
JFRC2013	ANTs A	4.915	4.247	1.000	3.808	9.995	99962
	ANTs B	6.260	5.076	1.414	4.950	13.229	101214
	ANTs C	5.382	4.396	1.225	4.243	10.817	105364
	CMTK A	5.675	4.621	1.000	4.472	12.104	110080
	CMTK B	5.762	4.718	1.225	4.528	12.390	109792
	CMTK C	5.857	4.366	1.414	4.950	11.511	105472
	Elastix A	5.131	4.446	1.000	3.873	10.583	100470
	Elastix B	5.238	4.455	1.000	4.062	11.023	99722
JRC2018	ANTs A	4.822	4.152	1.000	3.674	9.849	99134
	ANTs B	5.163	4.570	1.000	3.873	10.817	90884
	ANTs C	5.295	4.634	1.000	4.062	10.886	93564
	CMTK A	4.765	4.110	1.000	3.606	9.670	102790
	CMTK B	4.783	4.211	1.000	3.606	9.849	97670
	CMTK C	4.985	4.311	1.000	3.808	10.320	91042
	Elastix A	4.969	4.155	1.000	3.873	10.050	95666
	Elastix B	5.297	4.474	1.000	4.062	11.247	103016
FCWB	ANTs A	6.747	5.686	1.225	5.099	14.595	108758
	ANTs B	5.041	4.821	1.000	3.606	10.724	101844
	ANTs C	5.002	4.612	1.000	3.606	10.607	115356
	CMTK A	6.305	5.135	1.581	5.000	12.669	78828
	CMTK B	6.121	5.068	1.581	4.796	12.042	80406
	CMTK C	5.460	4.727	1.000	4.183	11.424	108796
	Elastix A	6.421	5.243	1.414	4.950	14.213	100978
	Elastix B	7.532	5.926	1.581	5.831	16.093	108868
Tefor	ANTs A	4.834	4.158	1.000	3.674	9.721	95462
	ANTs B	5.116	4.488	1.000	3.808	10.630	96784
	ANTs C	5.534	4.552	1.414	4.359	11.068	118818
	CMTK A	6.363	5.750	1.000	4.528	15.000	103720
	CMTK B	6.216	5.350	1.225	4.583	13.946	103650
	CMTK C	6.192	5.002	1.414	4.743	13.134	129028
	Elastix A	5.393	4.360	1.000	4.243	11.247	92726
	Elastix B	6.594	5.233	1.225	5.196	14.213	103482

Table S60. SIP_L : superior intermediate protocerebrum

Template	Algorithm	Mean	Std dev	10th perc	median	90th perc	N
JFRC2010	ANTs A	4.487	4.759	0.707	2.915	10.223	2048810
	ANTs B	2.871	2.689	0.707	2.121	6.083	1844162
	ANTs C	2.884	2.818	0.707	2.121	6.124	1905962
	CMTK A	3.977	4.464	0.707	2.550	8.803	1538652
	CMTK B	3.601	3.655	0.707	2.449	8.031	1604384
	CMTK C	4.044	4.257	0.707	2.646	9.220	1457268
	Elastix A	2.834	2.623	0.707	2.121	5.874	1698384
	Elastix B	3.509	3.241	0.707	2.550	7.583	1530020
JFRC2013	ANTs A	2.554	2.523	0.707	1.732	5.431	1978286
	ANTs B	2.862	2.798	0.707	2.121	6.083	1878714
	ANTs C	2.794	2.794	0.707	2.000	6.000	1961782
	CMTK A	2.860	2.840	0.707	2.121	6.083	1808334
	CMTK B	2.973	2.895	0.707	2.121	6.403	1785804
	CMTK C	3.238	2.861	0.707	2.449	6.856	1874888
	Elastix A	2.726	2.593	0.707	2.121	5.568	1745092
	Elastix B	2.828	2.758	0.707	2.121	5.874	1788762
JRC2018	ANTs A	2.564	2.568	0.707	1.732	5.431	1956318
	ANTs B	2.667	2.614	0.707	2.000	5.657	1819568
	ANTs C	2.638	2.641	0.707	2.000	5.568	1914416
	CMTK A	2.546	2.461	0.707	2.000	5.196	1955892
	CMTK B	2.603	2.492	0.707	2.000	5.339	1883916
	CMTK C	2.674	2.576	0.707	2.000	5.568	1853804
	Elastix A	2.734	2.564	0.707	2.121	5.701	1798790
	Elastix B	2.826	2.699	0.707	2.121	5.916	1893642
FCWB	ANTs A	2.785	2.838	0.707	2.000	6.000	1993800
	ANTs B	3.344	2.972	0.707	2.550	6.964	1546890
	ANTs C	3.265	2.969	0.707	2.449	6.856	1817190
	CMTK A	3.279	3.135	0.707	2.345	7.000	1760952
	CMTK B	3.161	3.019	0.707	2.236	6.671	1789962
	CMTK C	3.238	2.987	0.707	2.345	7.000	1673578
	Elastix A	4.141	4.058	0.707	2.915	9.301	1638518
	Elastix B	3.809	3.587	0.707	2.646	8.396	1683704
Tefor	ANTs A	2.536	2.537	0.707	1.732	5.339	1953734
	ANTs B	2.700	2.526	0.707	2.121	5.701	1875044
	ANTs C	2.757	2.620	0.707	2.121	5.831	1996512
	CMTK A	3.524	3.769	0.707	2.236	7.906	1744916
	CMTK B	3.497	3.821	0.707	2.236	7.810	1736936
	CMTK C	3.086	2.971	0.707	2.236	6.557	1797932
	Elastix A	2.843	2.693	0.707	2.121	5.916	1789736
	Elastix B	3.619	3.496	0.707	2.646	7.906	1671190

Table S61. SMP_L : superior medial protocerebrum

Template	Algorithm	Mean	Std dev	10th perc	median	90th perc	N
JFRC2010	ANTs A	7.819	6.241	1.732	5.874	17.393	698640
	ANTs B	5.936	4.431	1.581	5.000	11.511	648902
	ANTs C	6.316	4.712	1.581	5.196	12.410	734882
	CMTK A	6.250	4.859	1.581	5.000	12.669	858926
	CMTK B	6.124	4.779	1.581	4.950	12.369	847098
	CMTK C	6.577	4.771	1.732	5.523	12.903	798272
	Elastix A	5.114	3.961	1.414	4.243	9.618	690276
	Elastix B	5.902	4.761	1.414	4.743	11.790	745100
JFRC2013	ANTs A	4.991	3.977	1.225	4.123	9.487	712030
	ANTs B	6.906	4.970	1.732	5.745	13.583	560778
	ANTs C	6.425	4.972	1.581	5.148	12.826	630090
	CMTK A	5.867	4.372	1.581	4.950	11.424	667434
	CMTK B	5.962	4.403	1.581	5.000	11.597	647920
	CMTK C	6.650	4.636	1.732	5.568	12.903	526864
	Elastix A	5.084	3.939	1.414	4.183	9.618	712258
	Elastix B	5.025	3.841	1.414	4.183	9.426	734544
JRC2018	ANTs A	4.947	3.929	1.225	4.123	9.327	718986
	ANTs B	5.335	4.181	1.414	4.359	10.392	698242
	ANTs C	5.566	4.273	1.581	4.528	10.724	736770
	CMTK A	4.951	3.885	1.225	4.123	9.247	713194
	CMTK B	4.979	3.921	1.414	4.123	9.301	698056
	CMTK C	5.165	4.006	1.414	4.301	9.721	688070
	Elastix A	5.034	3.953	1.414	4.123	9.487	695914
	Elastix B	5.117	3.971	1.414	4.243	9.670	700946
FCWB	ANTs A	5.632	4.534	1.414	4.528	11.000	675074
	ANTs B	6.545	4.684	1.732	5.523	12.826	548688
	ANTs C	6.572	4.857	1.581	5.431	13.153	595164
	CMTK A	6.046	4.456	1.581	5.000	11.705	656072
	CMTK B	5.884	4.447	1.581	4.950	11.380	650172
	CMTK C	6.348	4.550	1.581	5.339	12.268	568660
	Elastix A	6.853	4.846	1.732	5.788	13.153	675556
	Elastix B	6.550	5.625	1.581	5.000	13.675	685246
Tefor	ANTs A	4.977	3.939	1.225	4.123	9.407	732900
	ANTs B	5.710	4.351	1.581	4.690	11.158	671192
	ANTs C	6.294	4.676	1.581	5.196	12.369	718464
	CMTK A	6.738	7.063	1.414	4.637	14.509	742970
	CMTK B	6.745	6.995	1.414	4.637	14.646	728246
	CMTK C	6.572	5.974	1.581	5.000	13.323	669718
	Elastix A	5.089	3.930	1.414	4.243	9.539	714624
	Elastix B	6.053	4.883	1.414	4.743	12.590	781204

Table S62. AVLP_L : anterior ventrolateral protocerebrum

Template	Algorithm	Mean	Std dev	10th perc	median	90th perc	N
JFRC2010	ANTs A	9.112	6.664	2.000	7.416	19.118	187494
	ANTs B	6.464	4.852	1.581	5.292	12.826	147296
	ANTs C	6.652	5.096	1.581	5.523	13.000	152126
	CMTK A	9.757	7.606	1.732	7.583	21.794	202130
	CMTK B	9.154	7.118	1.732	7.141	19.925	197878
	CMTK C	9.658	7.309	2.121	7.517	21.071	231152
	Elastix A	5.831	4.685	1.414	4.528	11.874	158130
	Elastix B	6.175	5.039	1.414	4.743	12.767	153506
JFRC2013	ANTs A	5.290	4.309	1.225	4.123	11.180	160822
	ANTs B	6.855	5.046	1.581	5.745	13.509	146820
	ANTs C	6.684	5.161	1.581	5.385	13.583	147114
	CMTK A	6.048	4.702	1.581	4.743	12.530	170840
	CMTK B	6.044	4.733	1.581	4.743	12.550	167216
	CMTK C	6.499	4.795	1.581	5.339	13.019	145374
	Elastix A	5.297	4.297	1.225	4.123	11.136	160938
	Elastix B	5.363	4.330	1.225	4.183	11.180	159022
JRC2018	ANTs A	5.322	4.354	1.225	4.123	11.180	160018
	ANTs B	5.638	4.674	1.225	4.359	11.874	154582
	ANTs C	5.617	4.473	1.225	4.359	11.597	157128
	CMTK A	5.481	4.422	1.225	4.183	11.597	151870
	CMTK B	5.455	4.455	1.225	4.183	11.597	152622
	CMTK C	5.399	4.402	1.225	4.183	11.269	155220
	Elastix A	5.330	4.344	1.225	4.123	11.068	159874
	Elastix B	5.490	4.411	1.225	4.243	11.489	153876
FCWB	ANTs A	6.041	5.003	1.225	4.528	13.077	157390
	ANTs B	7.107	5.366	1.732	5.745	14.387	156264
	ANTs C	7.099	5.517	1.581	5.701	14.283	154378
	CMTK A	6.887	5.178	1.581	5.745	13.323	152632
	CMTK B	6.696	5.210	1.581	5.523	13.058	151664
	CMTK C	6.804	5.184	1.581	5.431	13.894	152840
	Elastix A	8.175	5.608	2.121	7.000	15.764	177056
	Elastix B	7.828	7.120	1.581	5.701	17.088	152194
Tefor	ANTs A	5.352	4.317	1.225	4.183	11.203	158444
	ANTs B	6.163	4.734	1.581	5.000	12.288	146406
	ANTs C	6.597	5.075	1.581	5.385	13.000	156388
	CMTK A	7.418	6.978	1.414	5.050	17.219	148962
	CMTK B	7.391	6.916	1.414	5.050	17.176	148222
	CMTK C	7.187	6.039	1.581	5.292	16.016	157190
	Elastix A	5.668	4.473	1.414	4.528	11.576	153402
	Elastix B	6.148	4.801	1.581	4.899	12.530	150086

Table S63. PVLP_L : posterior ventrolateral protocerebrum

Template	Algorithm	Mean	Std dev	10th perc	median	90th perc	N
JFRC2010	ANTs A	6.175	4.565	1.732	5.148	11.937	195518
	ANTs B	5.508	3.674	1.732	4.796	9.644	186276
	ANTs C	5.561	3.738	1.732	4.796	9.899	193810
	CMTK A	5.668	3.873	1.581	4.950	10.223	191244
	CMTK B	5.393	3.709	1.581	4.743	9.327	195038
	CMTK C	5.692	3.859	1.732	5.000	10.025	204724
	Elastix A	5.105	3.601	1.581	4.359	9.000	160224
	Elastix B	5.062	3.395	1.581	4.472	8.972	178084
JFRC2013	ANTs A	5.067	3.535	1.581	4.472	8.972	170302
	ANTs B	5.353	3.430	1.732	4.743	9.274	201282
	ANTs C	5.182	3.440	1.581	4.528	9.083	205812
	CMTK A	5.150	3.632	1.581	4.472	9.220	173202
	CMTK B	5.095	3.597	1.581	4.359	9.110	175770
	CMTK C	5.395	3.656	1.732	4.690	9.487	196884
	Elastix A	5.217	3.580	1.581	4.528	9.301	159198
	Elastix B	5.129	3.495	1.581	4.528	9.083	161826
JRC2018	ANTs A	5.046	3.502	1.581	4.359	8.860	168292
	ANTs B	5.154	3.506	1.581	4.528	9.110	176482
	ANTs C	5.174	3.407	1.581	4.528	8.972	189348
	CMTK A	5.116	3.483	1.581	4.528	8.972	164240
	CMTK B	5.130	3.505	1.581	4.528	9.055	161266
	CMTK C	5.164	3.544	1.581	4.528	9.192	164222
	Elastix A	5.095	3.511	1.581	4.472	9.000	157964
	Elastix B	5.048	3.458	1.581	4.359	9.000	168066
FCWB	ANTs A	5.395	3.504	1.732	4.743	9.644	175478
	ANTs B	5.711	3.834	1.732	5.000	10.223	172314
	ANTs C	5.590	3.655	1.732	4.950	9.950	178622
	CMTK A	5.998	4.269	1.732	5.050	11.091	199722
	CMTK B	5.737	4.156	1.732	4.950	10.198	184238
	CMTK C	5.385	3.441	1.732	4.796	9.513	176718
	Elastix A	6.131	4.711	2.000	5.196	10.770	211960
	Elastix B	6.655	6.056	2.000	5.196	12.104	189138
Tefor	ANTs A	5.009	3.438	1.581	4.472	8.718	172432
	ANTs B	5.351	3.486	1.732	4.743	9.301	193554
	ANTs C	5.773	3.943	1.732	5.000	10.223	199872
	CMTK A	5.981	4.548	1.581	5.000	11.269	200804
	CMTK B	6.250	4.823	1.732	5.148	11.832	198428
	CMTK C	5.844	3.845	2.000	5.099	10.512	201972
	Elastix A	5.109	3.341	1.581	4.528	8.972	163504
	Elastix B	5.102	3.337	1.581	4.528	9.110	196782

Table S64. IVLP_L : wedge

Template	Algorithm	Mean	Std dev	10th perc	median	90th perc	N
JFRC2010	ANTs A	6.063	6.078	0.707	3.808	15.067	425196
	ANTs B	4.337	4.460	0.707	2.915	10.075	412812
	ANTs C	4.621	4.692	0.707	3.000	10.440	373646
	CMTK A	6.856	7.376	0.707	3.606	18.111	583892
	CMTK B	5.412	6.127	0.707	2.828	14.595	487112
	CMTK C	7.067	6.968	0.707	4.359	17.734	485036
	Elastix A	3.563	4.048	0.707	2.121	8.746	427644
	Elastix B	3.517	4.245	0.707	2.000	9.000	437410
JFRC2013	ANTs A	3.314	4.099	0.707	1.732	8.093	445348
	ANTs B	4.336	4.214	0.707	3.000	9.539	434152
	ANTs C	4.099	4.118	0.707	2.828	9.110	446134
	CMTK A	3.938	4.224	0.707	2.550	9.110	414136
	CMTK B	4.019	4.284	0.707	2.550	9.247	404460
	CMTK C	4.389	4.287	0.707	3.000	9.849	405546
	Elastix A	3.376	4.025	0.707	2.000	8.155	428920
	Elastix B	3.304	4.045	0.707	1.732	8.031	437122
JRC2018	ANTs A	3.290	4.079	0.707	1.732	8.093	455796
	ANTs B	3.428	4.028	0.707	2.121	8.124	465462
	ANTs C	3.581	4.013	0.707	2.121	8.337	474688
	CMTK A	3.277	4.041	0.707	1.732	8.031	446974
	CMTK B	3.242	4.028	0.707	1.732	7.906	441626
	CMTK C	3.317	4.074	0.707	2.000	7.937	434284
	Elastix A	3.348	4.067	0.707	2.000	8.062	441954
	Elastix B	3.332	4.099	0.707	1.732	8.185	444548
FCWB	ANTs A	3.598	4.510	0.707	2.000	9.055	475416
	ANTs B	4.143	4.617	0.707	2.449	10.000	424340
	ANTs C	4.230	4.600	0.707	2.646	9.925	420268
	CMTK A	5.873	6.766	0.707	3.536	13.964	366682
	CMTK B	5.520	6.419	0.707	3.317	12.903	352618
	CMTK C	4.261	4.896	0.707	2.550	10.512	398244
	Elastix A	6.091	5.778	1.000	4.243	13.946	338514
	Elastix B	5.045	6.085	0.707	2.345	13.656	399758
Tefor	ANTs A	3.298	4.093	0.707	1.732	8.093	464996
	ANTs B	3.830	4.210	0.707	2.345	8.972	452548
	ANTs C	3.920	4.255	0.707	2.449	9.055	482426
	CMTK A	4.351	6.384	0.707	2.000	11.705	443760
	CMTK B	4.445	6.383	0.707	2.121	12.104	442604
	CMTK C	4.642	5.547	0.707	2.646	11.402	446180
	Elastix A	3.475	4.037	0.707	2.121	8.515	443568
	Elastix B	3.436	4.098	0.707	2.000	8.718	442816

Table S65. PLP_L : posterior lateral protocerebrum

Template	Algorithm	Mean	Std dev	10th perc	median	90th perc	N
JFRC2010	ANTs A	16.330	9.444	4.583	15.330	29.283	80554
	ANTs B	8.677	7.334	1.581	6.245	19.609	14514
	ANTs C	8.930	6.973	2.000	7.000	18.788	17056
	CMTK A	12.808	10.303	1.732	10.223	29.034	17914
	CMTK B	13.304	10.055	2.121	11.424	28.783	22412
	CMTK C	13.049	9.556	2.550	10.817	27.295	16882
	Elastix A	8.749	8.171	1.581	5.568	19.929	13058
	Elastix B	8.253	7.852	1.414	5.196	19.925	14726
JFRC2013	ANTs A	8.395	7.372	1.581	5.831	18.173	13596
	ANTs B	8.361	6.597	1.581	6.481	18.014	31014
	ANTs C	7.389	6.613	1.225	5.196	16.872	30028
	CMTK A	12.449	8.885	2.121	11.380	25.159	19976
	CMTK B	12.121	8.747	2.000	10.886	24.546	20068
	CMTK C	9.563	7.412	2.121	7.550	20.591	16956
	Elastix A	8.655	7.742	1.581	5.874	19.248	13438
	Elastix B	8.887	8.003	1.581	6.083	19.975	13216
JRC2018	ANTs A	8.895	8.129	1.581	5.788	20.906	13630
	ANTs B	8.600	8.033	1.581	5.339	20.809	12338
	ANTs C	8.763	7.562	1.581	6.364	19.717	14006
	CMTK A	8.795	7.937	1.581	5.874	19.962	13928
	CMTK B	9.002	8.064	1.581	5.916	20.444	13452
	CMTK C	8.805	7.955	1.581	5.745	20.112	12312
	Elastix A	8.529	7.943	1.581	5.523	19.187	13318
	Elastix B	8.397	7.758	1.581	5.523	19.264	14250
FCWB	ANTs A	11.510	7.480	2.915	10.296	21.920	33530
	ANTs B	6.655	7.305	1.000	3.808	17.960	15710
	ANTs C	6.117	6.348	1.000	4.123	14.560	24774
	CMTK A	9.340	7.220	2.121	7.416	19.352	14154
	CMTK B	9.453	7.078	2.236	7.550	19.506	14176
	CMTK C	7.192	6.623	1.225	5.050	16.643	24994
	Elastix A	8.935	7.286	1.581	7.000	18.577	13950
	Elastix B	8.916	7.954	1.581	6.364	20.106	15566
Tefor	ANTs A	8.627	7.949	1.581	5.523	20.012	14782
	ANTs B	8.462	7.386	1.581	6.042	19.153	16114
	ANTs C	7.989	6.187	1.732	6.364	16.462	31686
	CMTK A	11.916	8.926	1.732	10.817	24.030	18692
	CMTK B	11.288	8.859	1.732	8.860	23.675	19294
	CMTK C	7.860	7.183	1.225	5.523	18.865	30052
	Elastix A	8.494	7.825	1.414	5.568	19.261	13916
	Elastix B	8.488	7.832	1.414	6.000	19.365	16528

Table S66. AOTU_L : anterior optic tubercle

Template	Algorithm	Mean	Std dev	10th perc	median	90th perc	N
JFRC2010	ANTs A	6.263	4.807	1.581	5.000	12.669	306006
	ANTs B	5.442	3.679	1.581	4.690	10.223	269522
	ANTs C	5.904	4.146	1.581	5.000	11.358	304562
	CMTK A	5.675	4.171	1.414	4.690	11.336	295072
	CMTK B	5.607	4.386	1.414	4.528	11.203	282530
	CMTK C	6.292	4.895	1.581	5.000	12.590	258870
	Elastix A	4.858	3.502	1.414	4.123	9.247	286844
	Elastix B	4.880	3.421	1.414	4.183	9.192	305128
JFRC2013	ANTs A	5.114	3.581	1.581	4.359	9.618	318536
	ANTs B	5.922	4.020	1.581	5.099	11.269	259806
	ANTs C	6.058	4.162	1.581	5.148	11.597	314784
	CMTK A	5.545	3.978	1.581	4.528	11.045	351580
	CMTK B	5.460	3.869	1.581	4.528	10.840	350916
	CMTK C	5.422	4.163	1.225	4.472	11.068	340114
	Elastix A	5.051	3.591	1.581	4.243	9.644	283216
	Elastix B	4.972	3.532	1.414	4.183	9.460	322730
JRC2018	ANTs A	4.858	3.416	1.414	4.183	9.165	327132
	ANTs B	5.205	3.477	1.581	4.528	9.644	315620
	ANTs C	5.356	3.547	1.581	4.637	10.050	338732
	CMTK A	4.935	3.424	1.414	4.243	9.247	326348
	CMTK B	4.890	3.416	1.414	4.183	9.192	316248
	CMTK C	4.953	3.447	1.414	4.243	9.301	311878
	Elastix A	4.979	3.523	1.414	4.243	9.460	307772
	Elastix B	5.027	3.514	1.581	4.301	9.434	313066
FCWB	ANTs A	5.352	3.653	1.581	4.583	10.050	346104
	ANTs B	6.272	4.078	2.000	5.431	11.683	267224
	ANTs C	6.367	4.302	1.732	5.431	12.104	293326
	CMTK A	6.098	4.049	1.732	5.196	11.597	289148
	CMTK B	5.860	3.918	1.581	5.000	11.269	297822
	CMTK C	6.310	4.455	1.732	5.292	12.186	278118
	Elastix A	6.531	4.648	1.732	5.431	12.689	276434
	Elastix B	6.760	5.661	1.581	5.148	13.946	303830
Tefor	ANTs A	4.832	3.462	1.225	4.123	9.220	326606
	ANTs B	5.288	3.638	1.581	4.528	9.925	301662
	ANTs C	5.553	3.919	1.581	4.743	10.512	332776
	CMTK A	7.508	7.925	1.414	4.743	19.313	276804
	CMTK B	7.826	8.477	1.414	4.743	21.237	266390
	CMTK C	6.622	6.748	1.581	4.690	13.342	282262
	Elastix A	4.994	3.577	1.414	4.243	9.513	307694
	Elastix B	5.162	3.549	1.581	4.472	9.747	306252

Table S67. LH_R : lateral horn

Template	Algorithm	Mean	Std dev	10th perc	median	90th perc	N
JFRC2010	ANTs A	5.602	4.184	1.414	4.528	11.424	87290
	ANTs B	4.035	3.398	0.707	3.000	8.746	88176
	ANTs C	4.587	3.902	0.707	3.464	10.223	72964
	CMTK A	5.297	5.320	0.707	3.240	12.903	82058
	CMTK B	4.871	5.090	0.707	3.000	11.489	76474
	CMTK C	4.541	4.274	0.707	3.162	10.247	72248
	Elastix A	3.501	2.745	0.707	2.828	7.106	81146
	Elastix B	3.509	2.892	0.707	2.646	7.106	82774
JFRC2013	ANTs A	3.289	2.510	0.707	2.646	6.708	86924
	ANTs B	4.300	3.435	0.707	3.464	9.110	89722
	ANTs C	4.520	3.518	1.000	3.606	9.618	86652
	CMTK A	3.375	2.802	0.707	2.646	7.106	75820
	CMTK B	3.466	2.862	0.707	2.646	7.211	77116
	CMTK C	4.012	3.141	1.000	3.240	7.906	89236
	Elastix A	3.411	2.688	0.707	2.646	7.106	78798
	Elastix B	3.237	2.525	0.707	2.550	6.708	78848
JRC2018	ANTs A	3.295	2.500	0.707	2.646	6.708	87562
	ANTs B	3.520	2.700	0.707	2.915	7.141	87832
	ANTs C	3.407	2.715	0.707	2.646	7.071	80968
	CMTK A	3.346	2.707	0.707	2.646	7.141	71502
	CMTK B	3.344	2.685	0.707	2.646	7.106	73202
	CMTK C	3.420	2.605	0.707	2.828	7.000	84368
	Elastix A	3.347	2.504	0.707	2.646	6.708	93686
	Elastix B	3.283	2.490	0.707	2.646	6.671	86832
FCWB	ANTs A	3.230	2.593	0.707	2.550	6.708	82426
	ANTs B	3.866	3.098	1.000	3.082	7.649	90258
	ANTs C	3.637	2.769	1.000	2.915	7.348	90864
	CMTK A	7.472	7.195	1.225	4.690	19.609	56578
	CMTK B	6.593	6.545	1.225	4.272	16.823	67432
	CMTK C	3.822	3.245	0.707	2.915	8.062	88790
	Elastix A	9.001	8.786	1.000	5.568	24.525	75406
	Elastix B	4.288	3.913	0.707	3.000	9.618	92154
Tefor	ANTs A	3.322	2.569	0.707	2.646	6.856	85508
	ANTs B	3.771	2.933	0.707	3.000	7.810	87320
	ANTs C	3.747	2.940	0.707	3.000	7.842	83340
	CMTK A	4.081	4.368	0.707	2.646	8.860	68066
	CMTK B	4.000	4.067	0.707	2.646	8.631	69704
	CMTK C	3.973	3.401	0.707	3.000	8.093	88896
	Elastix A	3.337	2.629	0.707	2.646	6.745	83424
	Elastix B	3.740	2.994	1.000	2.915	7.906	80286

Table S68. GOR_L : gorget

Template	Algorithm	Mean	Std dev	10th perc	median	90th perc	N
JFRC2010	ANTs A	4.603	3.060	1.414	4.000	8.660	386392
	ANTs B	5.159	3.797	1.414	4.359	9.849	282626
	ANTs C	4.697	3.345	1.225	4.062	8.972	359548
	CMTK A	4.702	3.431	1.225	3.873	9.110	366014
	CMTK B	4.523	3.054	1.225	3.873	8.515	386536
	CMTK C	5.126	3.609	1.414	4.359	9.874	275910
	Elastix A	4.364	2.937	1.225	3.808	8.155	316702
	Elastix B	4.167	2.732	1.225	3.606	7.810	350304
JFRC2013	ANTs A	4.462	2.962	1.225	3.808	8.515	319094
	ANTs B	4.945	3.472	1.414	4.183	9.460	322084
	ANTs C	4.926	3.366	1.414	4.183	9.434	320916
	CMTK A	3.856	2.362	1.225	3.536	7.036	255508
	CMTK B	4.038	2.488	1.225	3.606	7.280	250828
	CMTK C	5.119	3.194	1.581	4.528	9.539	196572
	Elastix A	3.841	2.367	1.000	3.464	7.106	322206
	Elastix B	3.630	2.278	1.000	3.162	6.708	320008
JRC2018	ANTs A	3.620	2.362	1.000	3.082	6.856	393546
	ANTs B	4.268	3.009	1.000	3.606	8.062	369874
	ANTs C	4.068	2.739	1.000	3.536	7.649	394530
	CMTK A	3.851	2.473	1.000	3.317	7.280	413950
	CMTK B	3.913	2.500	1.000	3.464	7.416	401238
	CMTK C	4.106	2.579	1.225	3.606	7.649	382438
	Elastix A	4.150	2.688	1.225	3.606	7.810	376294
	Elastix B	3.766	2.355	1.000	3.317	7.036	382706
FCWB	ANTs A	4.460	3.043	1.225	3.808	8.544	310260
	ANTs B	5.154	3.706	1.414	4.359	9.849	250986
	ANTs C	4.784	3.468	1.225	4.062	9.220	304398
	CMTK A	5.401	4.312	1.225	4.243	11.180	354154
	CMTK B	5.091	3.900	1.225	4.183	10.025	353338
	CMTK C	5.454	4.031	1.414	4.528	10.700	226302
	Elastix A	6.715	5.527	1.225	5.000	15.116	243188
	Elastix B	5.876	5.514	1.225	4.183	13.058	279418
Tefor	ANTs A	3.567	2.322	1.000	3.082	6.745	390314
	ANTs B	4.780	3.532	1.225	4.062	9.083	266852
	ANTs C	4.939	3.731	1.225	4.062	9.721	328196
	CMTK A	5.220	5.363	1.000	3.606	10.416	352240
	CMTK B	5.390	5.499	1.225	3.808	10.863	337534
	CMTK C	5.253	4.117	1.414	4.301	10.000	261764
	Elastix A	3.979	2.581	1.225	3.536	7.382	338726
	Elastix B	3.839	2.465	1.000	3.317	7.106	357760

Table S69. MB_CA_L : calyx of adult mushroom body

Template	Algorithm	Mean	Std dev	10th perc	median	90th perc	N
JFRC2010	ANTs A	7.310	6.162	1.581	5.431	16.279	494082
	ANTs B	5.325	4.211	1.225	4.243	10.817	451332
	ANTs C	5.226	3.977	1.225	4.243	10.440	481422
	CMTK A	6.758	6.100	1.225	4.690	15.556	310332
	CMTK B	6.426	5.861	1.225	4.472	14.748	335610
	CMTK C	6.591	6.309	1.225	4.528	15.524	465200
	Elastix A	4.177	3.341	1.000	3.317	8.276	430482
	Elastix B	4.552	3.815	1.000	3.606	9.083	358604
JFRC2013	ANTs A	4.277	3.570	1.000	3.240	8.775	386086
	ANTs B	5.284	4.153	1.225	4.183	10.794	423082
	ANTs C	5.146	4.120	1.225	4.062	10.512	413470
	CMTK A	4.507	3.717	1.000	3.536	9.055	356370
	CMTK B	4.563	3.702	1.000	3.606	9.192	363436
	CMTK C	5.221	4.367	1.225	4.062	10.654	425034
	Elastix A	4.205	3.479	1.000	3.240	8.515	390606
	Elastix B	4.310	3.698	1.000	3.240	8.660	360798
JRC2018	ANTs A	4.200	3.541	1.000	3.240	8.602	390572
	ANTs B	4.684	3.780	1.000	3.606	9.539	376408
	ANTs C	4.657	3.676	1.000	3.674	9.301	357000
	CMTK A	4.666	3.860	1.000	3.606	9.327	330466
	CMTK B	4.576	3.824	1.000	3.606	9.247	321912
	CMTK C	4.444	3.730	1.000	3.536	8.944	327862
	Elastix A	4.273	3.554	1.000	3.317	8.602	377022
	Elastix B	4.377	3.704	1.000	3.464	8.820	358098
FCWB	ANTs A	4.690	3.962	1.000	3.606	9.618	405374
	ANTs B	5.129	4.091	1.225	4.062	10.392	423360
	ANTs C	4.792	3.673	1.225	3.808	9.592	415376
	CMTK A	6.758	6.919	1.414	4.637	14.646	293282
	CMTK B	6.170	6.604	1.225	4.183	12.903	327190
	CMTK C	5.473	5.267	1.225	3.808	11.511	374896
	Elastix A	6.416	5.949	1.225	4.528	14.526	435286
	Elastix B	5.420	5.013	1.000	3.808	12.042	451186
Tefor	ANTs A	4.215	3.524	1.000	3.240	8.602	397556
	ANTs B	4.961	4.020	1.225	3.873	10.149	424108
	ANTs C	5.222	3.927	1.414	4.301	10.271	393994
	CMTK A	5.359	5.153	1.000	3.808	11.402	340272
	CMTK B	5.429	5.183	1.000	3.808	11.533	340788
	CMTK C	5.132	4.455	1.225	3.873	10.607	447014
	Elastix A	4.159	3.403	1.000	3.240	8.155	417846
	Elastix B	4.672	3.709	1.225	3.674	9.220	359298

Table S70. SPS_L : superior posterior slope

Template	Algorithm	Mean	Std dev	10th perc	median	90th perc	N
JFRC2010	ANTs A	10.205	7.498	2.550	8.485	19.875	209044
	ANTs B	7.295	4.887	2.121	6.205	14.160	183132
	ANTs C	7.084	4.813	2.121	6.000	13.620	193510
	CMTK A	9.097	5.863	2.345	7.937	17.734	167668
	CMTK B	8.030	5.571	2.121	6.856	15.906	166860
	CMTK C	8.026	5.762	2.000	6.442	16.568	213410
	Elastix A	6.012	4.463	1.581	5.000	12.042	192168
	Elastix B	6.456	4.616	1.732	5.339	12.767	192986
JFRC2013	ANTs A	6.601	5.144	1.581	5.196	13.874	188088
	ANTs B	7.082	4.713	2.121	6.083	13.675	176902
	ANTs C	7.015	4.674	2.121	5.916	13.583	186466
	CMTK A	6.739	4.972	1.581	5.568	13.342	169248
	CMTK B	6.843	5.034	1.581	5.701	13.656	165164
	CMTK C	7.357	4.957	2.121	6.164	14.474	189470
	Elastix A	6.205	4.904	1.414	4.796	13.058	181192
	Elastix B	6.383	4.985	1.581	5.050	13.058	180334
JRC2018	ANTs A	6.002	4.824	1.414	4.583	12.369	185274
	ANTs B	7.113	5.055	1.732	5.916	14.071	172736
	ANTs C	7.175	4.979	2.000	6.042	14.089	171534
	CMTK A	6.512	4.920	1.581	5.196	13.285	167674
	CMTK B	6.583	4.995	1.581	5.339	13.416	161066
	CMTK C	6.903	5.237	1.581	5.568	14.053	158340
	Elastix A	6.178	4.929	1.414	4.796	12.806	184982
	Elastix B	6.339	4.904	1.581	5.050	13.019	176462
FCWB	ANTs A	6.598	4.791	1.581	5.431	13.435	197364
	ANTs B	6.999	4.749	2.000	6.042	13.304	162610
	ANTs C	6.932	4.802	2.000	5.874	13.435	157050
	CMTK A	9.012	6.139	2.236	7.778	17.607	247140
	CMTK B	8.812	6.140	2.236	7.416	17.364	216198
	CMTK C	7.849	5.540	2.000	6.442	16.016	189734
	Elastix A	8.489	6.017	2.121	7.106	16.643	272580
	Elastix B	8.059	6.710	1.581	6.205	16.778	286162
Tefor	ANTs A	6.097	4.774	1.414	4.796	12.669	184588
	ANTs B	7.181	4.730	2.121	6.205	13.820	185350
	ANTs C	7.416	4.873	2.236	6.364	14.248	173904
	CMTK A	7.824	5.968	1.732	6.364	16.000	207344
	CMTK B	8.036	6.168	1.732	6.481	16.583	199956
	CMTK C	7.281	5.378	1.732	6.083	14.248	219074
	Elastix A	5.726	4.444	1.414	4.528	11.597	200776
	Elastix B	6.505	4.685	1.581	5.385	12.767	197976

Table S71. IPS_L : inferior posterior slope

Template	Algorithm	Mean	Std dev	10th perc	median	90th perc	N
JFRC2010	ANTs A	5.455	6.304	0.707	3.162	13.285	365908
	ANTs B	3.064	3.233	0.707	2.000	7.314	310344
	ANTs C	3.262	3.507	0.707	2.000	8.062	287574
	CMTK A	3.271	4.042	0.707	1.732	8.660	316734
	CMTK B	4.493	5.100	0.707	2.236	12.288	378216
	CMTK C	4.372	4.872	0.707	2.345	11.597	354038
	Elastix A	2.718	2.975	0.707	1.581	6.403	313594
	Elastix B	2.957	3.658	0.707	1.581	7.036	302568
JFRC2013	ANTs A	2.501	2.654	0.707	1.581	5.831	330144
	ANTs B	3.351	3.483	0.707	2.121	8.124	306500
	ANTs C	3.029	3.191	0.707	1.732	7.246	325802
	CMTK A	3.161	2.930	0.707	2.236	6.964	308818
	CMTK B	3.173	2.976	0.707	2.236	7.000	309956
	CMTK C	3.734	3.275	0.707	2.828	7.937	296110
	Elastix A	2.585	2.750	0.707	1.581	6.083	327732
	Elastix B	2.587	2.758	0.707	1.581	6.083	324458
JRC2018	ANTs A	2.486	2.641	0.707	1.581	5.788	333308
	ANTs B	2.673	2.819	0.707	1.581	6.364	328600
	ANTs C	2.700	2.774	0.707	1.732	6.403	328444
	CMTK A	2.446	2.604	0.707	1.581	5.701	326512
	CMTK B	2.434	2.604	0.707	1.581	5.701	326858
	CMTK C	2.493	2.660	0.707	1.581	5.745	323692
	Elastix A	2.561	2.688	0.707	1.581	5.916	328392
	Elastix B	2.569	2.845	0.707	1.581	6.083	332010
FCWB	ANTs A	3.331	3.599	0.707	2.000	8.185	319986
	ANTs B	3.417	3.764	0.707	2.121	8.337	291926
	ANTs C	3.354	3.612	0.707	2.121	8.246	304426
	CMTK A	3.989	4.814	0.707	2.236	9.849	252984
	CMTK B	3.966	4.561	0.707	2.345	9.618	258242
	CMTK C	3.019	3.575	0.707	1.732	7.106	311560
	Elastix A	4.944	5.453	0.707	2.915	12.530	261840
	Elastix B	3.853	5.500	0.707	1.732	9.721	278840
Tefor	ANTs A	2.467	2.608	0.707	1.581	5.745	332778
	ANTs B	2.821	2.956	0.707	1.732	6.671	318136
	ANTs C	3.077	3.168	0.707	2.000	7.246	337976
	CMTK A	2.942	4.012	0.707	1.581	6.964	320210
	CMTK B	2.960	4.025	0.707	1.581	7.000	317748
	CMTK C	3.010	3.521	0.707	1.732	6.856	323594
	Elastix A	2.625	2.881	0.707	1.581	6.124	316456
	Elastix B	3.013	3.655	0.707	1.732	7.246	297784

Table S72. SCL_L : superior clamp

Template	Algorithm	Mean	Std dev	10th perc	median	90th perc	N
JFRC2010	ANTs A	8.473	6.129	1.732	6.856	17.264	20800
	ANTs B	9.971	8.119	1.732	7.649	23.592	5310
	ANTs C	13.086	9.894	2.646	10.025	28.267	5350
	CMTK A	8.004	7.301	1.225	4.743	19.416	7936
	CMTK B	8.871	7.742	1.414	5.701	20.541	8882
	CMTK C	14.072	8.653	3.162	13.229	26.086	8348
	Elastix A	5.027	3.985	1.000	3.873	11.091	8620
	Elastix B	6.502	5.606	1.225	4.528	14.782	11278
JFRC2013	ANTs A	4.772	3.640	1.000	3.674	9.925	11272
	ANTs B	9.053	6.853	2.345	6.856	19.912	8706
	ANTs C	9.880	7.181	2.121	7.842	20.628	8176
	CMTK A	4.652	3.545	1.000	3.606	9.849	7850
	CMTK B	4.737	3.664	1.000	3.674	9.849	7898
	CMTK C	5.578	5.375	1.000	3.808	11.864	8396
	Elastix A	4.517	3.482	1.000	3.536	9.327	11440
	Elastix B	4.331	3.306	1.000	3.536	8.746	10200
JRC2018	ANTs A	4.715	3.604	1.000	3.674	9.872	11212
	ANTs B	5.370	4.386	1.000	4.123	11.597	10352
	ANTs C	6.863	5.464	1.581	5.431	12.923	9650
	CMTK A	4.177	3.202	1.000	3.240	9.000	7242
	CMTK B	4.271	3.175	1.000	3.464	8.964	8104
	CMTK C	4.366	3.299	1.000	3.536	8.972	9412
	Elastix A	4.523	3.391	1.000	3.606	9.083	10326
	Elastix B	4.229	3.177	1.000	3.536	8.515	9400
FCWB	ANTs A	5.231	4.080	1.225	4.123	11.203	10960
	ANTs B	6.989	5.387	1.732	5.523	14.232	11730
	ANTs C	7.271	6.425	1.581	5.000	17.116	10192
	CMTK A	6.679	6.664	1.581	4.528	14.179	5850
	CMTK B	7.742	6.833	1.732	5.745	16.233	7096
	CMTK C	5.272	5.928	1.000	3.536	10.392	7818
	Elastix A	12.588	6.992	2.646	13.304	21.389	11342
	Elastix B	10.268	6.293	2.121	9.874	18.557	14104
Tefor	ANTs A	4.496	3.439	1.000	3.536	9.434	10886
	ANTs B	7.671	6.469	1.581	5.523	17.769	8006
	ANTs C	8.403	7.111	1.581	5.874	19.313	6976
	CMTK A	5.387	6.922	1.000	3.000	11.402	5952
	CMTK B	5.379	6.771	1.000	3.162	11.834	6314
	CMTK C	7.147	6.188	1.414	5.523	15.484	10986
	Elastix A	4.854	4.112	1.000	3.606	9.925	8444
	Elastix B	6.968	5.819	1.581	5.148	15.133	10642

Table S73. EPA_L : epaulette

Template	Algorithm	Mean	Std dev	10th perc	median	90th perc	N
JFRC2010	ANTs A	9.792	6.880	2.915	8.276	19.300	6906
	ANTs B	7.664	3.981	2.646	7.141	13.304	5612
	ANTs C	8.216	4.275	3.000	7.665	14.027	6166
	CMTK A	8.411	4.059	3.082	8.276	13.675	4720
	CMTK B	8.171	4.245	2.540	7.842	13.946	7860
	CMTK C	8.291	4.460	2.646	8.031	14.370	7384
	Elastix A	7.822	4.291	2.236	7.348	14.053	5158
	Elastix B	7.306	3.983	2.121	7.036	12.669	4932
JFRC2013	ANTs A	7.327	3.693	2.646	7.000	12.510	4904
	ANTs B	8.966	4.455	3.464	8.544	15.149	3702
	ANTs C	7.549	4.321	2.121	7.071	13.952	3488
	CMTK A	8.723	4.345	3.162	8.515	14.474	5628
	CMTK B	8.507	4.346	3.000	8.155	14.474	6310
	CMTK C	8.175	4.181	3.162	7.416	13.964	4600
	Elastix A	8.221	3.821	3.240	8.062	13.435	4282
	Elastix B	7.716	4.024	2.550	7.314	13.388	5236
JRC2018	ANTs A	7.185	3.763	2.345	6.708	12.530	5298
	ANTs B	7.432	3.864	2.449	7.106	12.826	5168
	ANTs C	7.357	3.788	2.646	6.856	12.708	6022
	CMTK A	7.320	3.819	2.550	6.856	12.767	4962
	CMTK B	7.285	3.863	2.345	6.856	12.927	4570
	CMTK C	7.396	4.024	2.236	7.106	13.077	5154
	Elastix A	7.352	3.767	2.345	7.141	12.530	5522
	Elastix B	7.662	4.076	2.345	7.246	13.416	4790
FCWB	ANTs A	7.724	3.923	2.646	7.382	13.153	4658
	ANTs B	7.151	4.437	2.000	6.481	13.058	5524
	ANTs C	7.915	3.768	3.240	7.649	13.058	5910
	CMTK A	7.525	3.861	2.646	7.211	13.088	5108
	CMTK B	7.678	3.944	2.915	7.246	13.416	4854
	CMTK C	8.755	3.762	4.183	8.276	14.230	3826
	Elastix A	8.976	4.336	3.606	8.544	14.612	5108
	Elastix B	7.824	3.881	2.646	7.616	13.077	5912
Tefor	ANTs A	7.285	3.799	2.449	6.856	12.765	5312
	ANTs B	7.383	3.776	2.737	7.000	12.610	5956
	ANTs C	8.357	4.225	3.041	8.031	13.964	6816
	CMTK A	8.646	4.419	3.082	8.185	14.577	4492
	CMTK B	9.250	4.824	3.162	9.301	15.182	3766
	CMTK C	9.734	4.802	3.536	9.301	16.553	4354
	Elastix A	7.722	4.249	2.000	7.416	13.583	4586
	Elastix B	7.232	3.787	2.345	7.036	12.300	5808

Table S74. GA_L : gall

Template	Algorithm	Mean	Std dev	10th perc	median	90th perc	N
JFRC2010	ANTs A	11.570	7.574	2.345	10.464	22.113	176308
	ANTs B	5.994	5.228	1.414	4.528	12.767	53780
	ANTs C	6.006	5.241	1.414	4.528	12.669	48400
	CMTK A	9.958	6.654	2.345	8.660	19.672	95702
	CMTK B	10.801	7.191	2.345	9.539	21.378	84010
	CMTK C	8.467	5.685	2.121	7.246	16.748	102936
	Elastix A	6.023	5.398	1.414	4.528	12.689	48958
	Elastix B	6.049	4.992	1.414	4.743	12.042	56630
JFRC2013	ANTs A	4.988	5.337	1.000	3.162	11.790	47072
	ANTs B	5.080	3.931	1.414	4.000	10.320	158968
	ANTs C	5.849	4.347	1.581	4.690	11.705	130506
	CMTK A	6.402	5.851	1.414	4.528	14.018	37398
	CMTK B	6.525	5.589	1.581	4.950	13.435	41744
	CMTK C	6.529	4.821	1.732	5.292	12.923	82804
	Elastix A	5.209	5.190	1.000	3.606	11.597	51690
	Elastix B	5.227	5.188	1.000	3.606	11.597	50770
JRC2018	ANTs A	5.152	5.552	1.000	3.240	12.186	46336
	ANTs B	5.764	4.816	1.225	4.528	11.604	53168
	ANTs C	5.795	4.116	1.581	4.950	10.724	75474
	CMTK A	5.810	5.894	1.000	3.808	13.730	33196
	CMTK B	5.847	5.896	1.000	3.873	13.739	32616
	CMTK C	5.934	5.925	1.225	4.062	13.647	35616
	Elastix A	5.765	5.560	1.000	4.062	13.058	41278
	Elastix B	5.981	5.689	1.225	4.123	13.675	37368
FCWB	ANTs A	6.636	5.688	1.225	4.796	14.916	50220
	ANTs B	5.839	4.106	1.581	4.899	11.402	133492
	ANTs C	6.722	4.678	1.732	5.701	13.285	105324
	CMTK A	10.190	6.568	2.345	9.192	19.975	113524
	CMTK B	9.805	6.403	2.345	8.602	18.947	71940
	CMTK C	7.424	5.566	1.732	6.000	15.572	74432
	Elastix A	9.369	6.192	2.236	8.246	18.453	186822
	Elastix B	11.057	7.018	2.449	10.149	21.225	128624
Tefor	ANTs A	5.094	5.423	1.000	3.317	12.042	46480
	ANTs B	5.715	4.603	1.414	4.528	11.790	46570
	ANTs C	7.075	5.221	1.732	5.701	13.874	61358
	CMTK A	10.621	6.530	2.550	9.849	19.698	80892
	CMTK B	10.684	6.664	2.550	9.823	19.975	77374
	CMTK C	10.544	7.218	2.236	9.301	20.881	81748
	Elastix A	6.861	5.579	1.581	5.431	14.177	41516
	Elastix B	8.750	5.938	2.121	7.416	17.507	103946

Table S75. LAL_R : lateral accessory lobe

Template	Algorithm	Mean	Std dev	10th perc	median	90th perc	N
JFRC2010	ANTs A	5.974	5.347	1.581	4.528	11.619	815870
	ANTs B	4.852	3.709	1.581	4.123	8.631	856040
	ANTs C	4.952	3.894	1.581	4.183	8.860	901644
	CMTK A	5.157	4.126	1.581	4.183	9.618	867552
	CMTK B	5.005	3.960	1.581	4.123	9.165	846594
	CMTK C	5.125	4.054	1.581	4.243	9.192	846840
	Elastix A	4.538	3.565	1.414	3.808	8.185	750648
	Elastix B	4.755	3.790	1.414	4.000	8.573	723218
JFRC2013	ANTs A	4.608	3.762	1.225	3.808	8.515	796100
	ANTs B	5.149	4.085	1.581	4.359	9.192	790572
	ANTs C	5.098	4.178	1.581	4.243	9.192	820998
	CMTK A	4.904	3.861	1.414	4.123	9.055	746112
	CMTK B	4.907	3.813	1.414	4.123	9.000	741396
	CMTK C	5.122	3.787	1.581	4.359	9.247	766278
	Elastix A	4.569	3.627	1.225	3.808	8.307	717052
	Elastix B	4.614	3.717	1.225	3.808	8.396	734362
JRC2018	ANTs A	4.567	3.849	1.225	3.808	8.276	757694
	ANTs B	4.892	3.770	1.581	4.123	8.775	783234
	ANTs C	5.052	3.894	1.581	4.243	9.083	815800
	CMTK A	4.598	3.879	1.225	3.808	8.396	751242
	CMTK B	4.568	3.770	1.225	3.808	8.367	739994
	CMTK C	4.631	3.699	1.414	3.808	8.396	733568
	Elastix A	4.553	3.665	1.225	3.808	8.276	720306
	Elastix B	4.719	3.895	1.414	3.873	8.544	725992
FCWB	ANTs A	4.685	3.689	1.225	3.873	8.544	751334
	ANTs B	4.765	3.932	1.414	4.000	8.544	846026
	ANTs C	4.749	3.804	1.414	4.000	8.515	855024
	CMTK A	5.086	3.868	1.581	4.301	9.165	831892
	CMTK B	4.993	3.845	1.581	4.243	8.972	819294
	CMTK C	4.922	3.642	1.581	4.183	8.746	781292
	Elastix A	5.654	5.084	1.581	4.583	10.100	802456
	Elastix B	5.328	4.575	1.581	4.472	9.618	872146
Tefor	ANTs A	4.574	3.664	1.225	3.808	8.276	756640
	ANTs B	5.023	3.748	1.581	4.301	9.000	811812
	ANTs C	5.192	3.922	1.581	4.359	9.407	828668
	CMTK A	5.294	4.177	1.414	4.359	10.075	769982
	CMTK B	5.270	4.061	1.414	4.359	10.075	761656
	CMTK C	5.243	4.073	1.581	4.359	9.460	783740
	Elastix A	4.763	3.676	1.414	4.062	8.544	710380
	Elastix B	5.181	3.827	1.581	4.472	9.407	750662

Table S76. SAD : saddle

2 Qualitative registration results

Finally, we present a more thorough qualitative comparison of registration results. We first randomly selected two of our evaluation images, then display three z-slices of those nc82 images after registration to a particular template, using a particular algorithm. This is repeated for every template-algorithm pair, with the same two moving images. The z-slices were chosen to show similar anatomical structures, and therefore vary across templates.

Arrows were placed once on each template image at similar (but not identical) locations, and are displayed at the same location in each warped image. Similar anatomical structure around each arrow for both images indicates that registration is consistent from image to image. These anatomical structures include the: mushroom body, ellipsoid body, lateral horn, protocerebral bridge, and the anterior optic tubercle. Large differences in structure at the same location in the two aligned images is an indication of poor registration accuracy, but good apparent alignment could still result from inadequate registration. See the discussion limitations of using manually chosen landmark points in Sections 4.2.1 and 5.1.

Despite the limitations of this qualitative assessment, we will briefly point out some observations given the examples below. For the CMTK registrations using JRC 2018F, the ellipsoid body appears overly deformed using CMTK A (Fig. S4) and CMTK C (Fig. S5), but not CMTK B (Fig. S5). This is consistent with the JSD. Some registrations produced unusable results, with significant scaling issues, see Tefor-CMTK B (Fig. S37). Over-warping is evident using ANTs C, for example, with JFRC 2013 (Fig. S19). Surprisingly, some elastix transformations over-warp as well despite generally low-values of JSD, for example JFRC 2010 and Elastix A (Fig. S31). It could be that elastix does not over-warp on average, but happened to produce a poor result for one of the images randomly selected here.

In general, there exists an algorithm that works well for every template. Overall, registration quality seems most consistent when using the JRC 2018 template.

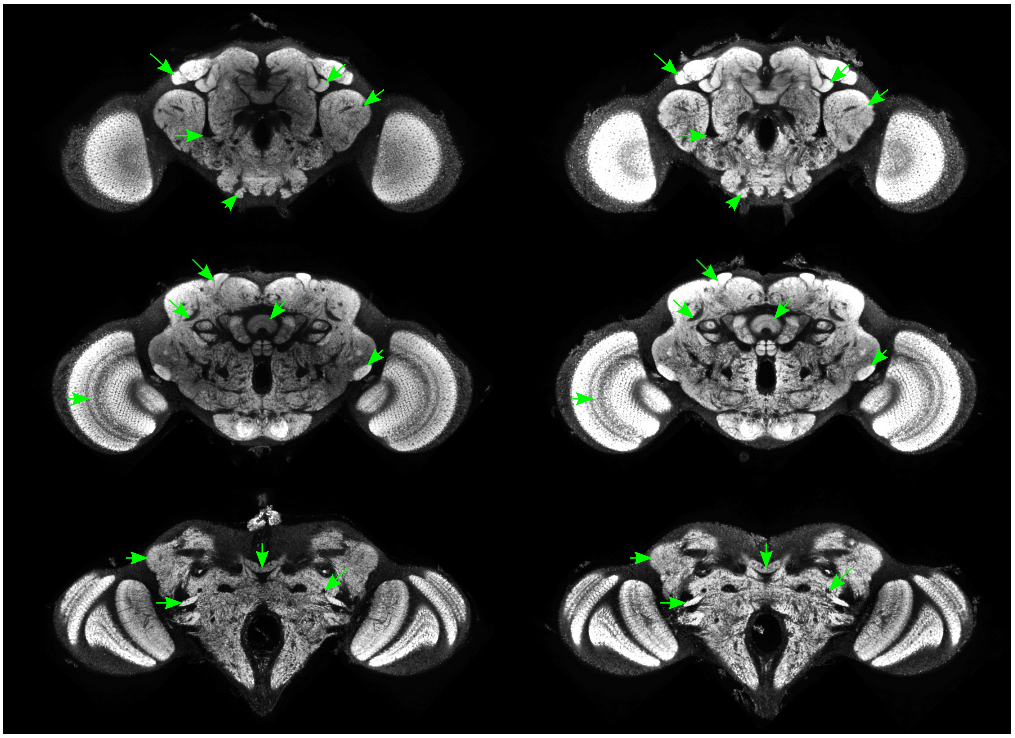

Fig S1. JRC2018 antsA

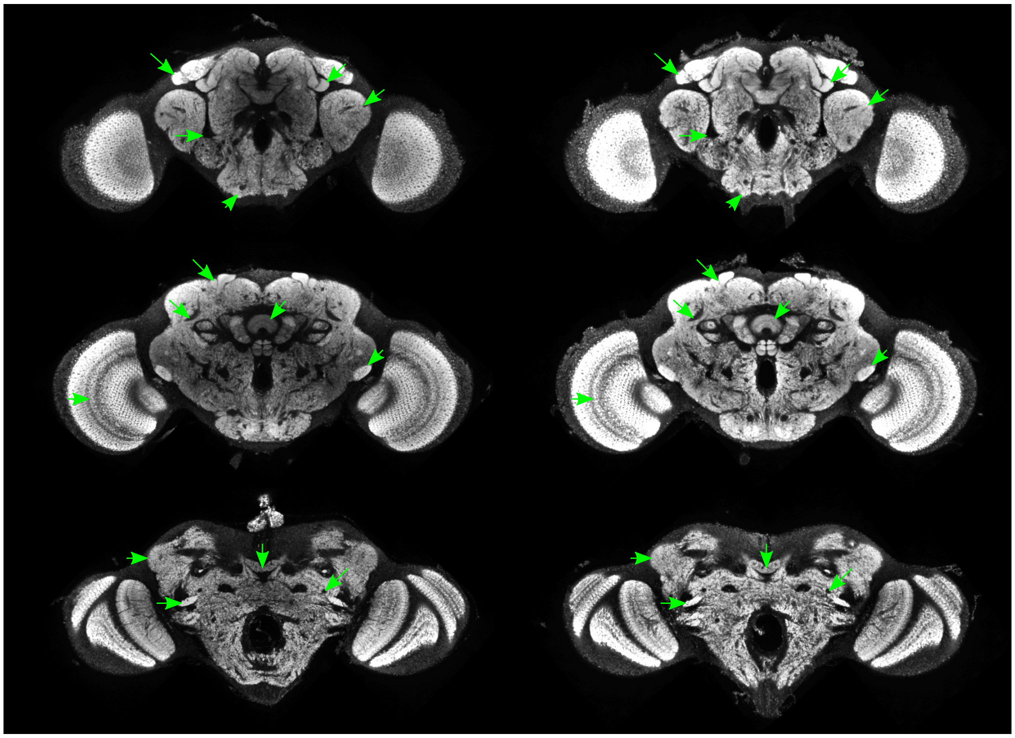

Fig S2. JRC2018 antsB

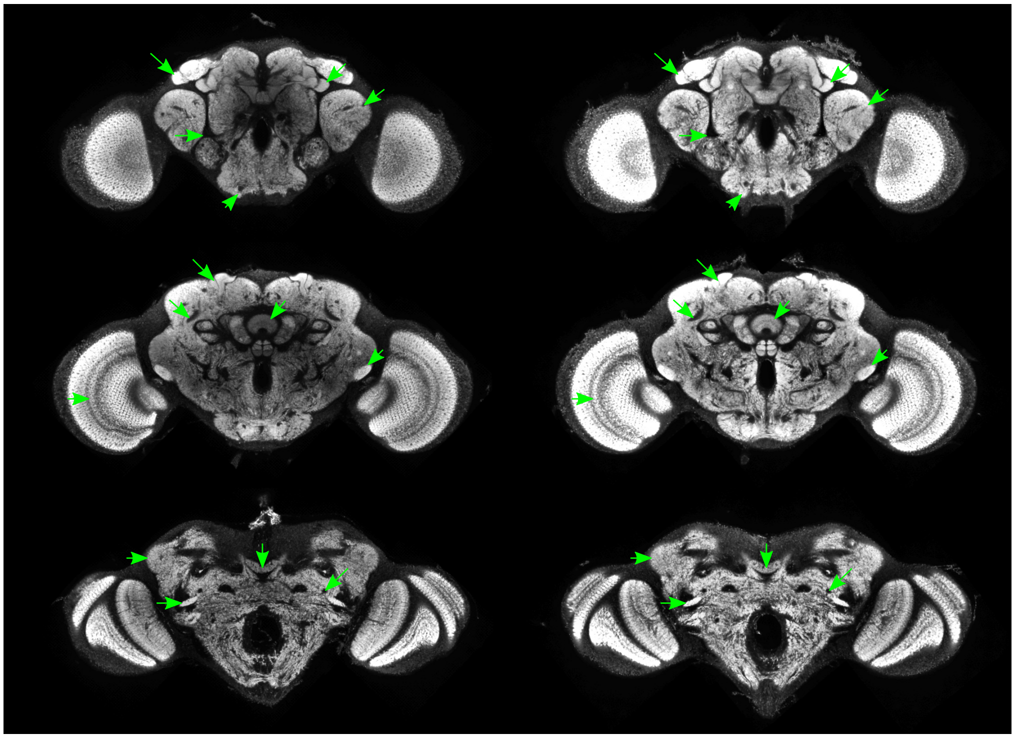

Fig S3. JRC2018 antsC

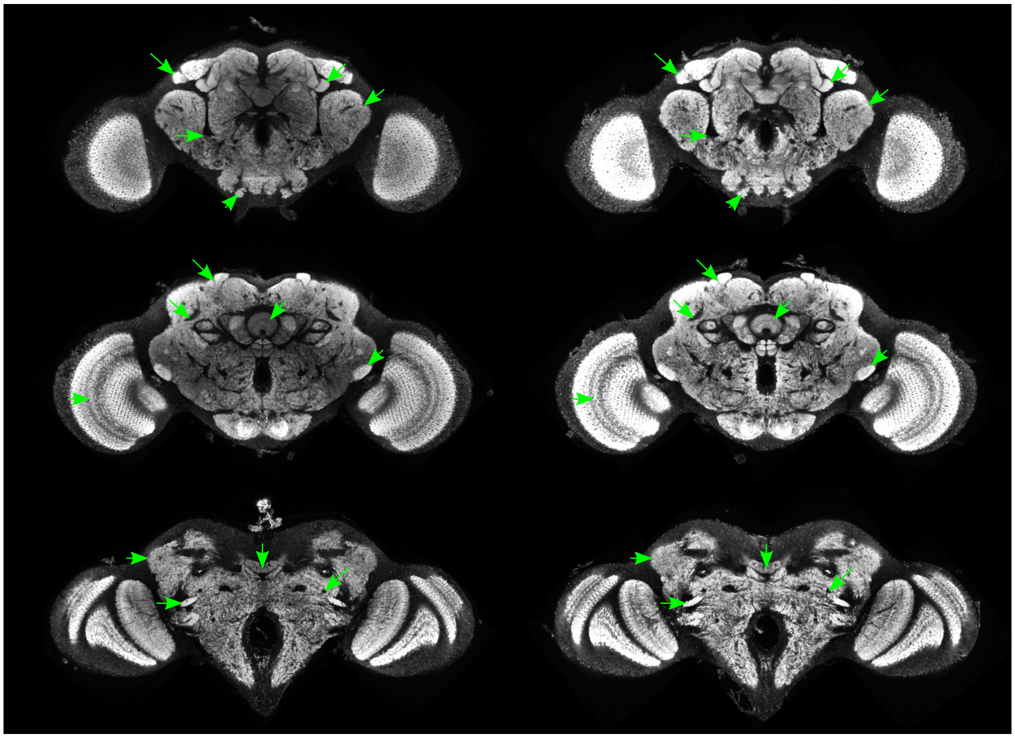

Fig S4. JRC2018 cmtkA

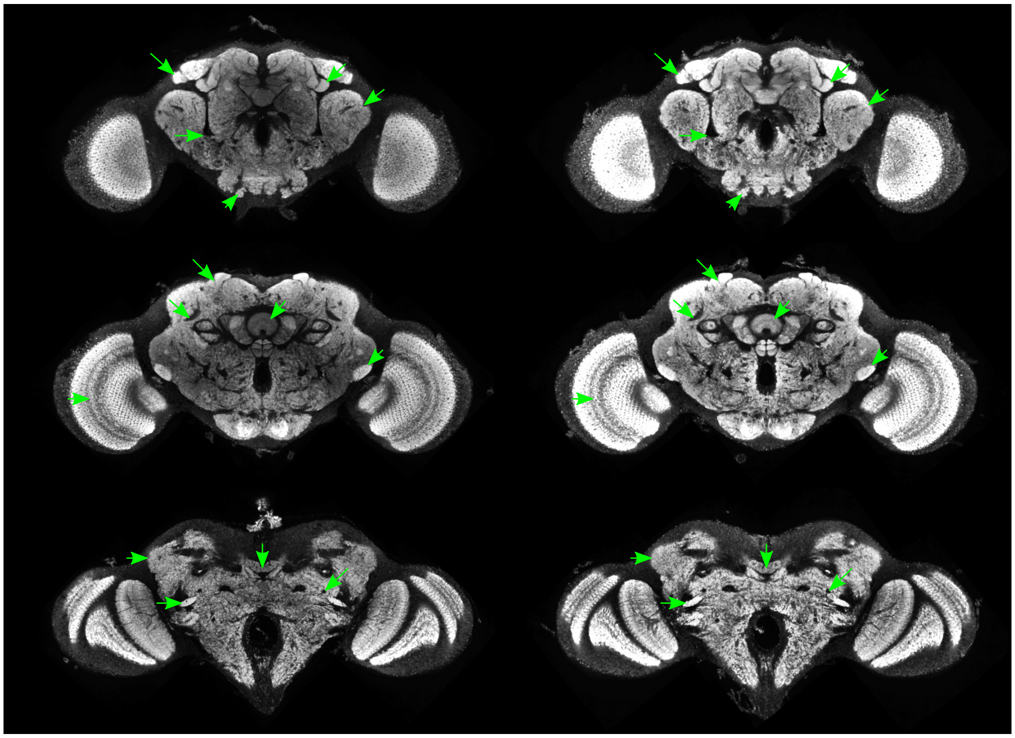

Fig S5. JRC2018 cmtkB

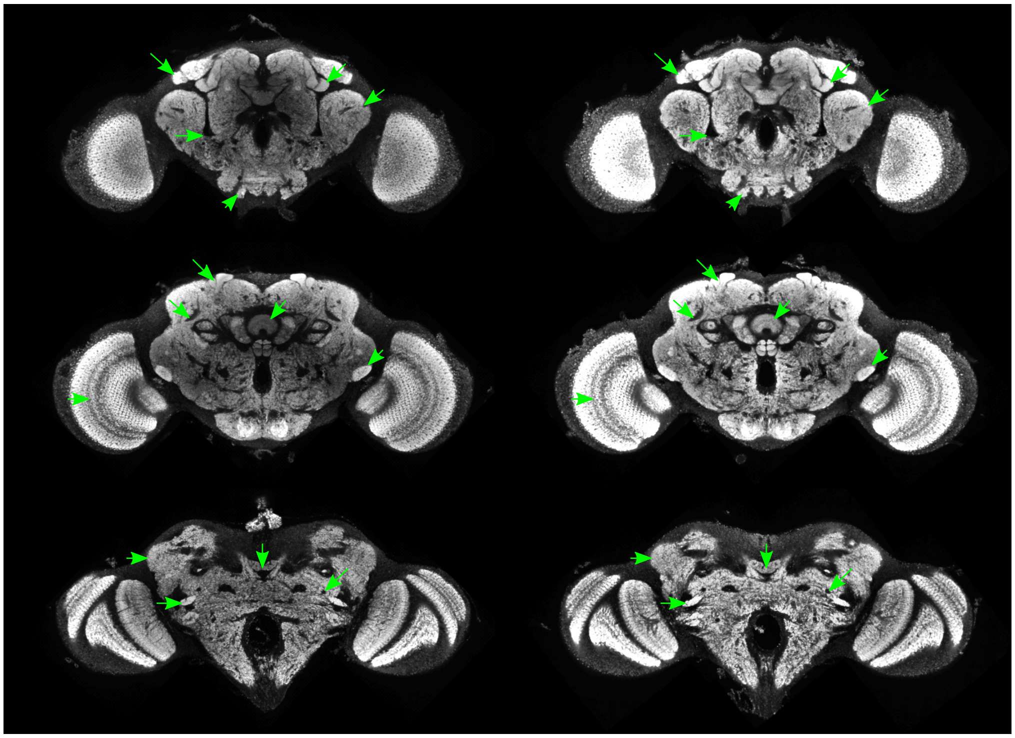

Fig S6. JRC2018 cmtkC

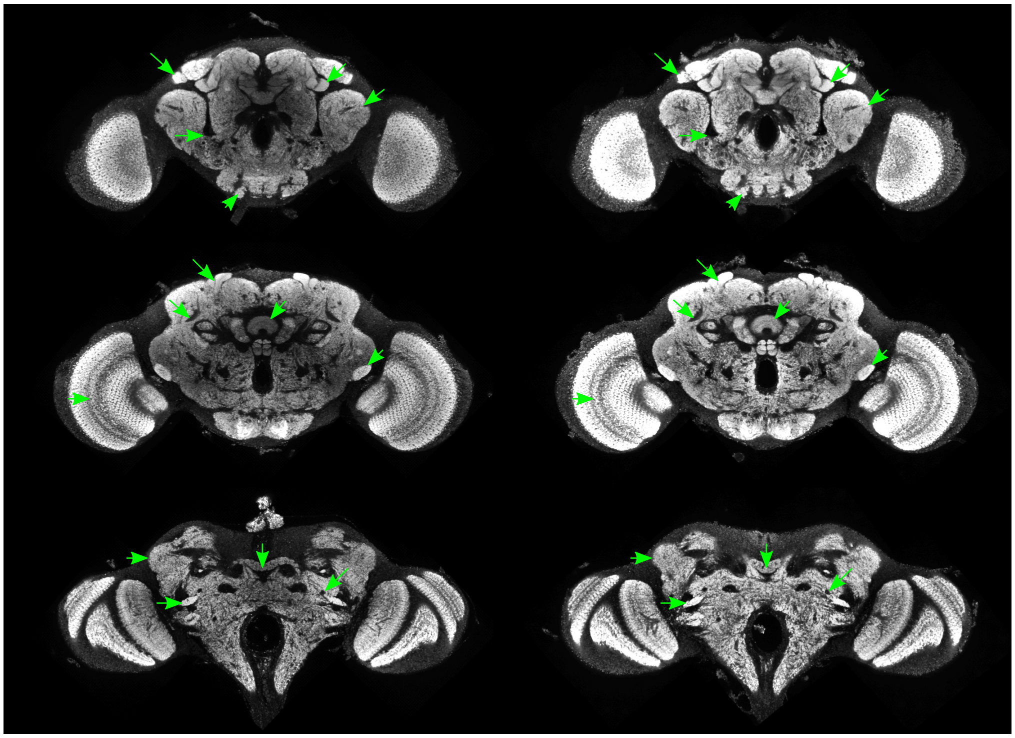

Fig S7. JRC2018 elastixA

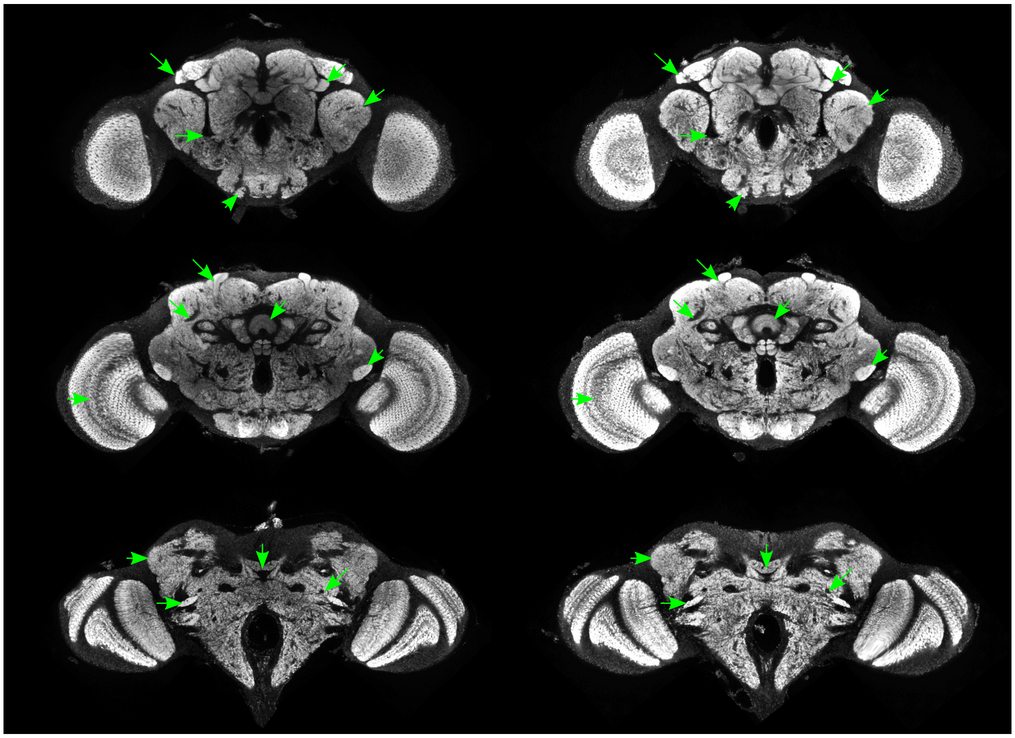

Fig S8. JRC2018 elastixB

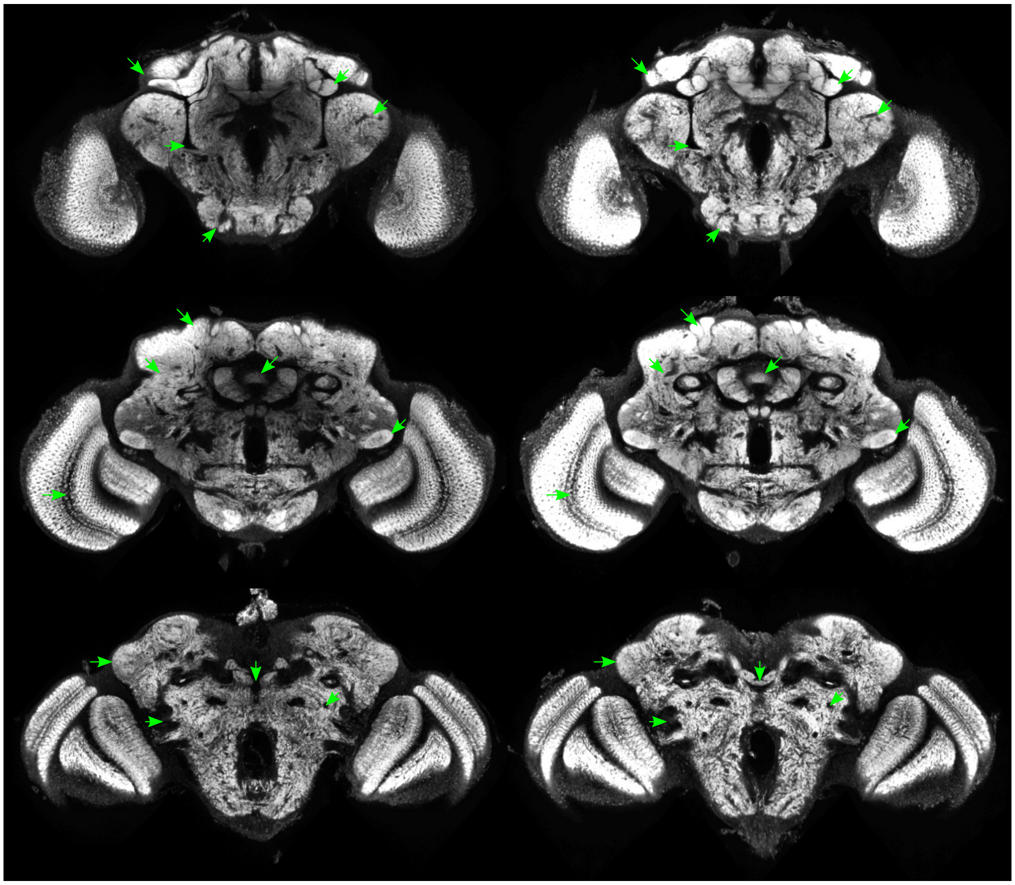

Fig S9. FCWB antsA

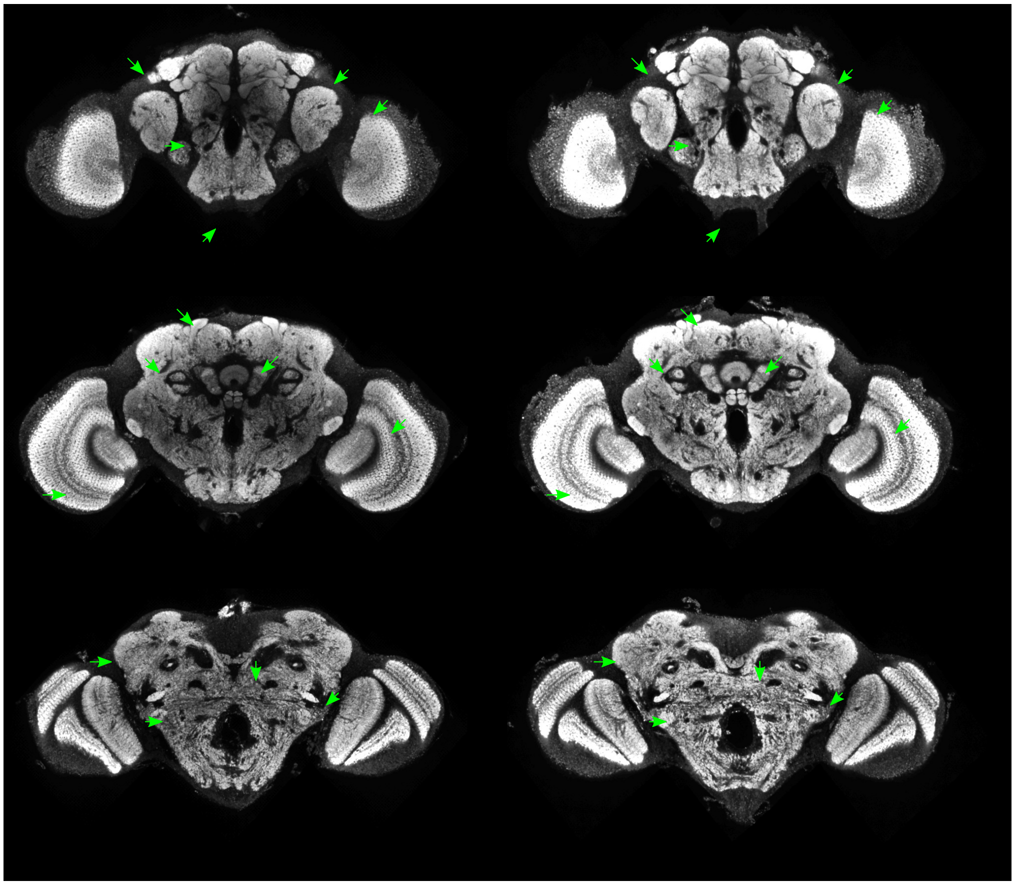

Fig S10. FCWB antsB

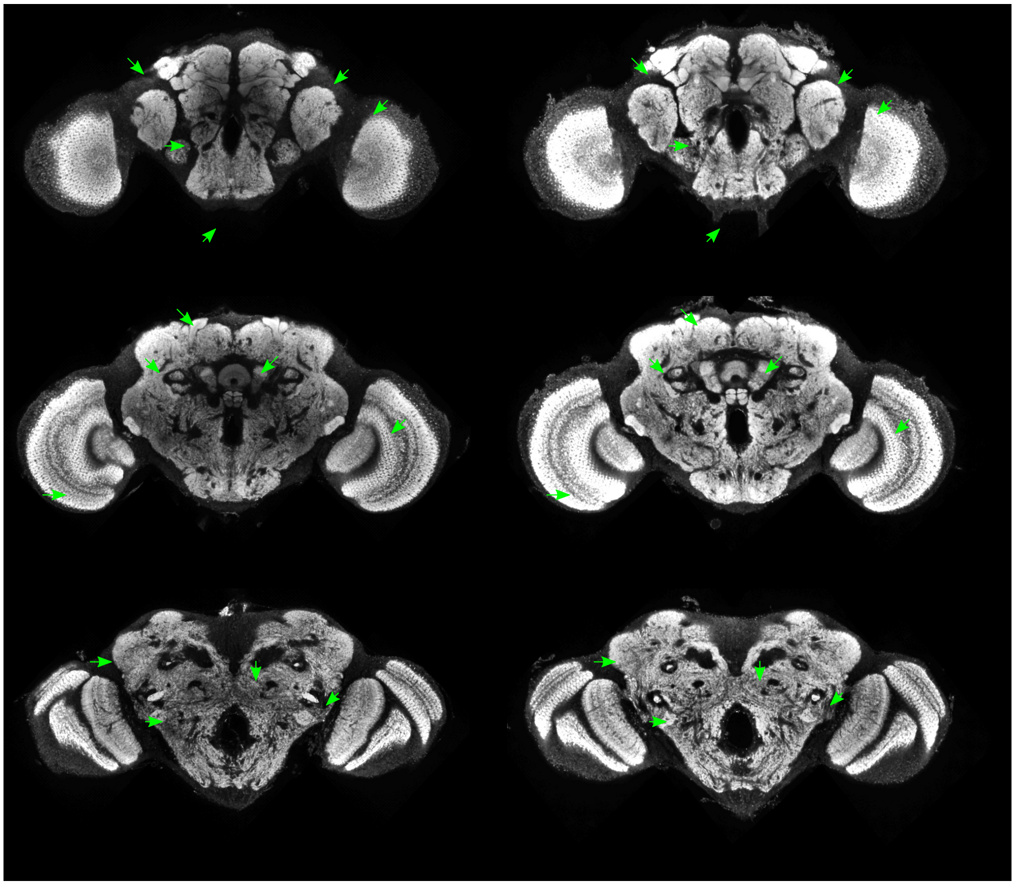

Fig S11. FCWB antsC

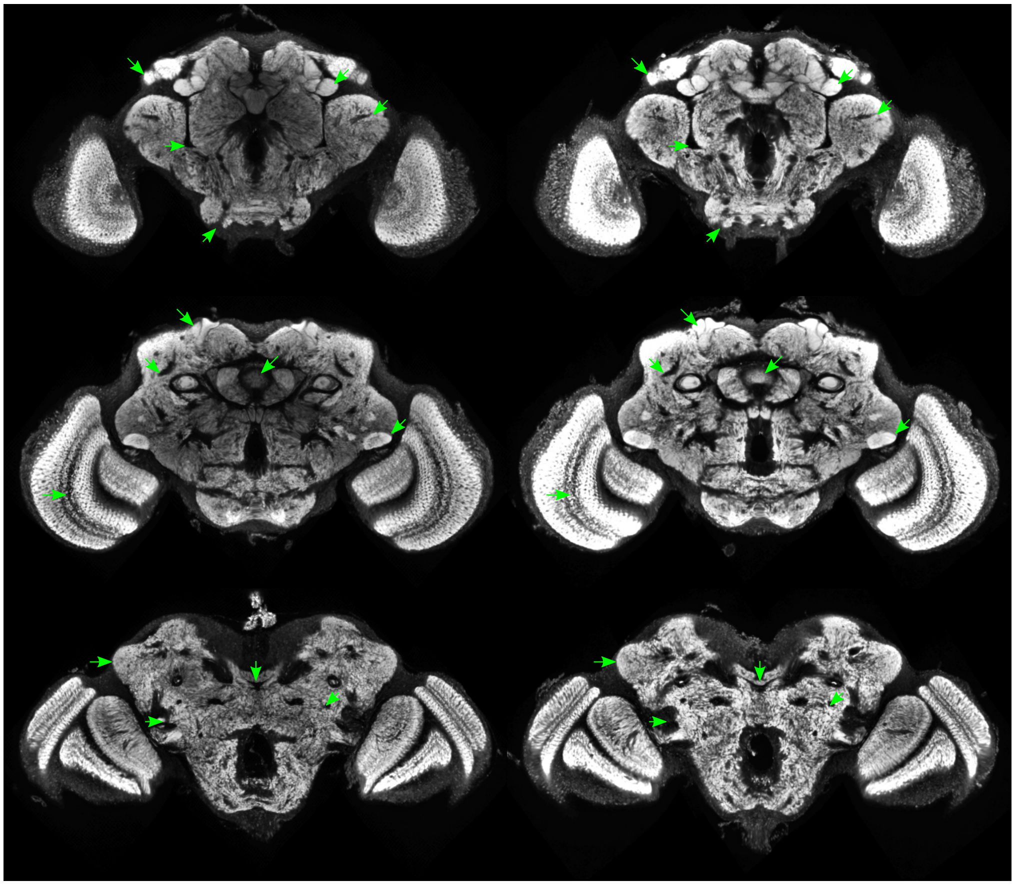

Fig S12. FCWB cmtkA

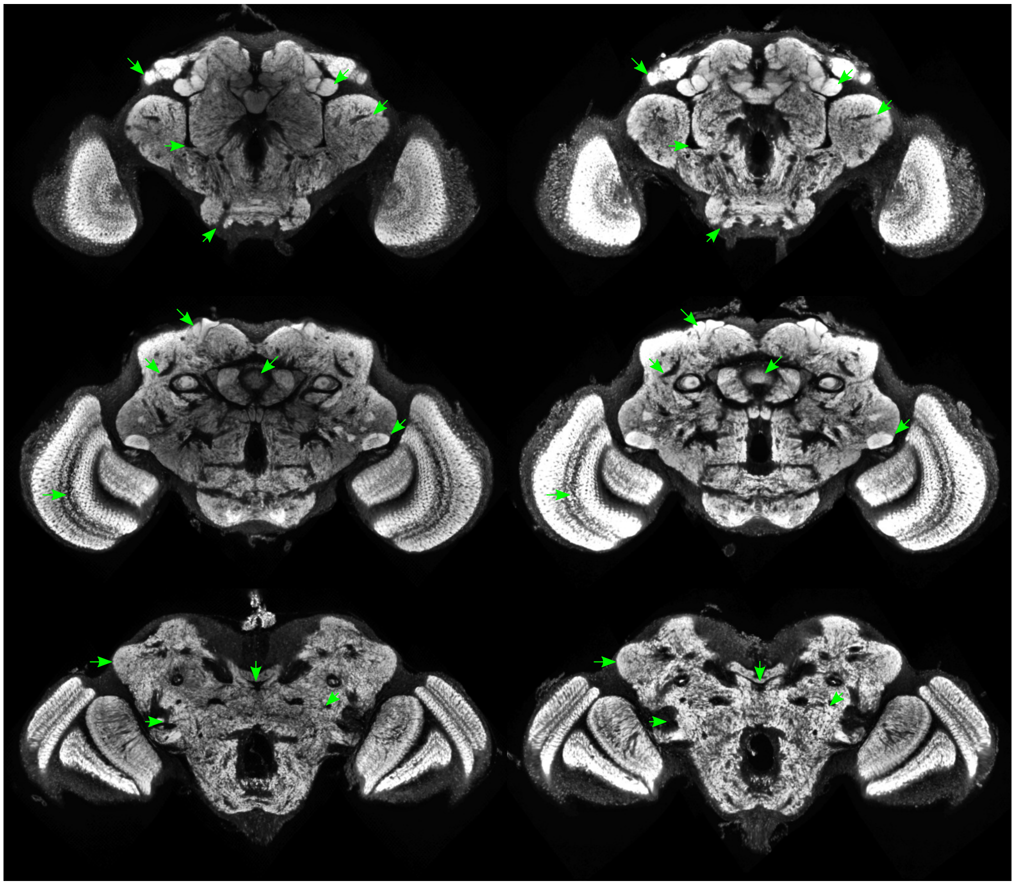

Fig S13. FCWB cmtkB

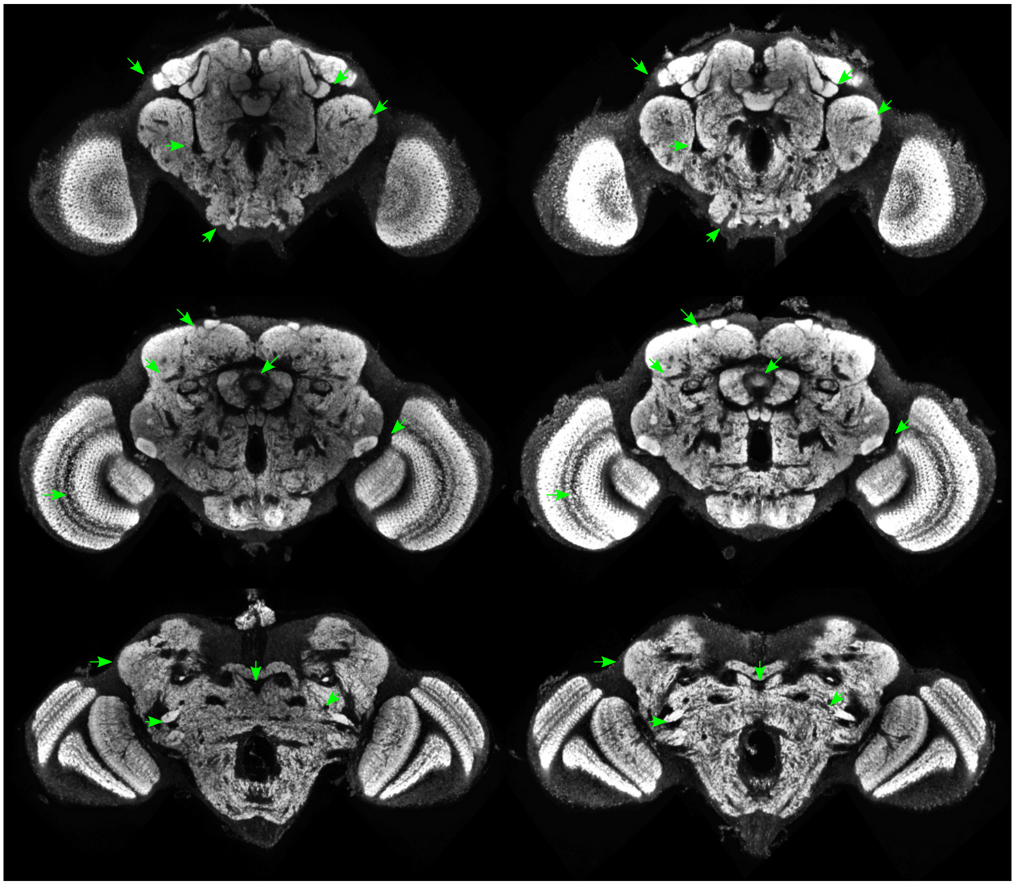

Fig S14. FCWB cmtkC

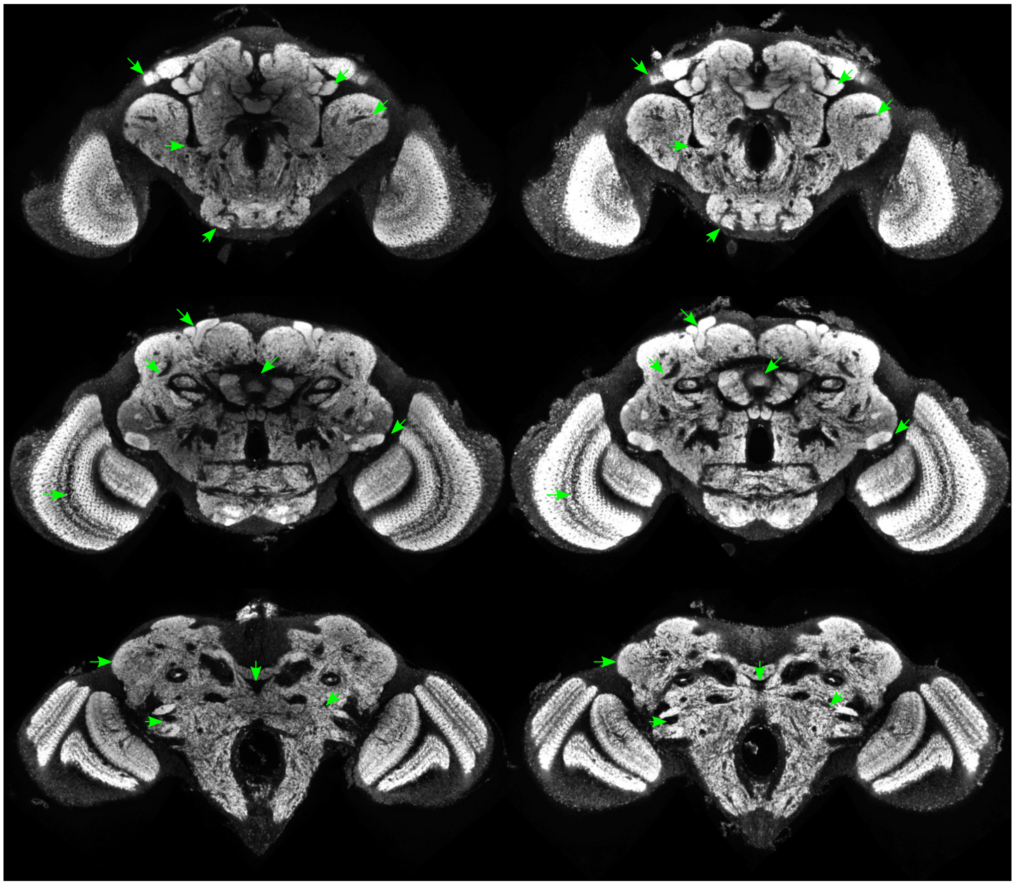

Fig S15. FCWB elastixA

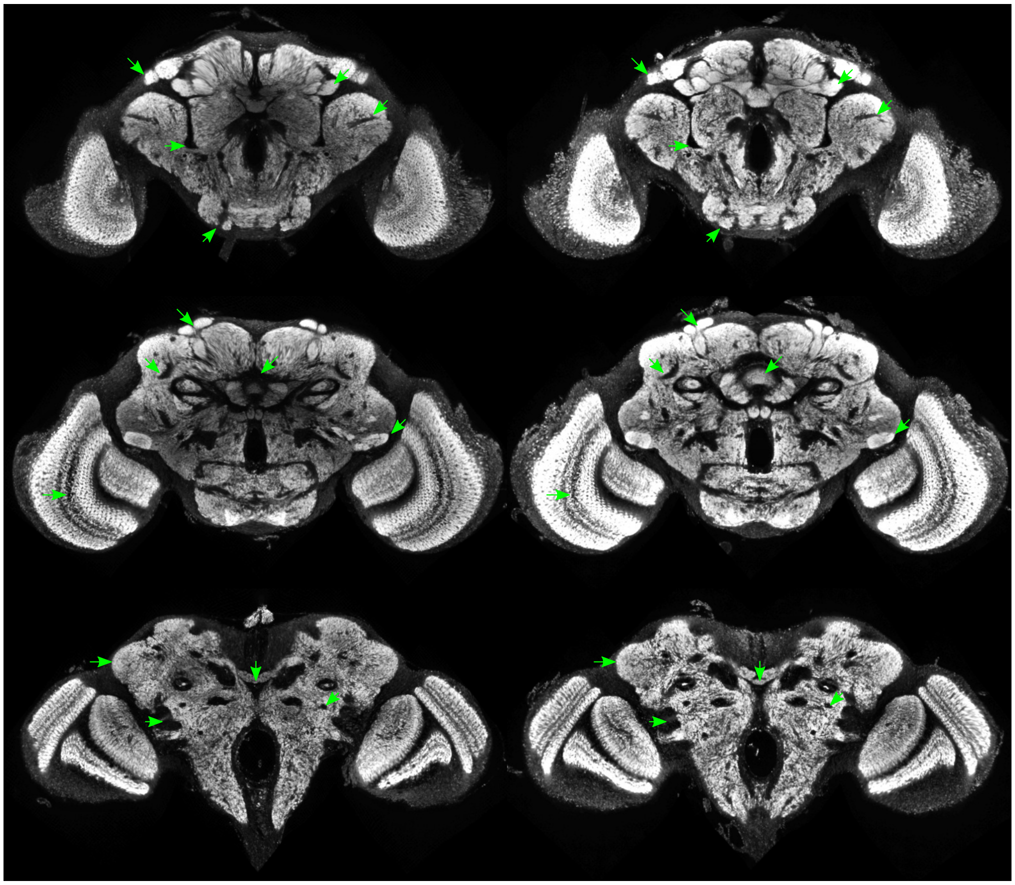

Fig S16. FCWB elastixB

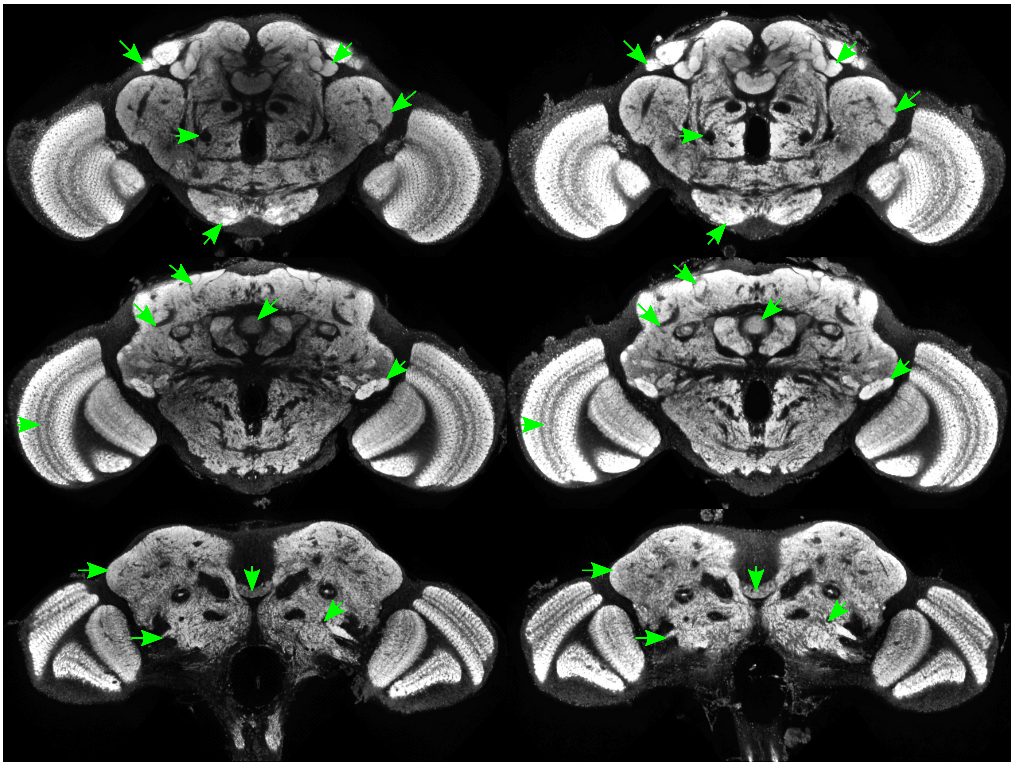

Fig S17. JFRC2013 antsA

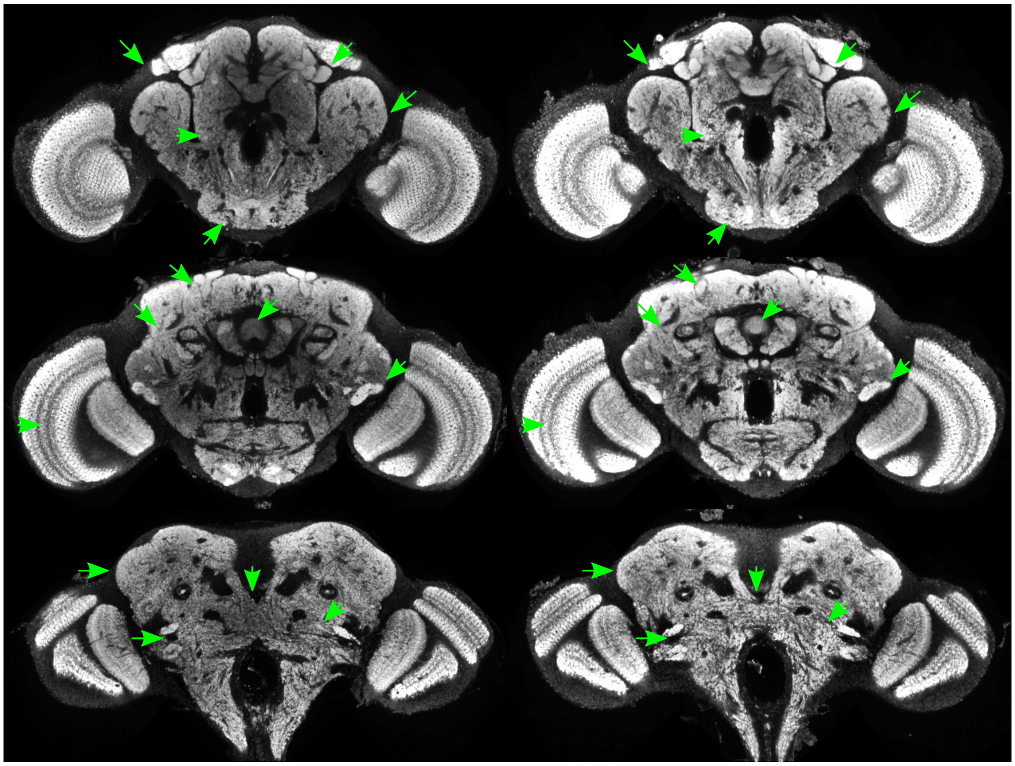

Fig S18. JFRC2013 antsB

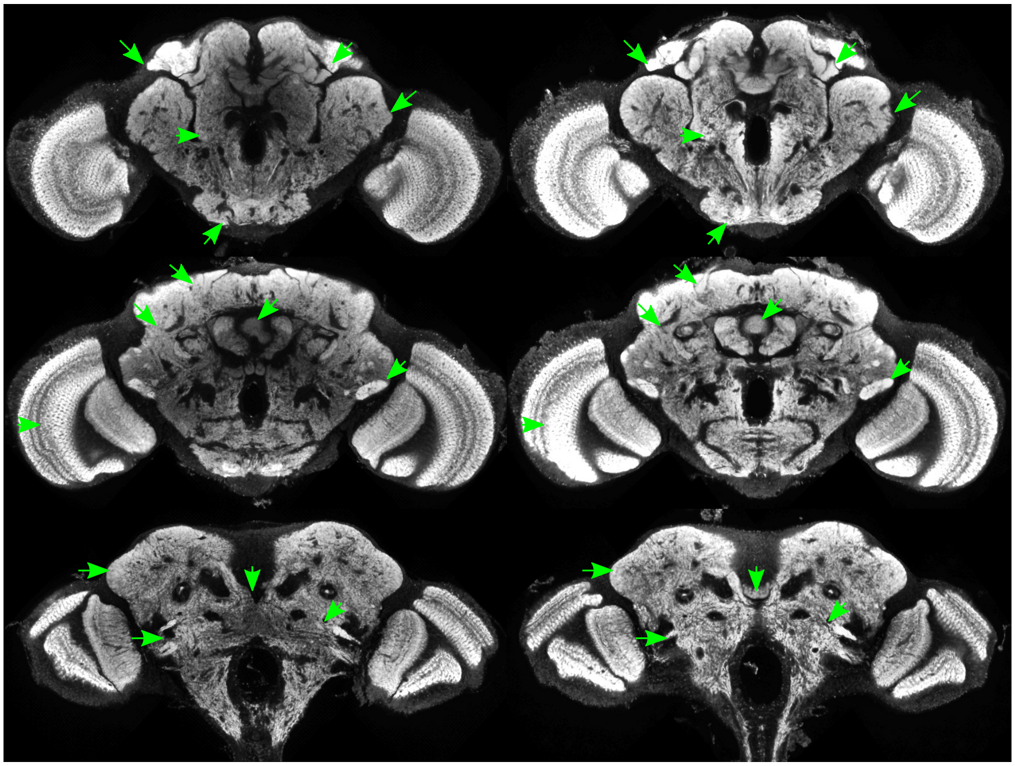

Fig S19. JFRC2013 antsC

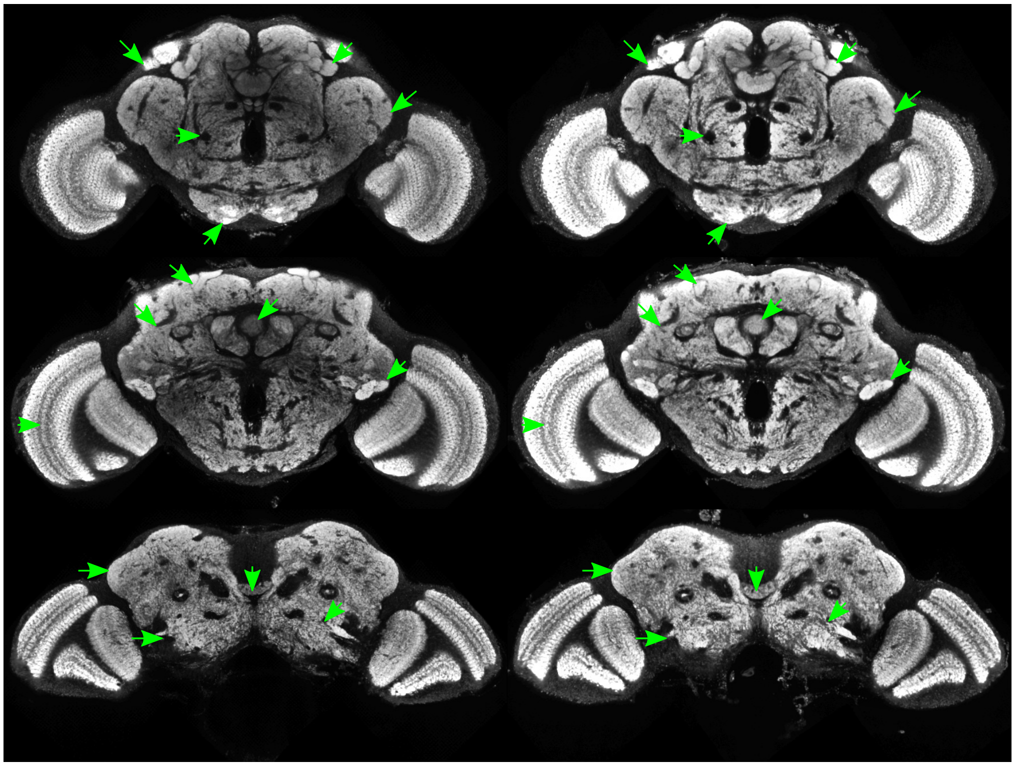

Fig S20. JFRC2013 cmtkA

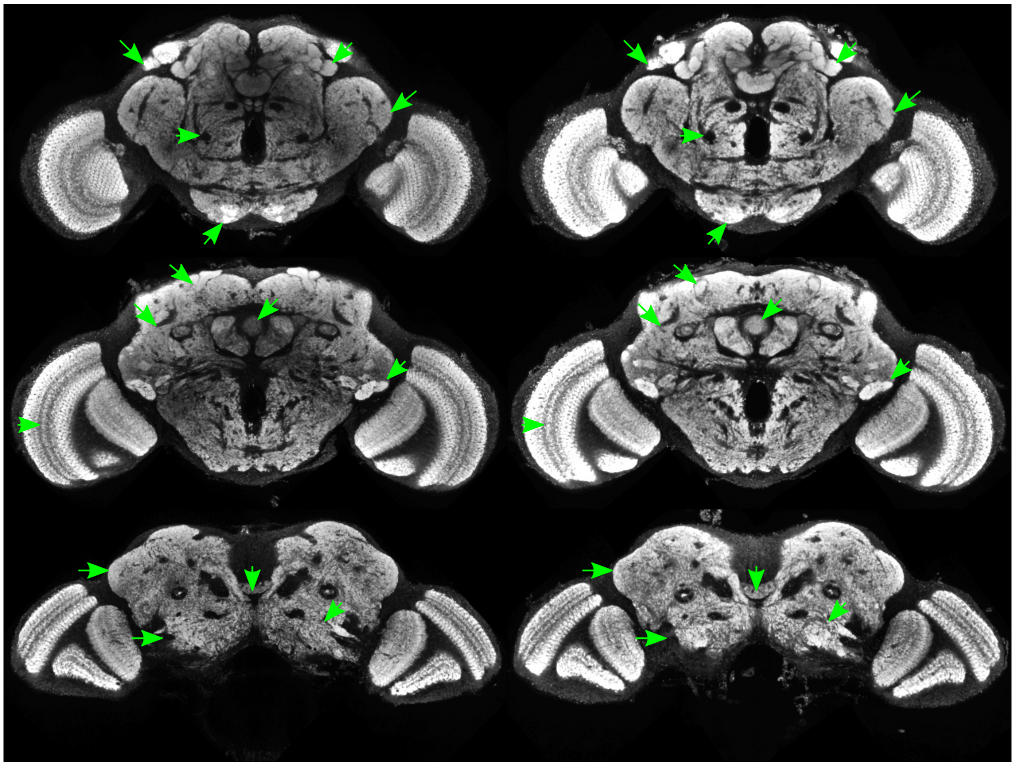

Fig S21. JFRC2013 cmtkB

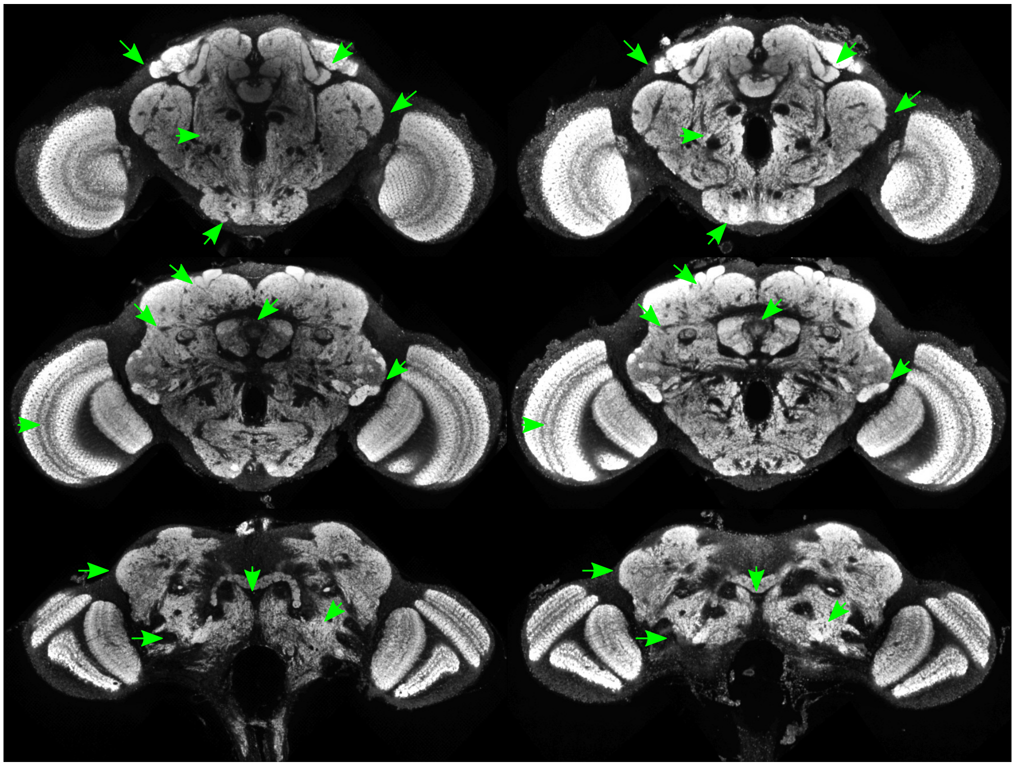

Fig S22. JFRC2013 cmtkC

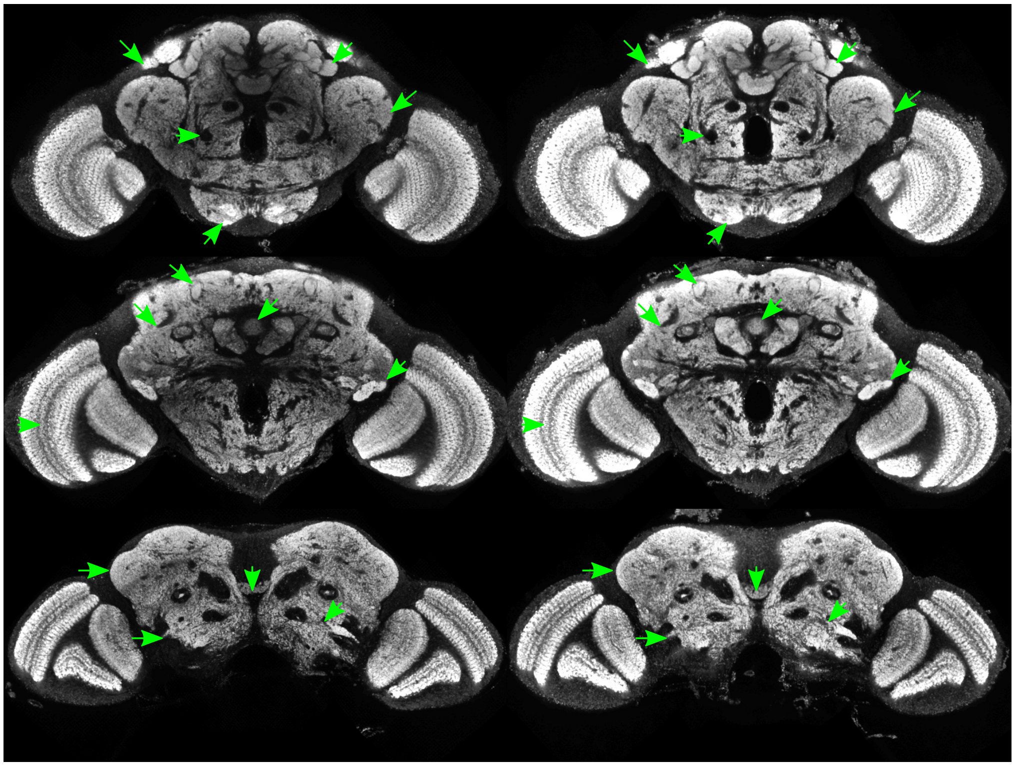

Fig S23. JFRC2013 elastixA

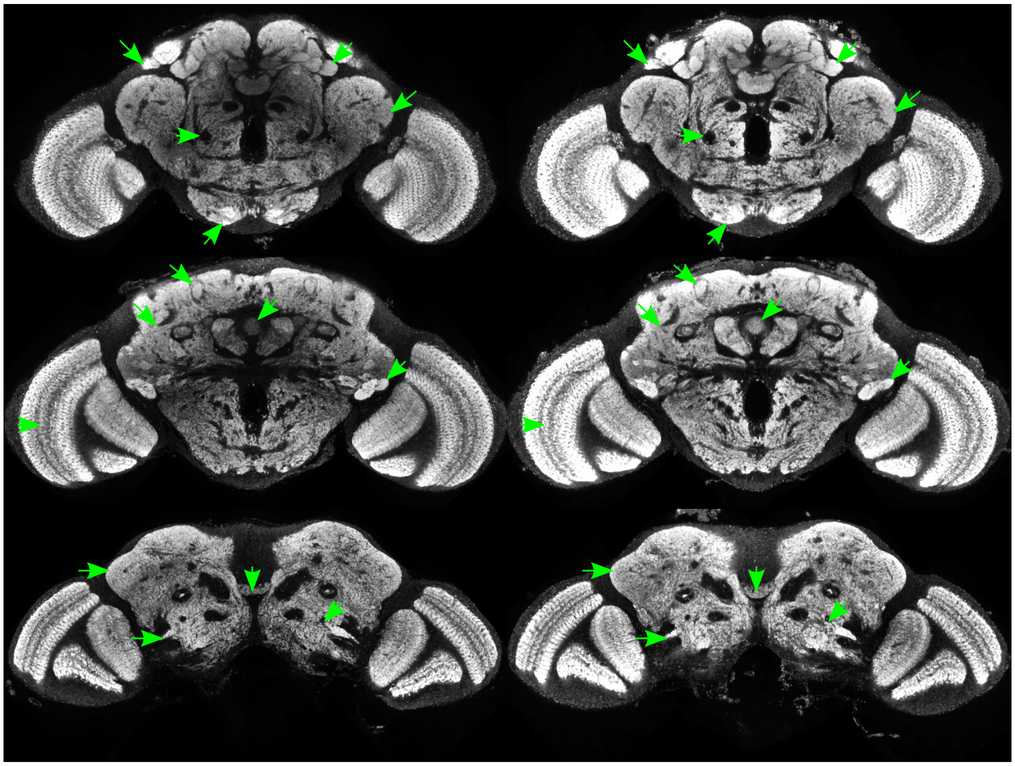

Fig S24. JFRC2013 elastixB

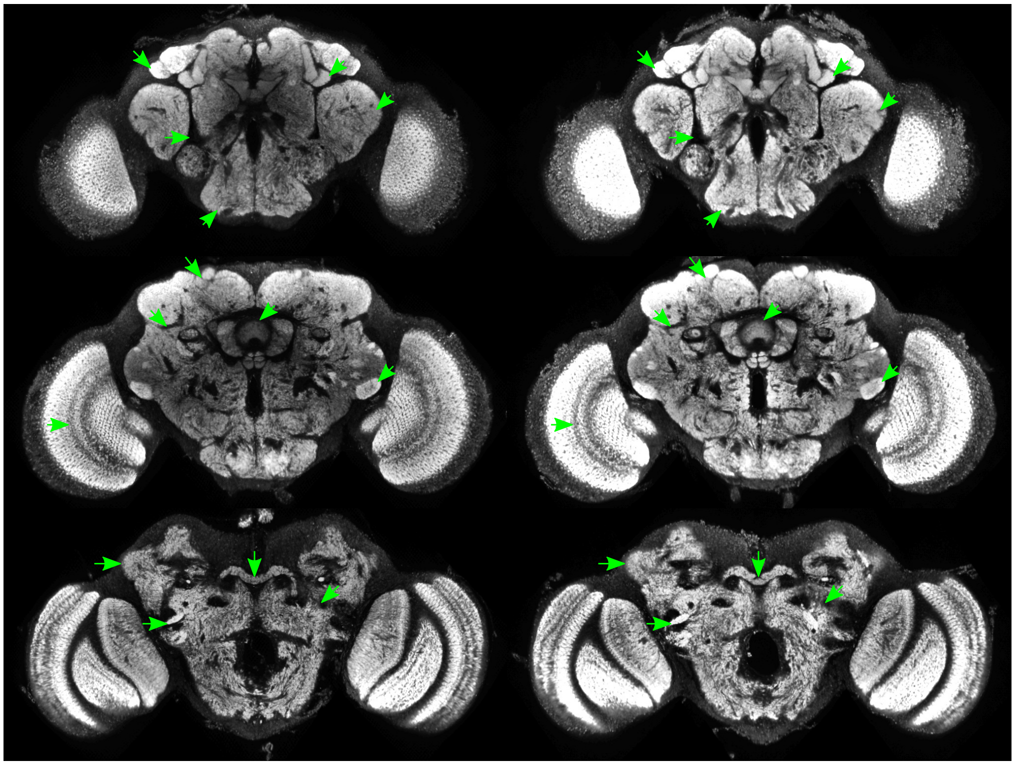

Fig S25. JFRC2010 antsA

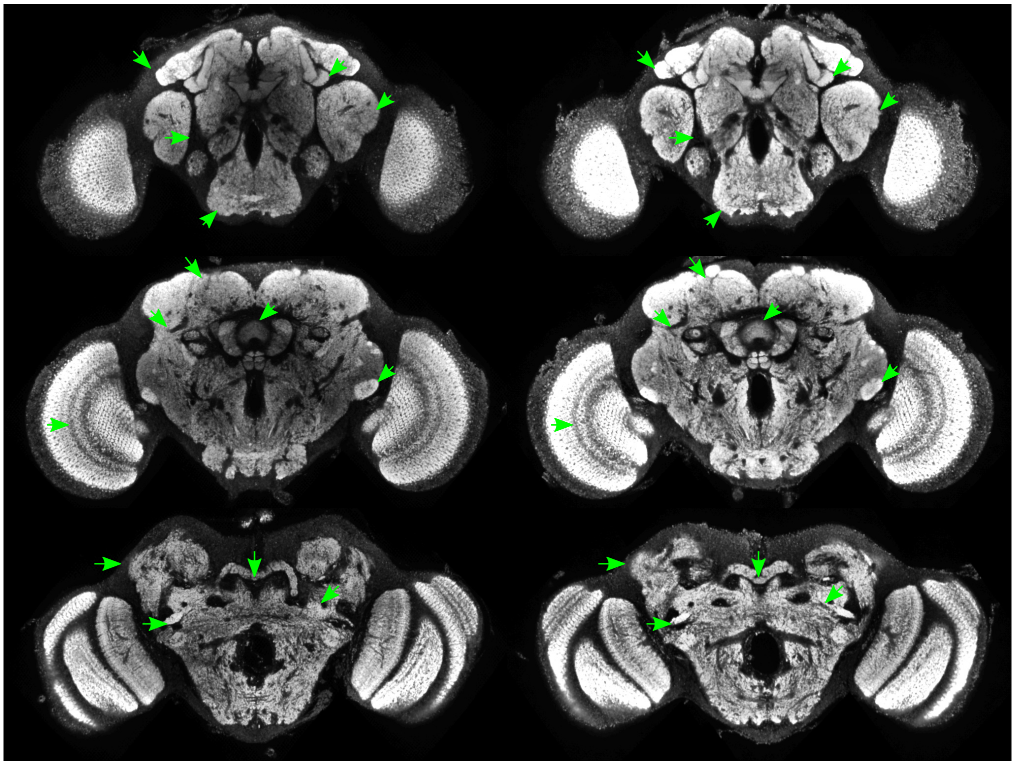

Fig S26. JFRC2010 antsB

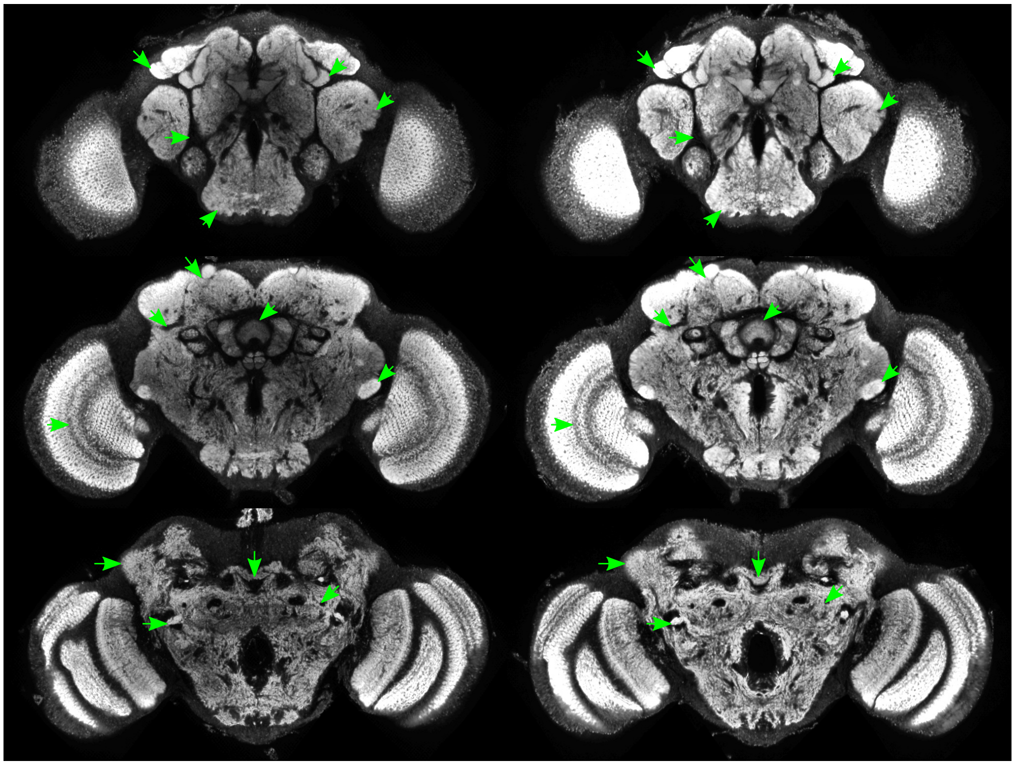

Fig S27. JFRC2010 antsC

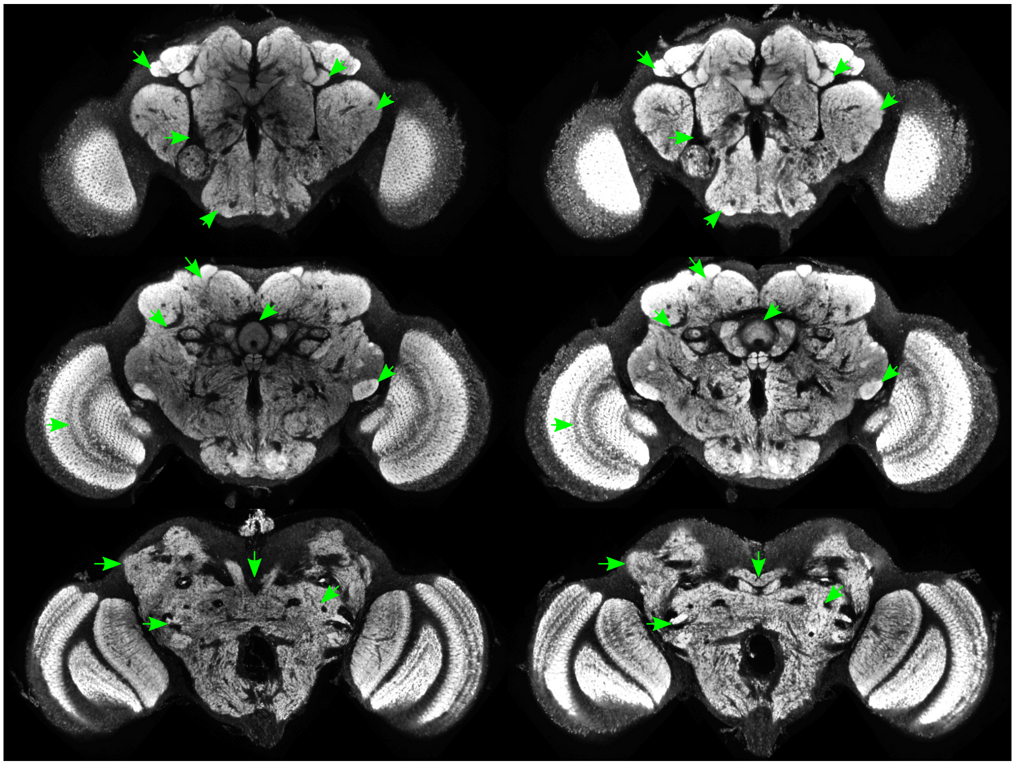

Fig S28. JFRC2010 cmtkA

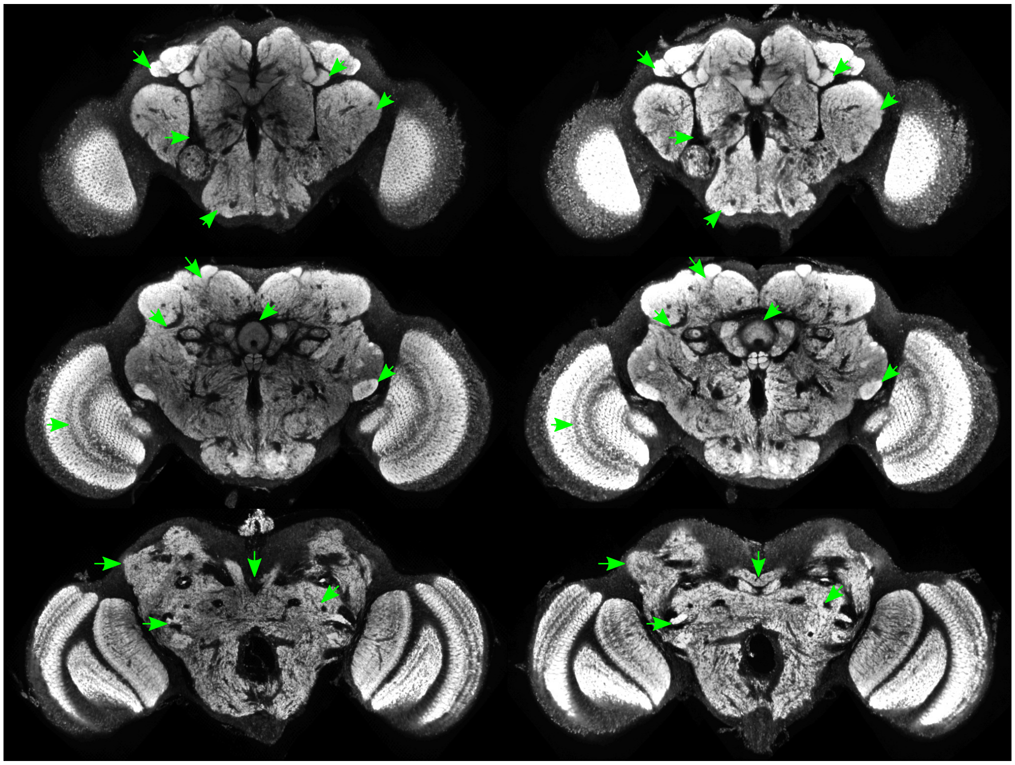

Fig S29. JFRC2010 cmtkB

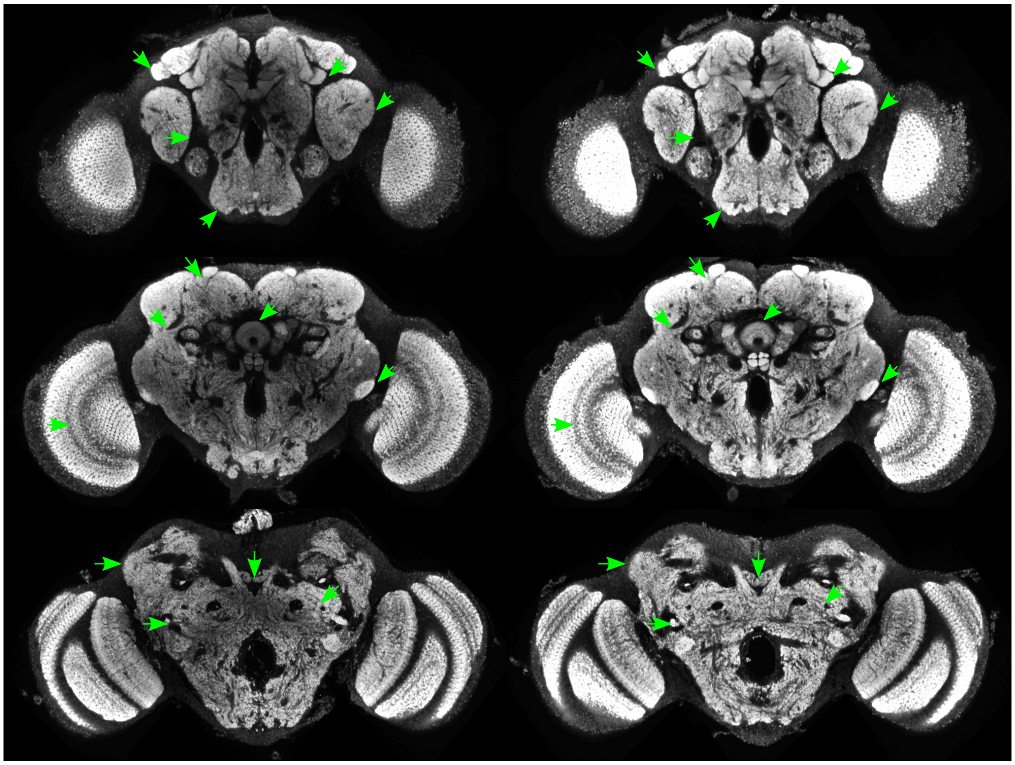

Fig S30. JFRC2010 cmtkC

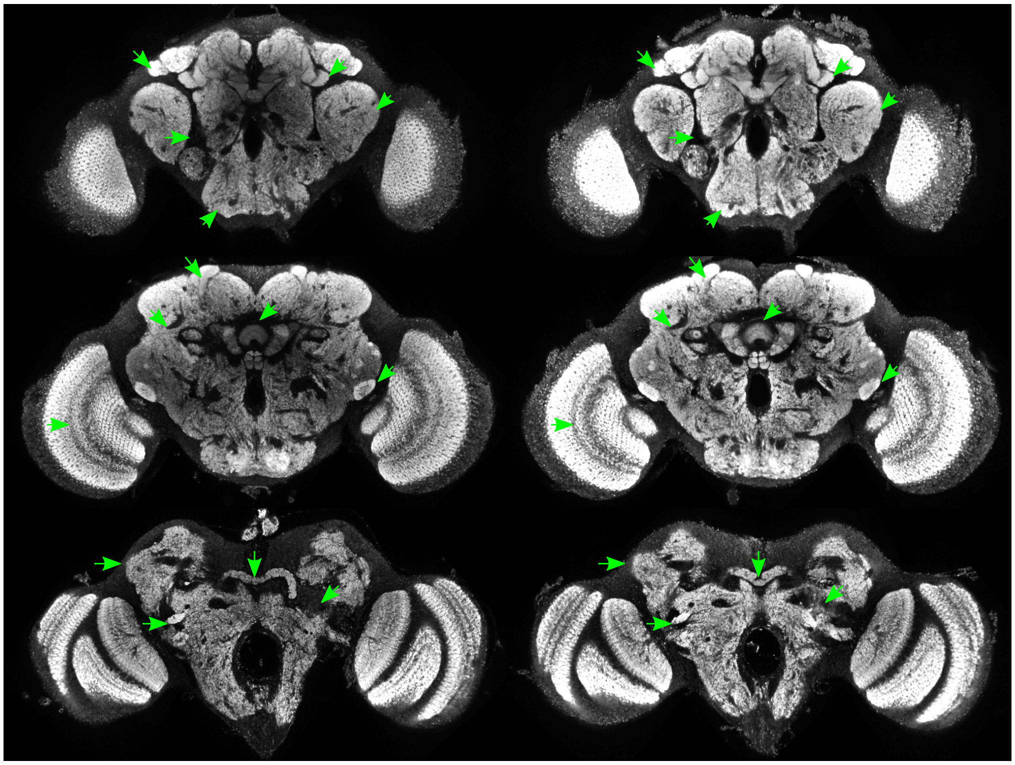

Fig S31. JFRC2010 elastixA

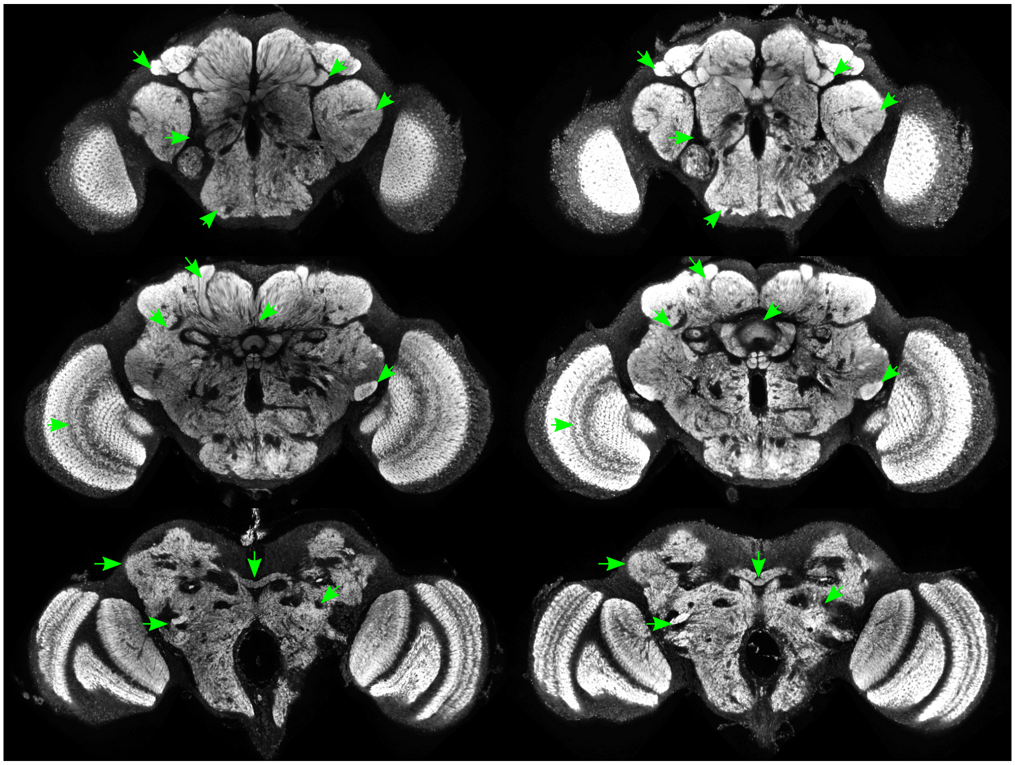

Fig S32. JFRC2010 elastixB

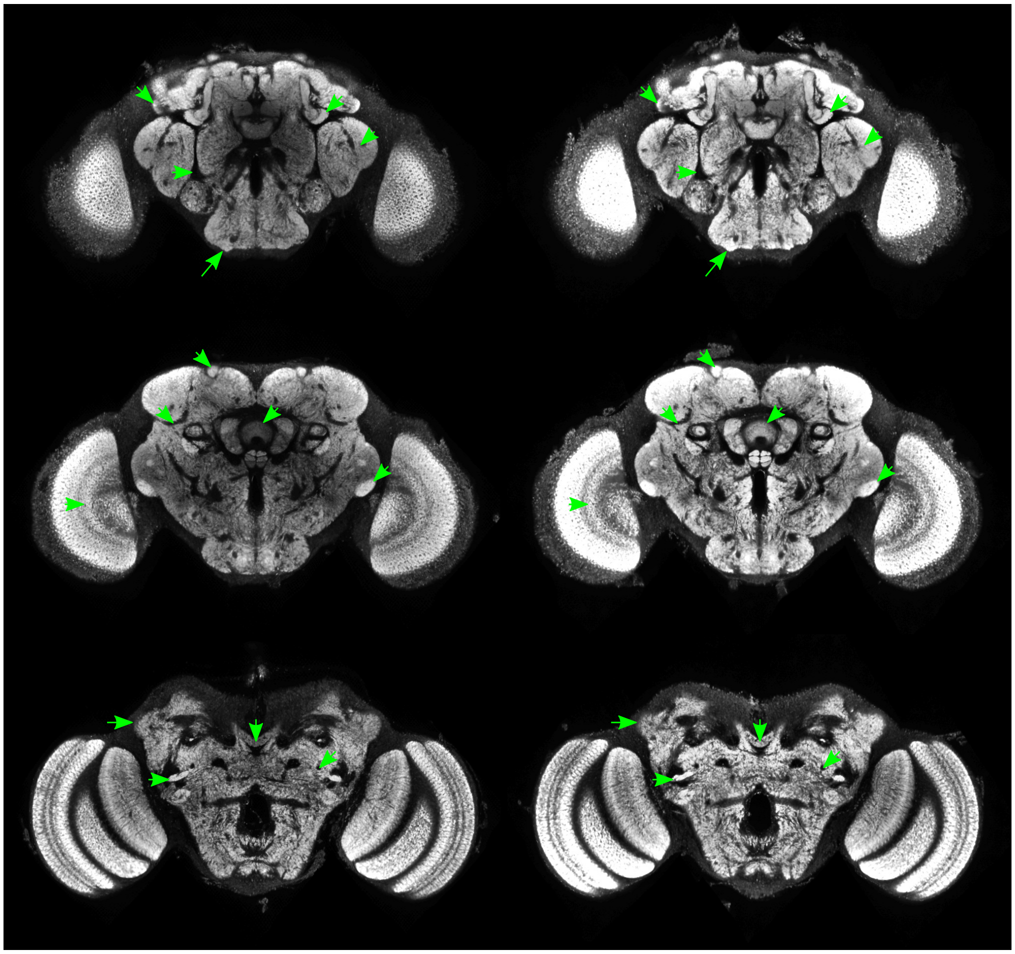

Fig S33. Tefor antsA

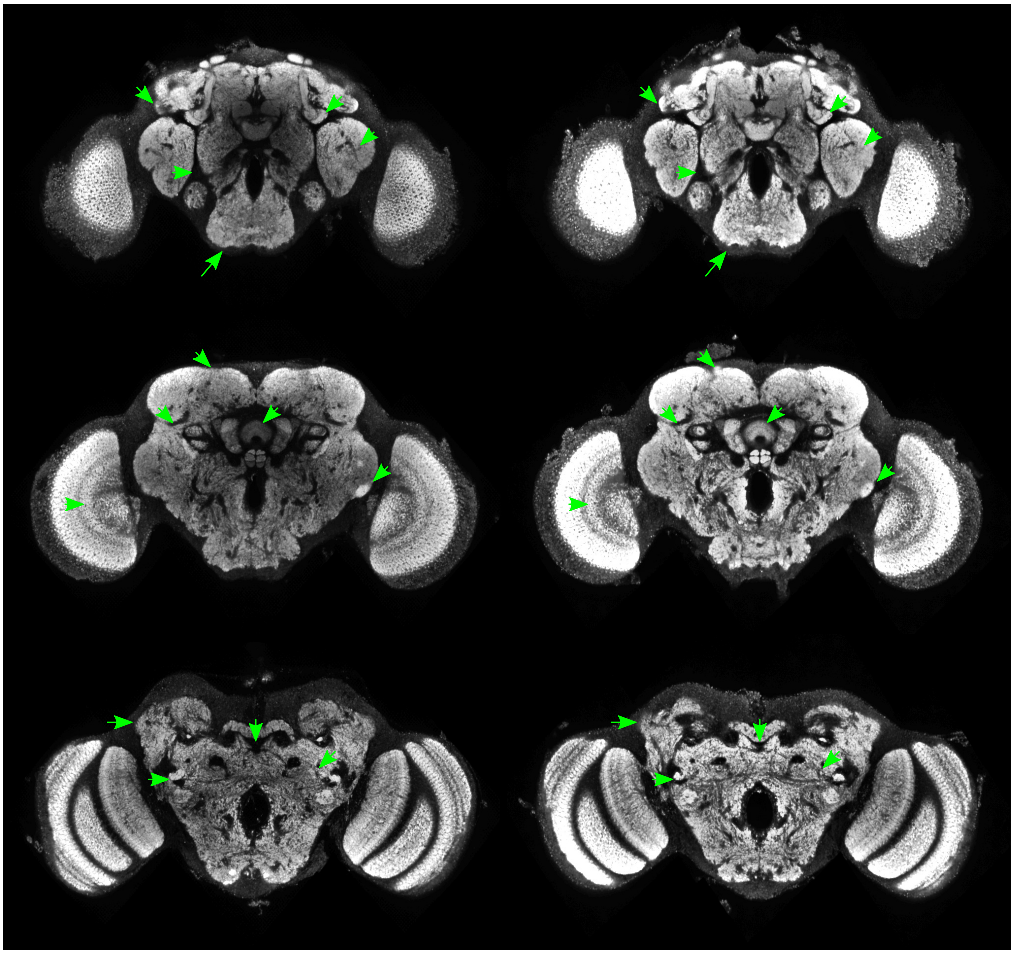

Fig S34. Tefor antsB

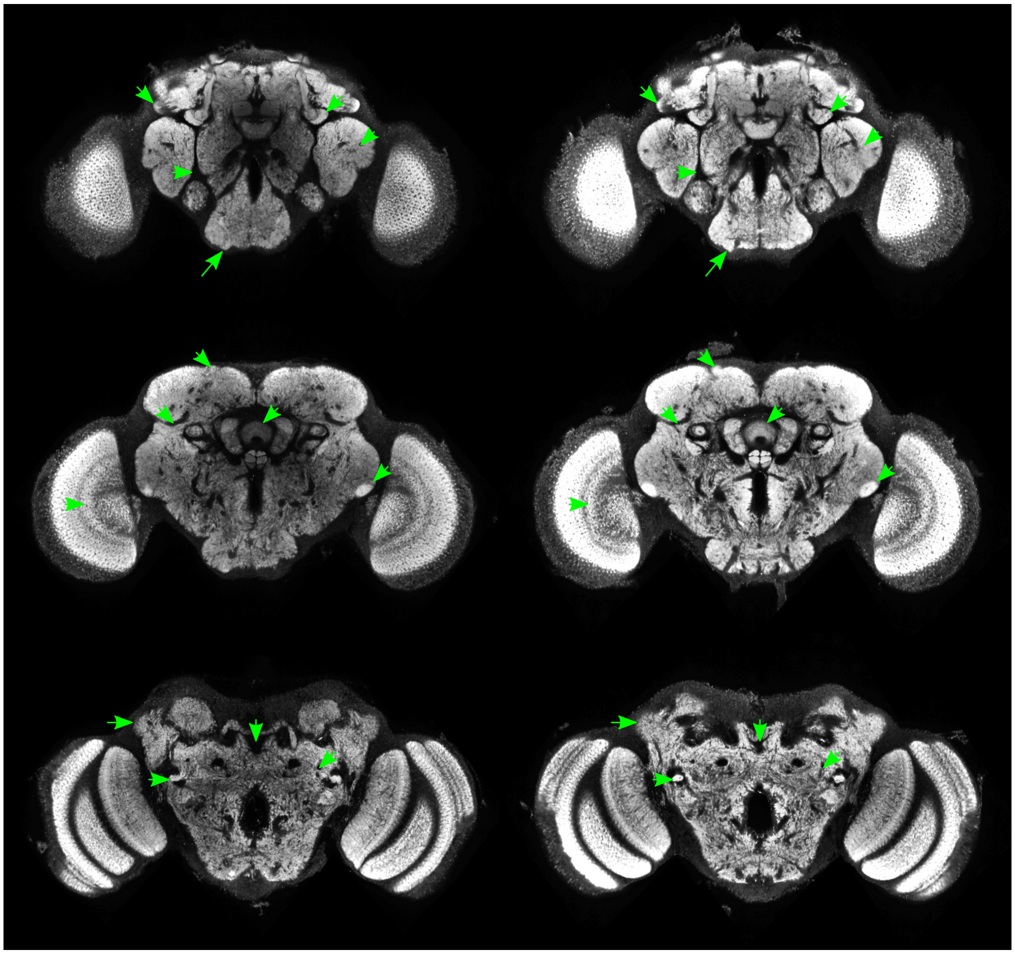

Fig S35. Tefor antsC

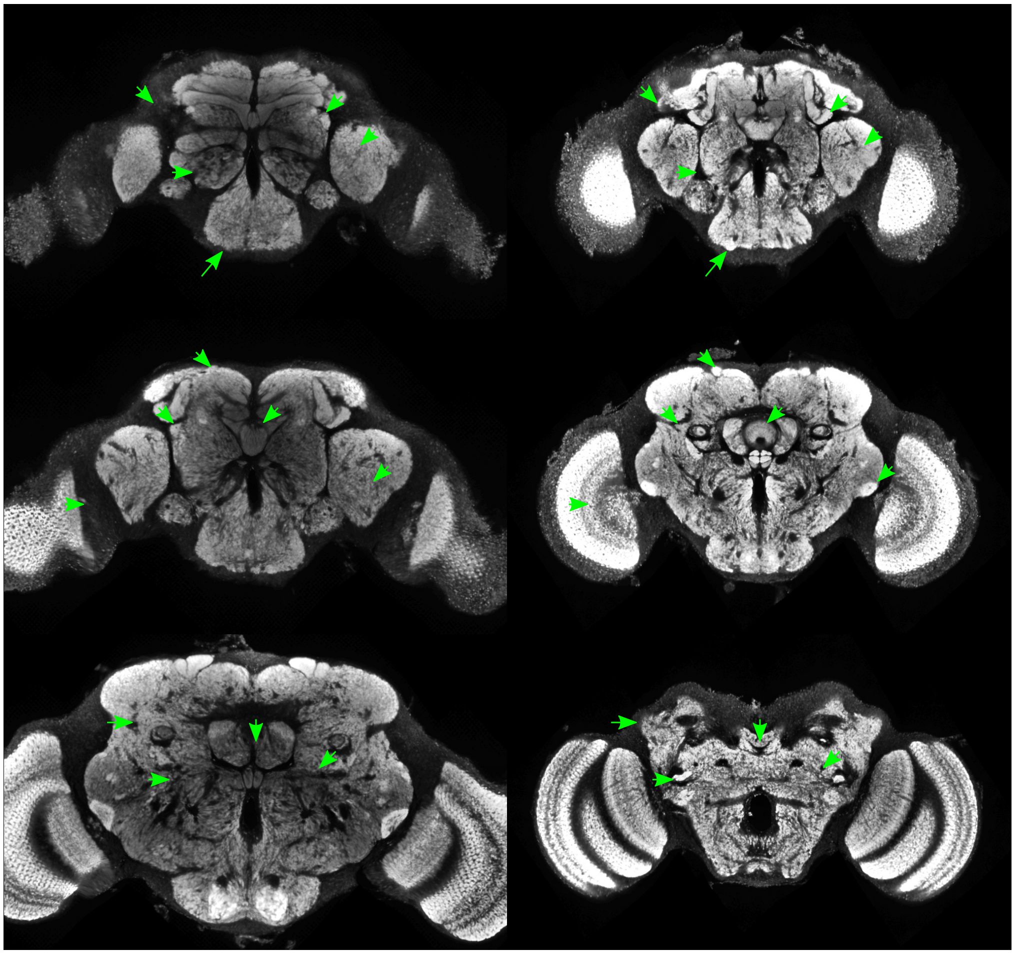

Fig S36. Tefor cmtkA

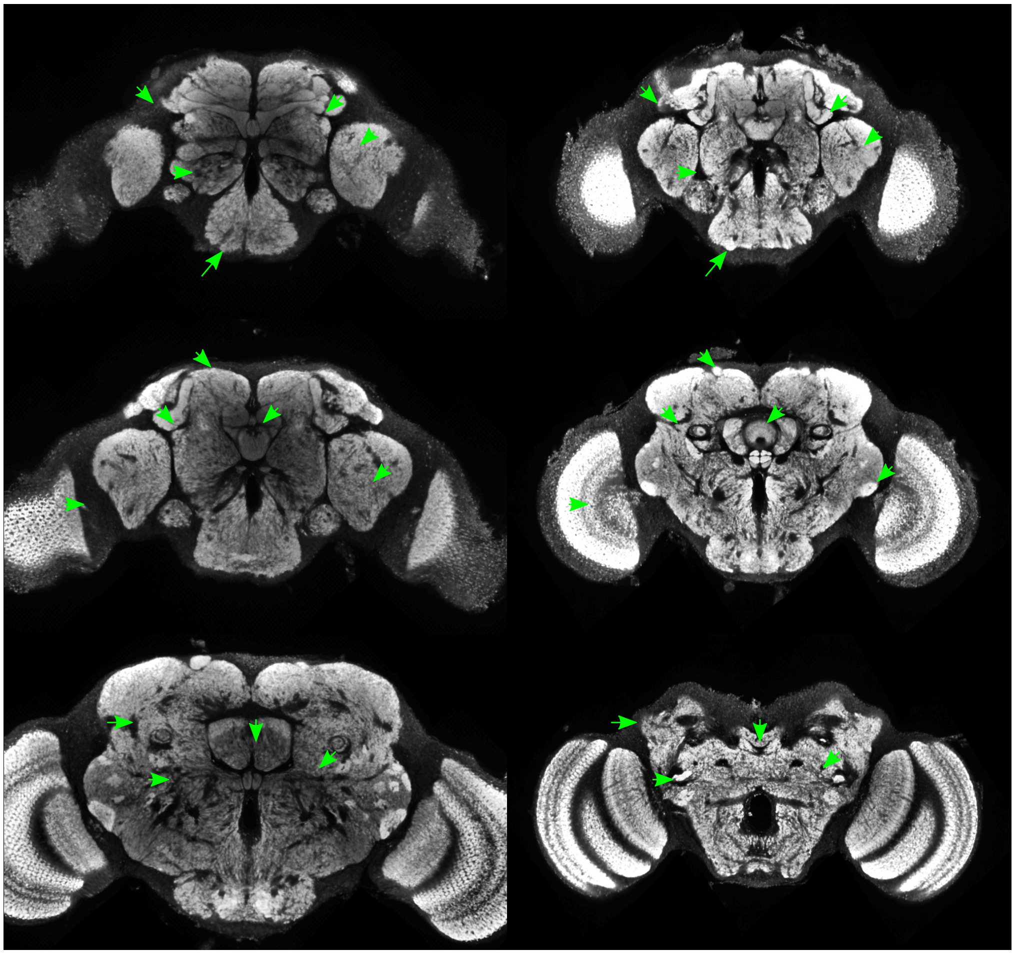

Fig S37. Tefor cmtkB

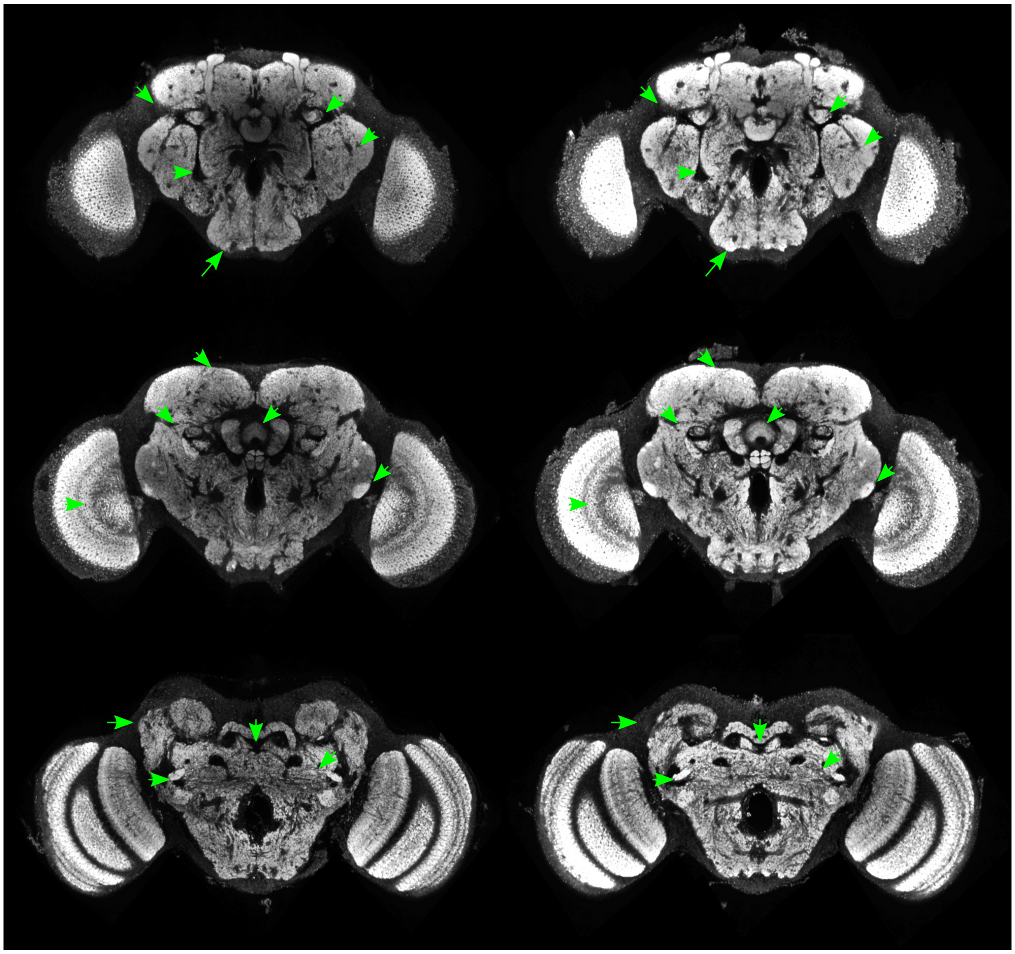

Fig S38. Tefor cmtkC

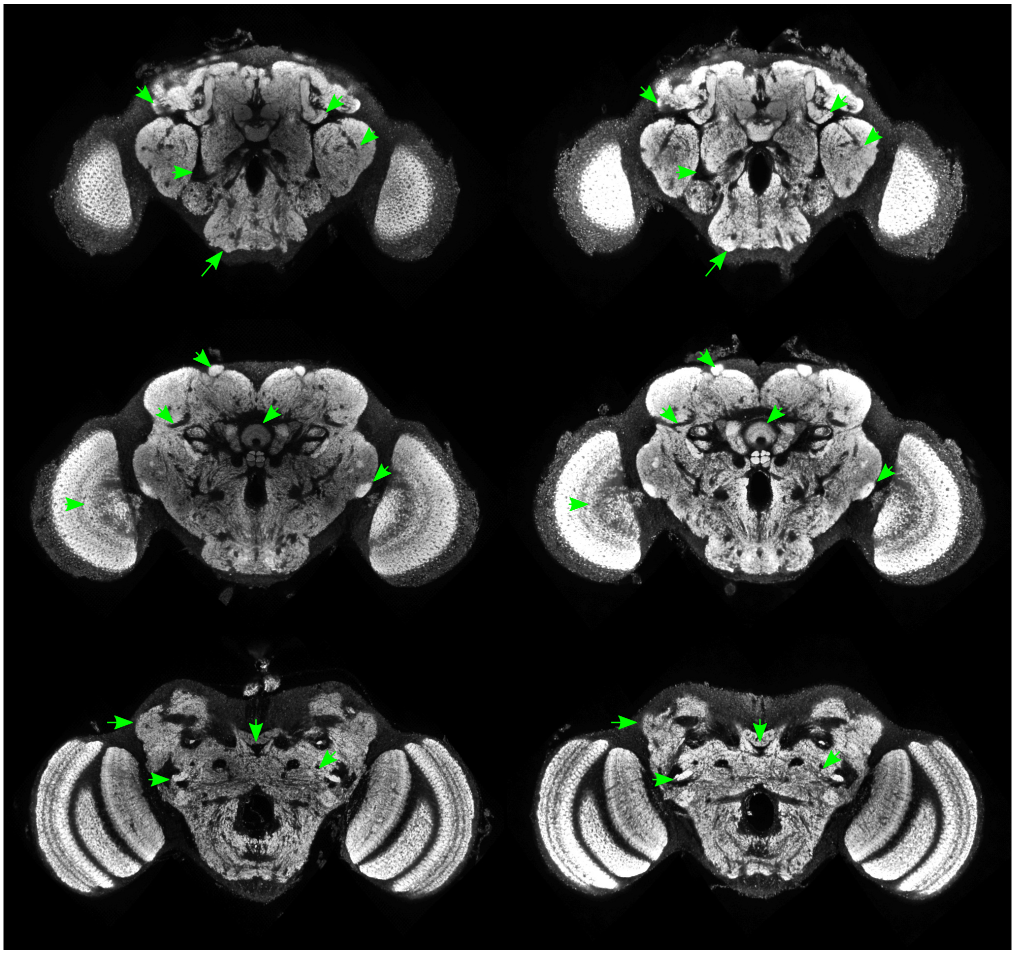

Fig S39. Tefor elastixA

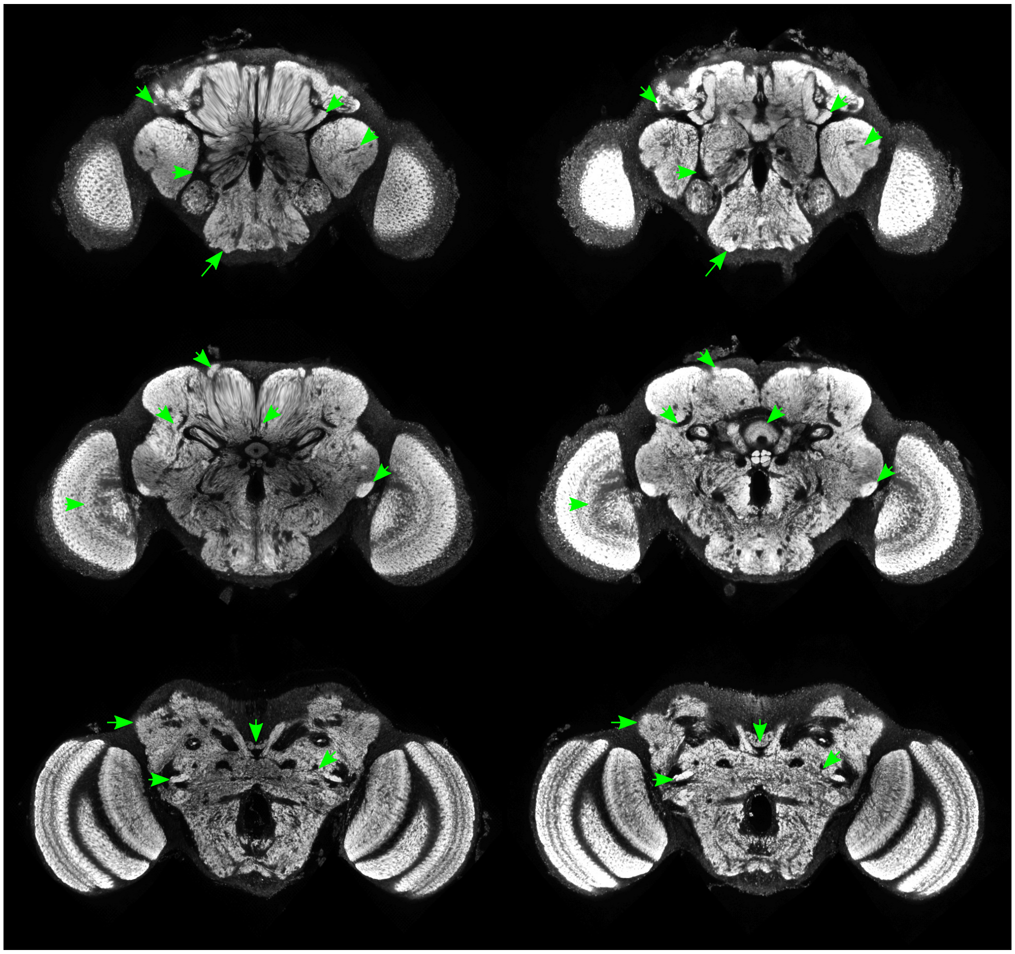

Fig S40. Tefor elastixB
