# Supplementary figures and images for: An unbiased template of the Drosophila brain and ventral nerve cord
Source: PLoS One. 2020 Dec 31;15(12):e0236495. doi: 10.1371/journal.pone.0236495 (PMC7774840; doi:10.1371/journal.pone.0236495)

FAFB  
ssTEM

(a)

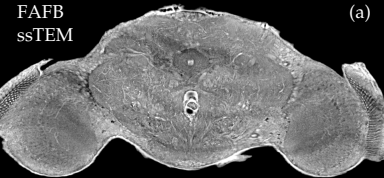

FAFB  
synapses

(b)

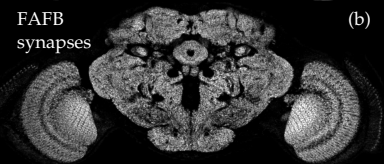

JRC 2018  
atlas

(c)

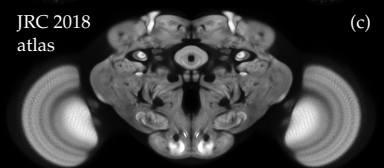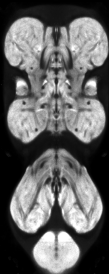

100  $\mu$ m

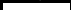

Supplement: S3 File — (PDF) [file pone.0236495.s003.pdf]
