# Supplementary material for: An unbiased template of the Drosophila brain and ventral nerve cord
Source: PLoS One. 2020 Dec 31;15(12):e0236495. doi: 10.1371/journal.pone.0236495 (PMC7774840; doi:10.1371/journal.pone.0236495)
Supplement: Attachment — Submitted filename: Response_To_Reviewers.pdf [file pone.0236495.s004.pdf]

We thank the anonymous reviewer for their comments. We have addressed these in our revision and feel that the clarity and accessibility of the manuscript are improved as a result. Since our initial submission, we have made public a new transformation from our template to a newly released *Drosophila* electron microscopy dataset, called the "hemibrain". Our changes are highlighed in blue in the "Revised Manuscript with Track Changes," with footnotes indicating the comment each change addresses.

A summary of the changes are given below.

1 Reviewer #1

1.1

It would be great to have a brief and simple description of how to use the atlas right up front in the manuscript. It is fantastic that the templates, transformations, code, and descriptions are all publicly available, but a brief paragraph within the manuscript that describes the use of these resources would likely attract many researcher to actually try it out.

Reply: We thank the reviewer for the suggestion and considering the accessibility of this work. We have added a brief new section at the end of the introduction ("Usage") in which we describe the ways in which the resources we have created will be useful to various researchers in their own work.

1.2

It is understood that registration of genetically labeled neurons requires to have a standard of non-wildtype Drosophila. However, it might be useful to include a short paragraph of the origin of the respective genetic backgrounds. This would greatly help researchers, that are not as experienced in Drosophila genetics as the authors are, to judge on potential differences /caveats when they will try to move forward and register data from their own work with different fly strains.

Reply: We have included a short discussion regarding the potential caveats when using this work with *Drosophila* of different genetic backgrounds, of which be believe there to be very few. As evidence for this, we point out our own internal success in registering many thousands of brains and VNCs from different genetic lines.

1.3

It seems a little harsh to state that the existing VNC atlas of Börner is not available at all. Although I have not made the effort to try to find it myself, I know people who have accessed it before. However, it is agreed that it is not as advanced as the one of this study, has not been analyzed nearly as deeply as the work presented, and most importantly, it is not useable with open access software which indeed significantly limits availability to the field. In addition to the high quality, being able to use open source code and software is a compelling advantage of the work presented.

Reply: We echo the reviewer's appreciation of open data and software. We have softened our language slightly, but feel it is important to recognize this hurdle.

1.4

A statement about the obvious tissues distortions, especially non-isometric shrinkage, as expected from the fixation, dehydration, and clearing protocols used for histology would be useful. Especially in the

light of the expected increasing availability of live imaging data (without fixation etc.). What will users have to be aware of when trying to register functional data? (which would be great in the future) I am not asking for a rigorous analysis here, but a short paragraph provided by these true expert authors would be highly useful.

Reply: We agree. Our revision includes a short discussion on this point, describing the expected kinds of tissue distortions due to fixation, dehydration, and clearing. We specifically point out the lack of "ground-truth" in this regard, and how novel technologies could help, as the reviewer points out.

1.5

The term "irrelevant sources of variability" needs some additional explanation /justification, especially in the light of the recent study by Linneweber et al. (2020, Science. 367(6482):1112-1119.) which relates morphological variability to behavioral individuality.

Reply: The revised manuscript provides more details in the section 5.5 ("Symmetry") clarifying this point, including a reference to the recent related work pointed out by the reviewer. To summarize the additions, we more directly state how analysis can be done to recovery asymmetries after registration to our (symmetric) template, and what analysis to avoid when analyzing asymmetries.

1.6

I agree that the SD of the Jacobian determinant is a highly useful measure for non-isometric shrinkage, as explained nicely in section 4.2.2.. It seems that Hessian matrix norm also measures precisely this, and the reported values for JSD and HMN correlate nicely (table 8). Does this mean that both provide a similarly good means for judging on non-isometric tissue shrinkage? I understood the text like this, but admittedly had some difficulties understanding these sections. It would be nice to explain this somewhat clearer to the non-expert, and maybe also include some judgement on what values become nearly unacceptable for registration of future samples.

Reply: We agree the reviewer that there is a need for more clarity on this point. We now more explicitly state our conclusions and recommendation in the main text in a single sentence. The supplement now includes some additional discussion and rationale regarding this topic.
